# Incorporating earned value management into income statements to improve project management profitability and elevate application in the business and management

**DOI:** 10.1371/journal.pone.0312956

**Published:** 2025-01-03

**Authors:** Rudy Setyopurnomo, Sudarso Kaderi Wiryono, Yuliani Dwi Lestari, Subiakto Sukarno

**Affiliations:** School of Business and Management, Bandung Institute of Technology, Bandung, Indonesia; Thamar University: Dhamar University, YEMEN

## Abstract

Our goal is to improve Project Management (PM) profitability and reduce discrepancies in information among stakeholders, which can result in conflicts. This will be accomplished by incorporating Earned Value Management (EVM) with the Income Statement and incorporating earnings before interest, tax, depreciation, and amortization (EBITDA) with the work breakdown structure (WBS). These incorporations will streamline information sharing between PM, other management professionals, and the stakeholders, ultimately fostering the application of PM within broader business and management contexts. We drew on theories from Project, Operations, Financial Management, Agency Theory, and the Theory of Performance Frontiers. We conducted multiple case studies in a shipbuilding corporation with 25 projects and an aircraft manufacturing corporation with 20 projects. Our analysis involved both qualitative and quantitative methods, ensuring the accuracy and practicality of our hypotheses and instilling confidence in our research findings. This integration addresses the information gap on profitability for non-project management professionals, empowering stakeholders to enhance project performance. This integration benefits the company by allowing the project manager and stakeholders to gain real-time insights into the project’s profitability, which enables continuous optimization of daily EBITDA by improving efficiency and productivity to achieve the project’s profitability target. This study and its innovative findings originate from the author’s unique approach, which is a significant contribution to the field of Project Management. This research focused on project management using EVM in for-profit organizations. Integrating EVM in the income statement also facilitates PM application in other management systems.

## 1. Introduction

Project management is a well-established field that deals with complex tasks within tight constraints [1, 2]. It is integral to modern management with strong theoretical foundations and scientific rigor [3–5]. Despite its importance, project management research receives little attention in leading business and management journals. The project management manifesto encourages collaboration between social scientists and urges business and management experts to make project research more relevant to society [3].

The use of EVM in project management has grown but is not widely accepted in business due to its limitations in addressing quality, uncertainty, and profitability [2, 3]. Current EVM indicators are limited and need enhancement. Profitability measurements are missing, creating a lack of real-time awareness among project managers and potential conflicts with stakeholders [4].

Upon analysis, it’s clear that EVM lacks profitability indicators. This hampers managers’ real-time understanding of project profitability, leading to reliance on financial reports later. This knowledge gap among managers can create conflicts. Our research suggests integrating EBITDA into EVM to maximize daily EBITDA in all processes, WBS, and work packages for improved performance and profitability.

Our study utilized multiple embedded case studies within complex defense industries in a shipbuilding corporation with 25 projects and an aircraft manufacturing corporation with 20 projects. We collected and analyzed qualitative data through NVivo interviews and quantitative data through SPSS surveys. Data collection took place over a longitudinal time horizon.

We aim to improve EVM by integrating it into the income statement and linking EBITDA to WBS and work packages to provide timely profitability information, enhance daily performance, and broaden the practical application of project management. This research has implications for real-world implementation in business and management.

RQ1. How can a company integrate EVM into Income Statement to improve Project Management Profitability and elevate application in the Business and Management?
RQ2. Is EBITDA the appropriate measure for assessing PM profitability?
RQ3. How can a company use integrated EVM-I/S to increase PM profitability and maximize PM daily EBITDA?

RQ4. What are the benefits of using EBITDA to measure PM profitability?
RQ5. How can a company enhance its ability to support and make strategic PM decisions using EBITDA?

This study focuses on both profit-driven and not-for-profit organizations, as both need to secure operational earnings for sustainable success. However, the study does not delve into the measurement of interest, taxes, depreciation, and amortization in project management, nor does it explore the role of project management in valuations such as mergers and acquisitions. The novelty of this research lies in integrating EVM into the income statement and EBITDA into the WBS, enhancing project management profitability and applying EVM–PM in other business and management fields, which is the author’s innovation. The integration of EVM into income statements and EBITDA into WBS not only combines the project management and financial systems but also integrates the language of both fields, enabling leaders to foster a strong corporate culture, improve efficiency, and enhance productivity for better performance and profitability.

The structure of the paper is as follows: Section 1, Introduction, and Section 2. Literature Review and Section 3 Research Methods. Then we will have Section 4, Result and Discussion, and finally, Section 5 is the conclusion.

## 2. Literature review

The upcoming section will provide a comprehensive review of how to enhance Project Management (PM) profitability and elevate PM applications in other business and management fields. Firstly, we will explore project-based firms (PBF), projects, and Earned Value Management (EVM), including components such as Work Breakdown Structure (WBS) and Work Packages. We will also delve into Agency Theory, which covers the roles of principals (Project Owners) and agents (Contractors) and how information gaps about profitability can lead to conflicts among stakeholders. Additionally, we will discuss the disparity between PM language and financial literacy, such as Earnings Before Interests, Taxes, Depreciation, and Amortization (EBITDA) among stakeholders, and its impact on information asymmetry using the theory of Productivity Frontiers.

**Fig 1:**
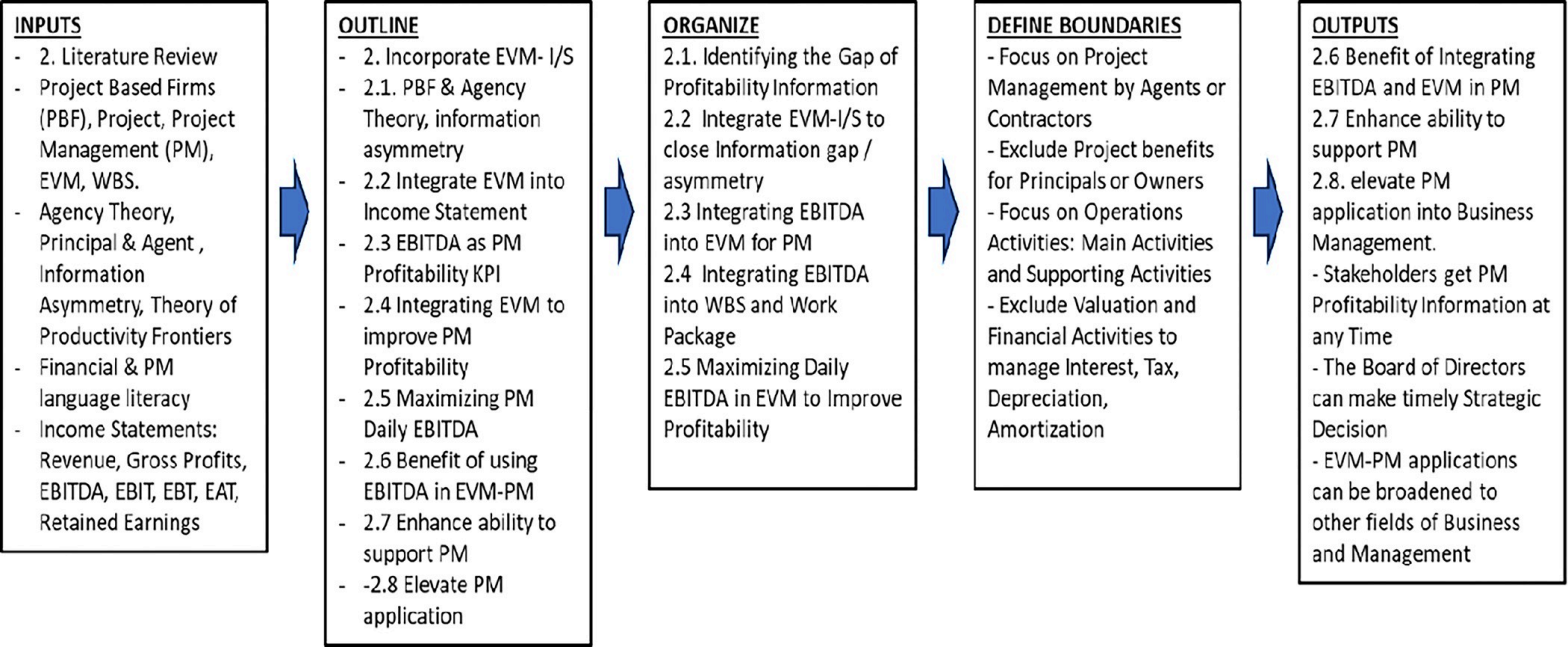
The five steps to incorporate PM EVM-income statements.

To address Research Question 1, we need to answer research questions 2, 3, 4, and 5 to understand the practical application of EVM in business management. Therefore, we will delve into topics such as Project Based Firm (PBF), Agency Theory, and Information Asymmetry in Section 2.1. Fig 1 visually presents the five steps to incorporate Project Management EVM with the Income Statement.

In section 2.2, our focus is on the integration of EVM into the Income Statement, a crucial step to improve Project Management profitability. In Section 2.3, we will explore the appropriateness of EBITDA as the Primary Profitability indicator of PM. Section 2.4 will discuss how to integrate EVM–Income Statement and EBITDA to enhance profitability, and Section 2.5 will delve into how to maximize PM daily EBITDA.

Furthermore, section 2.6 will discuss the benefit of using EBITDA in the EVM-PM. Section 2.7 will discuss enhancing EVM-Income Statement—EBITDA in the Work Breakdown Structure (WBS) to support PM. Additionally, we will organize the literature from section 2.1 until section 2.5 and define the boundary of the study that focuses on PM from the agents (contractors’) perspectives and exclude the discussion of project owner benefits that may result from the project usage after PM completion. We also exclude discussing managing interest, tax, depreciation, and amortization for corporation valuations.

Finally, in section 2.8, we will summarize how to elevate PM applications by integrating EVM with income statements and WBS—EBITDA into other business and management fields and summarize the hypothesis we built during the literature review.

### 2.1. Project based firm, project, project management and agency theory

Project management is a complex field that aims to deliver cost-effective, high-quality, timely, and safe products [4, 6–10]. Frontier analysis identifies best-in-class companies and areas for improvement, driving continuous improvement and providing a competitive advantage [11]. We will explore how to enhance the use of EVM beyond project management and traditional methods [4], to prioritize project management efficiency, productivity, and profitability from the outset.

#### 2.1.1. Project-based firms: Understanding agency theory and information asymmetry

Project management is crucial for project-based firms (PBFs) to enhance employee performance and execute projects effectively [2]. It extends to encompass internal and external stakeholders with diverse expectations [3]. Agency theory provides insights into inter-organizational relationships, highlighting the importance of aligning the principal’s and agent’s value propositions to achieve optimal project management outcomes [12]. Prioritizing diverse stakeholders and promoting transparent communication are essential. Information asymmetry arises when one party possesses information they do not openly share. However, such information asymmetry can give rise to opportunistic behavior [13, 14], typically exhibited by the agent [15] and sometimes by the principal [16].

### 2.2. Integrate EVM into income statements to improve project management profitability and elevate application in the business and management?

RQ1. How can a company integrate EVM into Income Statement to improve Project Management Profitability and

elevate application in the Business and Management?

To answer Research Question 1 (RQ1), we split it into three RQ as follows:

RQ1. 1. How can a company integrate EVM into Income Statement
RQ1. 2. How can a company use integrated EVM- I-S to improve Profitability
RQ1. 3. How can integrated EVM-Income Statement elevate application in the Business and Management?

Further we will study the literature to answer RQ1. 1 by studying seven layers of Income Statements (I/S) framework.

#### 2.2.1. Seven layers of income statement and management control

The income statement, also known as the profit and loss statement, is a vital financial reporting tool for businesses and management, providing crucial insights into costs, benefits, and operational profitability [17–21]. The seven-step income statement structure, depicted in Fig 1, serves as a valuable framework.

The process starts with revenue, direct operating costs, and gross profit, a key measure of operational profitability. However, it is important to note that gross profit does not account for indirect operating costs [22], and should not be the sole measure of operating profitability. On the other hand, EBIT (Earnings Before Interest and Taxes) includes all operating costs, including depreciation and amortization. Although depreciation and amortization are considered operating costs, they are not directly controlled by project managers but rather by the board of directors (BOD) [23, 24]. Thus, we do not recommend using EBIT as a profitability objective for daily project management activities, as daily depreciation and amortization are fixed costs not within the control of project managers.

EBITDA includes direct and indirect operating costs but excludes interest, tax, depreciation, and amortization. Company EBITDA is solely under company operational control, and PM EBITDA is under project managers’ control. EBITDA can be used to measure operational efficiency, productivity, and profitability [24, 25], making it a valuable key performance indicator (KPI) for project management. Our next step will be to delve into its suitability as a KPI in PM and investigate how to integrate EVM with the income statement framework.

#### 2.2.2. Integrating EVM project management with income statement

**Fig 2:**
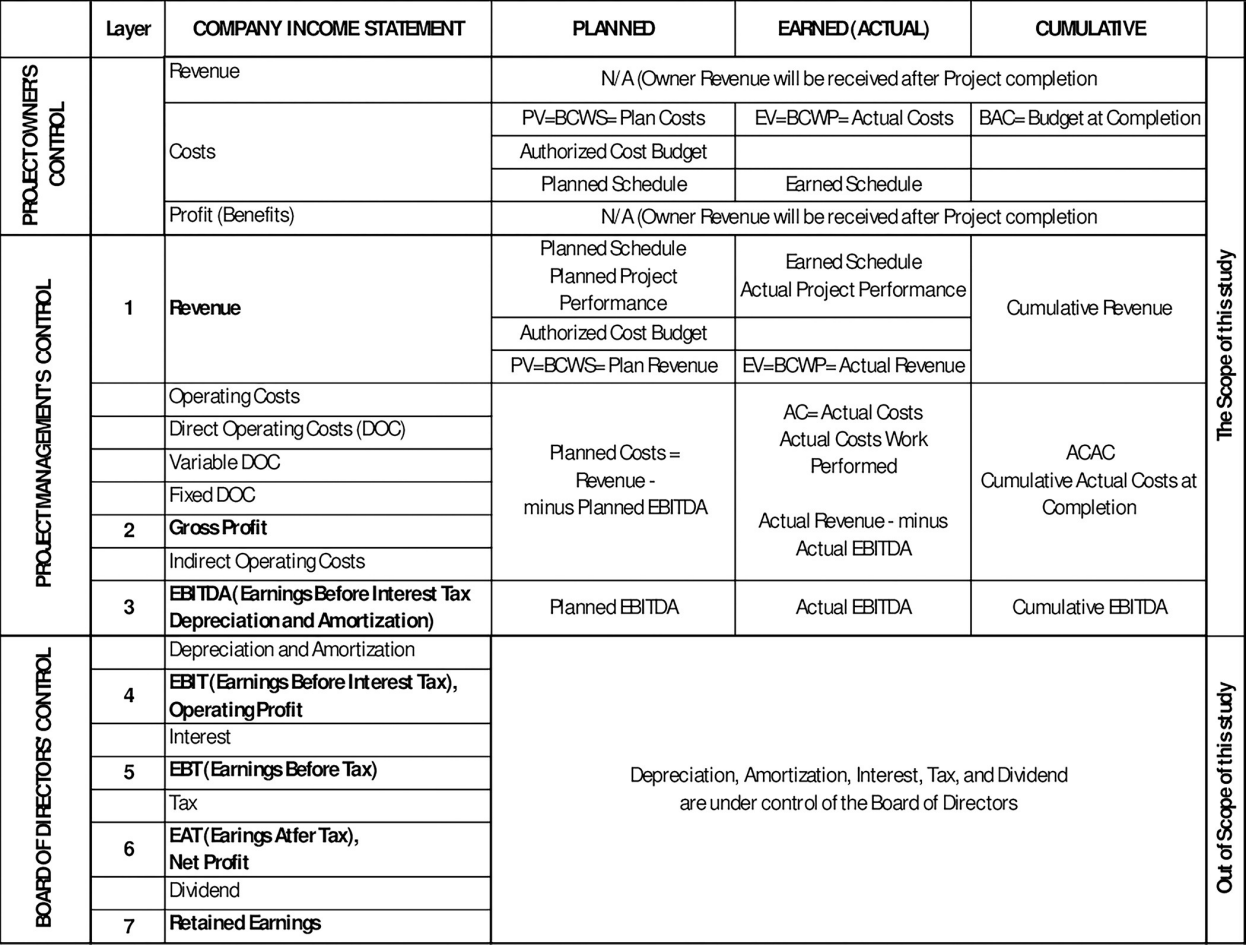
The seven layers of income statement, EVM, and its relationship with management control. (Author’s property).

It is essential to recognize the significance of PM profitability for its success, growth, and continual operation [26]. Thus, it is crucial to grasp the terminology of profitability measures in Earned Value Management (EVM) and align them with the profit and loss framework, as depicted in Fig 2. EVM is a comprehensive system that compares actual and budgeted work values, time, and costs. Through the integration of time and cost perspectives, EVM assesses schedule and cost performance, calculates variances, and forecasts total cost and duration [2, 7, 27–33].

Fig 2 illustrates several EVM indicators, including Planned Value (PV) as the "budgeted cost of work scheduled" (BCWS), Earned Value (EV) as the "budgeted cost of work performed" (BCWP), and Actual Costs (AC) as the "actual costs of work performed" (ACWP) [27, 29–32, 34, 35].

The Earned Value Management (EVM) method lacks profitability indicators, leading to information imbalances among stakeholders. Integrating profitability indicators into EVM will enhance transparency and support from stakeholders [18]. Additionally, the EVM method does not consider revenue, using the term "budget" instead, necessitating the inclusion of a baseline for production and profitability [26]. The interpretation of Planned Value (PV) varies based on perspective. For effective management of profit-driven organizations, it is crucial for a contractor’s planned cost to differ from the planned revenue received from the owner. Setting a zero-profitability target may not motivate project managers to prioritize profit, and an unclear buffer between revenue and cost may lead to cost overruns.

Project managers (PMs) can utilize EBITDA as a buffer between revenues and costs to address this issue. This buffer can be represented by the equation: Planned cost = planned revenue minus planned EBITDA. By integrating the EBITDA target as a profitability goal, the project manager can effectively manage profitability and intervene early if necessary [33, 36]. Moreover, incorporating buffer into the EVM system can positively impact profitability [37]. In Fig 2, we illustrate the integration of EVM into the Income Statement and the incorporation of EBITDA into the framework. Incorporating EBITDA into EVM can streamline accounting processes and enable direct project performance management. However, it’s important to evaluate the relevance of using EBITDA as a KPI in project management before implementation [38, 39]. Within this framework, our objective was to verify the suitability of EBITDA as a KPI in PM.

### 2.3. EBITDA as a key performance indicator for project management

In addressing the second research question, we explore the significance of profitability for project management activities and introduce some terminology related to operational profitability.

**Fig 3:**
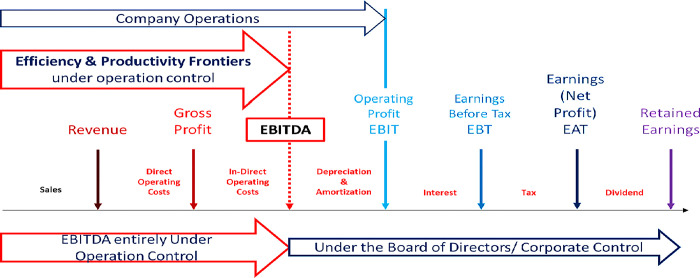
Company earnings and their relationship with the management authority and control (Author’s property).

Fig 3 vividly illustrates the seven crucial steps of an Income Statement. It begins with sales activity, effectively generating revenue. The next step involves the Company’s core operations, which consume direct operational costs, encompassing both variable and fixed costs, culminating in the creation of gross profit. The third step involves the Company Supporting Units engaging in Indirect Operations activities, such as General Administration, and consuming Indirect Operating Costs resulting in EBITDA (Earnings Before interest, depreciation, and amortization). Finally, the decision to expense Depreciation and Amortization by the Board of Directors leads to EBIT (Earnings Before Interests and Tax), also called Operating Profit [25, 40].

Noticeably, the Company’s operations management authority is confined to specific operational expenses, as control over Depreciation and Amortization expenses rests with the Board of Directors. The decision to account for Depreciation, Amortization, Interest, and Tax falls within the Board of Directors’ purview, limiting the Operations Management’s jurisdiction to EBITDA. Consequently, operations management has no complete control over operating profit expenses because it excludes depreciation from its jurisdiction. The ultimate earnings that operations management can deliver is EBITDA [25, 40].

The frontier approach assesses performance using managerial benchmarks and theoretical perspectives. The production frontier represents the highest level of performance achievable by an operational unit based on its operational decisions (Vastag, 2000). EBITDA can be a critical operational management performance indicator (KPI), representing the profitability frontier under their direct control.

In addition, Fig 3 illustrates the correlations between earnings metrics, such as Earning Before Tax (EBT), Earnings After Tax (EAT) or Net Profit, and Retained Earnings, and management authority and control. This illustration highlights the essential role of operational management in achieving profitability and control [25, 40].

Critics of EBITDA as a company indicator argue that it is an inadequate measure for evaluating investment activities since it requires consideration of other indicators and is not a reliable proxy for net cash flow [41]. Additionally, some argue that EBITDA is insufficient for guiding financial strategies and valuation decisions such as bankruptcy, mergers, and acquisitions [42–45].

On the other hand, advocates of EBITDA as an indicator maintain that it is a suitable measure of a company’s operations and project management profitability, reflecting the ultimate profitability that only operations management and project management can achieve [41, 46–50]. A Key Performance Indicator (KPI) is a crucial metric used to gauge the achievement of critical objectives and is essential for understanding and enhancing performance [51].

*EBITDA is a performance metric that assesses a company’s profitability by excluding factors such as taxes, interest, and debt costs [23, 52]. It is a reliable indicator of operational effectiveness and is well-suited as a profitability metric for evaluating operational management [53].*

It’s important to align performance dimensions with company practices such as knowledge management, openness, and sustainability [54]. Performance measurement involves assessing effectiveness and efficiency using specific measures [55]. EBITDA is crucial for operational management and integrating it into earned value management (EVM) can help project managers plan and control financial targets. An effective performance management system should incorporate a limited number of KPIs to provide a comprehensive view of company performance and prevent information overload [55]. Integrating EBITDA into EVM allows project managers to plan and control financial targets effectively.

Based on this review, we establish the following hypotheses:

Hypothesis 1. A: EBITDA is a proper measure of project management profitability because it excludes interest, tax, depreciation, and amortization, which do not fluctuate according to variations in project management efficiency and productivity.
Hypothesis 1. B: EBITDA is a proper measure of project management profitability because it is entirely within the control of the project manager and the team.
Hypothesis 1 C: EBITDA is an appropriate measure of operational Profitability for implementing company operations and project management.

### 2.4. Integrating EBITDA into EVM- income statements to manage and improve project management profitability

In this section, we will delve into integrating EBITDA into EVM for project management, which addresses the third research question. Our focus will be on exploring the incorporation of EBITDA measures to maximize daily EBITDA within EVM, and we will pose two important questions:

1. Can integrating EBITDA as a target in business processes and WBS enable active profitability management?

2. Can integrating EBITDA as a target in business processes and WBS streamline operations and project management while increasing profitability?

To address these inquiries, we conducted a comprehensive analysis of various EVM performance indicators and devised a strategy for integrating EBITDA into the current system. EBITDA is crucial as a performance metric not only to gauge performance but also to facilitate the formulation of improvement initiatives [54].

Performance indicators play a vital role in enabling companies to evaluate their systems’ adherence to standards and progress toward specific objectives [56]. Using key performance indicators such as EBITDA empowers managers to adeptly guide the company towards enhancement, make well-informed decisions, and incentivize and reward employees. With technological advancements, it is now feasible to collect data and analyze the most pertinent variables in real time to assess key performance indicators [51].

**Fig 4:**
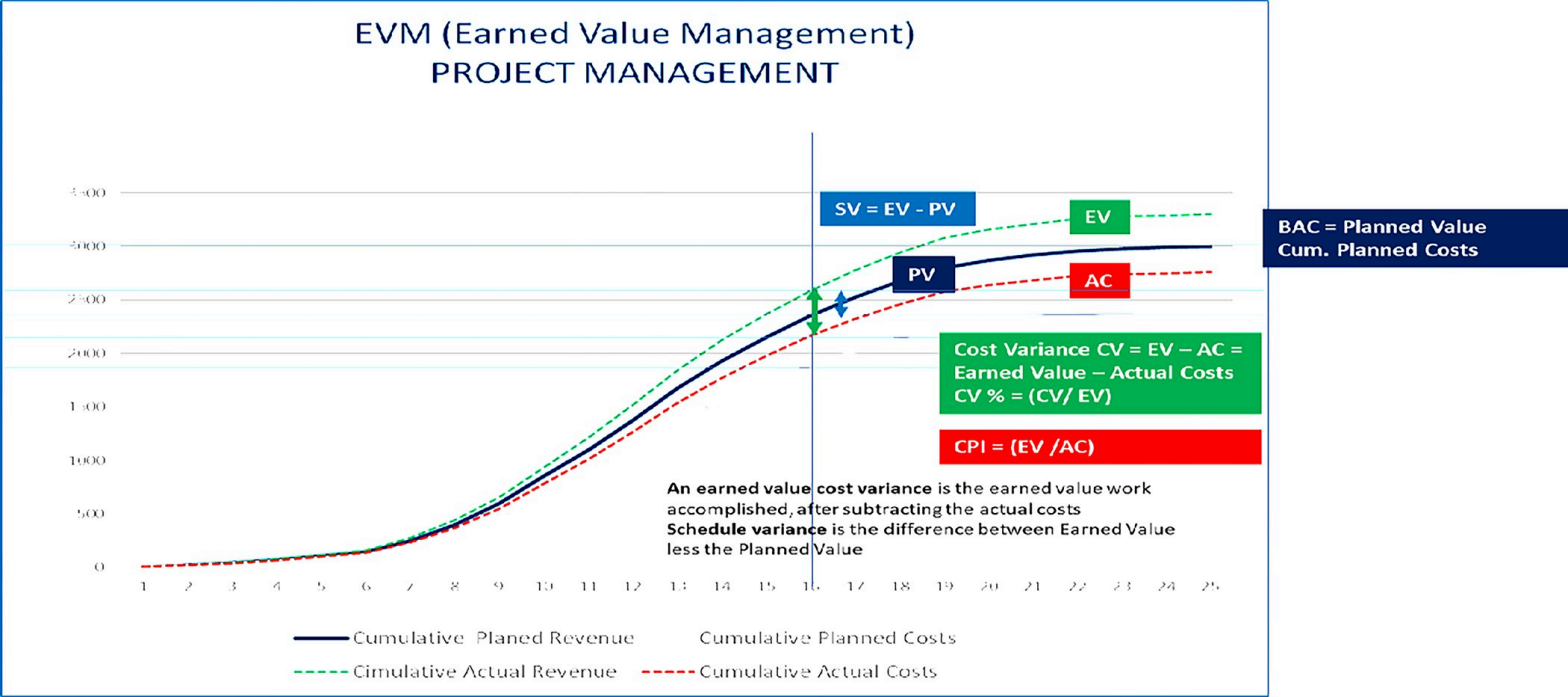
S-Curve EVM project management from a project owner’s perspective (Author’s property).

Fig 4 depicts the S-Curve EVM project management from the project owner’s viewpoint, wherein PV represents the Planned Value equivalent to the Planned Costs.

The Budget Actual Costs (BAC) represent the accumulated planned expenses for a project. The Earned Value (EV) is subtracted from the Actual Costs (AC) to calculate the cost variance. Meanwhile, the Schedule Variance (SV) compares the Earned Value (EV) and Planned Value (PV) against the project baseline and work schedule. It’s worth noting that EVM relies on monetary values instead of schedule indicators, which some professionals may find unreliable [32, 57].

This study focuses on examining the financial metrics utilized to manage project management profitability. However, the current version of the Earned Value Management (EVM) framework lacks complete oversight over production and revenue. Therefore, it is necessary to establish an additional baseline to ensure better control over these aspects [26].

**Fig 5:**
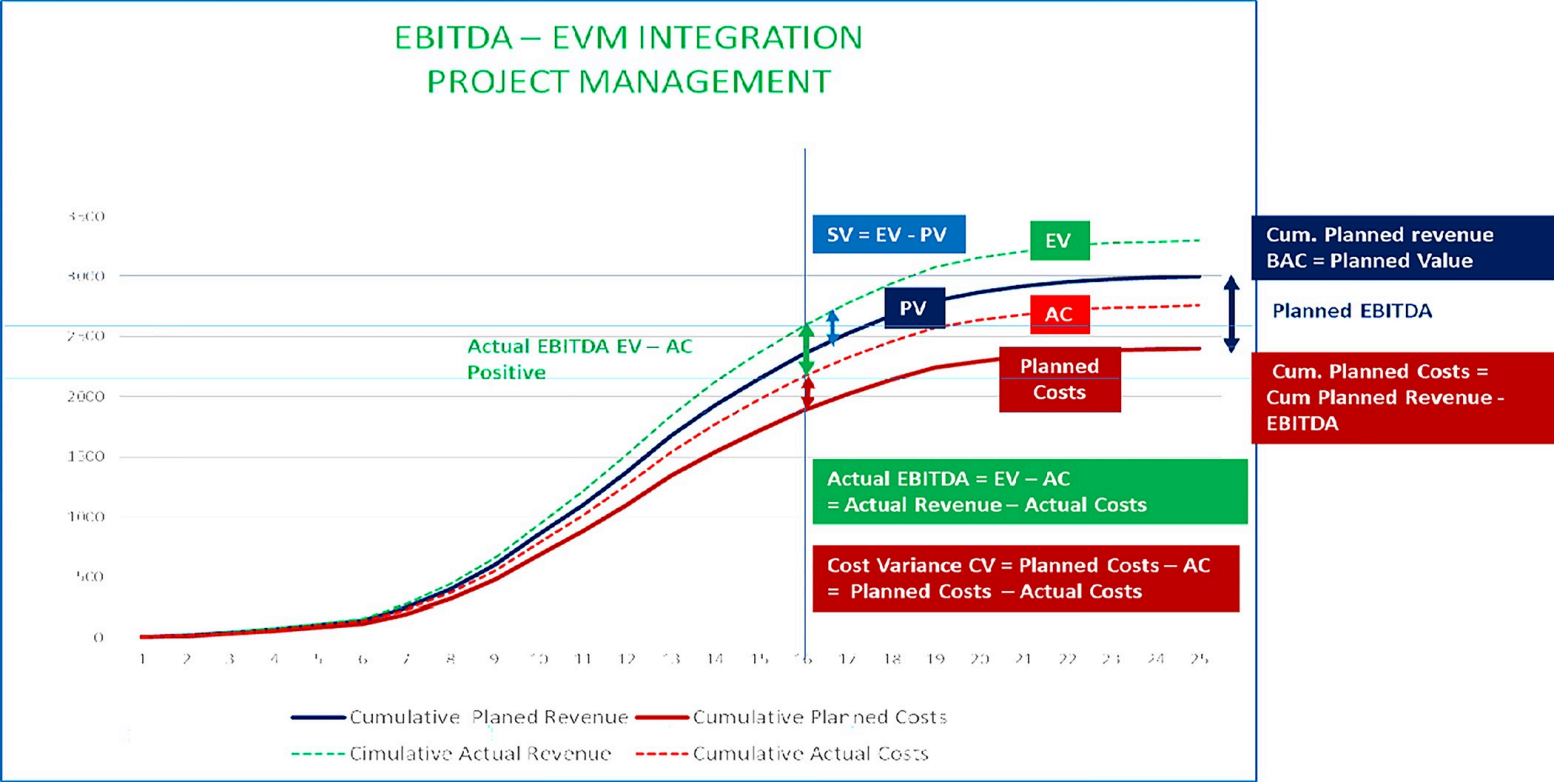
S-Curve EBITDA–EVM integration in project management with a positive actual EBITDA (Author’s property).

EBITDA and EVM can be integrated into project management for a positive outcome. The project manager can use EBITDA as a buffer to manage actual costs effectively. Based on Fig 5, the EBITDA figure is positive, which suggests that the revenue surpasses the costs incurred (EV-AC). In project management, cost variance is the difference between planned and actual costs, denoted as CV (PM) = planned costs—AC. Moreover, schedule variance (SV) is the difference between the work performed and the scheduled planned value (SV = EV—PV). We must integrate these metrics into each WBS and work package to include EBITDA and EBITDA Margin in the EVM.

#### 2.4.1. Integrating EBITDA into WBS and work packages

Numerous companies encounter difficulties when implementing budgets [58]. The literature emphasizes that performance measurement is essential for effectively managing production systems [56]. Inaccurate data can hinder companies in assessing profitability and recognizing areas for improvement [54]. Financial indicators like EBITDA are important for demonstrating the process’s financial performance to management. Developing key performance indicators (KPIs) such as EBITDA would enable stakeholders to have visibility into the process’s real-time performance [59]. Monitoring real-time product costs is crucial for promptly identifying any unexpected increases that could erode the EBITDA margin [51].

The Work Breakdown Structure (WBS) serves as a framework for outlining the project budget [30, 60, 61]. EBITDA can be assigned to each WBS and Work Package using a top-down approach, establishing target EBITDA and refining these allocations based on bottom-up input. By combining bottom-up and top-down approaches, governance challenges can be addressed effectively, and learning within the project management process can be promoted. The allocation of EBITDA to every WBS provides a profitability indicator for each business process. Companies can apply Business Process Reengineering (BPR) to reduce activity costs by analyzing and redesigning WBS workflows and processes [62]. Therefore, integrating EBITDA into the WBS facilitates the implementation of BPR in project management, ultimately strengthening profitability.

### 2.5. Maximizing PM daily EBITDA in the integrated EVM-I/S system to improve PM performance and profitability

In addition to the result of the study in section 2.4 we will further study to addresses the third research question

RQ3. How can a company use integrated EVM-I/S to increase PM profitability and maximize PM daily EBITDA?

To maximize daily EBITDA, project managers need to focus on improving cost efficiency, productivity, quality, delivery, and safety [13, 63, 64]. The first step is to provide structured indicators to assess whether a production system efficiently uses its resources [56]. Maximizing daily EBITDA involves conducting daily simulations [65], setting high expectations, utilizing objective data, and providing timely feedback on performance [66]. Strategies like Business Process (BP) methods [11], and the Project Value Risk Opportunity (PVRO) framework can help achieve high-performance positions and optimize decision-making [4, 67, 68]. Companies can maximize EBITDA by elevating the constraint through lean methods in business process management, which involves identifying customer value and addressing wastage to resolve bottlenecks. Companies can maximize EBITDA by elevating the constraint by increasing their capacity with extra resources [69]. Addressing wastage can resolve bottlenecks in the process, leading to improvements [59]. The superficial differences in Business Processes improvement may significantly impact project success [62].

In summary, we define the following hypotheses:

Hypothesis 2. A. The Company can maximize EBITDA with EVM in PM by integrating EBITDA into WBS and applying the target EBITDA margin to every WBS and work package.
Hypothesis 2. B. The company can maximize daily EBITDA by integrating EBITDA as a target in business processes and WBS enables project managers to manage profitability actively.
Hypothesis 2. C. The Company can maximize daily EBITDA by integrating EBITDA as a target in business processes and WBS allows project managers to increase profitability.

### 2.6. The benefit of using EBITDA to measure PM profitability in integrated EVM-I/S system

This section answers the fourth research question: What benefits does using EBITDA to measure PM profitability offer? Through a literature review, we identified the limitations of EVM implementation [6], and how integrating EBITDA can mitigate these issues:

#### 1. EVM does not differentiate between critical and noncritical activities

It is crucial to integrate EBITDA into each Work Breakdown Structure as it enables a company to establish an internal control system for guiding resource allocation decisions based on WBS EBITDA or EBITDA Margin. These internal control systems are essential for managing companies with multi-operation production systems, particularly in the context of organizations overseeing multiple manufacturing facilities. The introduction of divisional and departmental budgets plays a key role in overseeing daily operations and comparing production and costs across different divisions and departments [65]. Integrating EBITDA into WBS, the company can differentiate the critical and non-critical activities in Project management.

#### 2. EVM does not indicate dependency among activities

Project managers are required to work closely with other departments to effectively manage EBITDA and meet EBITDA targets. Coordination involves integrating a series of interdependent tasks that are essential for organizational productivity and efficiency [70–72]. Cooperation is vital for the successful implementation of complex strategies, relying on coordinated efforts and information sharing [71, 73]. Therefore, integrating EBITDA into EVM will allow for better visualization of the interdependency among activities. Achieving target EBITDA requires improved cooperation, coordination, and interdependency among activities.

#### 3. EVM does not include behavioral management aspects

Maximizing daily EBITDA hinges on improving behavior and engagement. Engagement refers to involvement, commitment, and the willingness to overcome obstacles or constraints in strategy execution, ultimately challenging these constraints and delivering better performance [70, 74–76]. Therefore, integration of EBITDA into WBS will simplify manager to manage profitability.

#### 4. High information requirements

Ensuring maximum daily EBITDA across all work breakdown structures and work packages requires effective communication. Precise and efficient communication is vital for sharing strategic information and facilitating successful implementation [72, 75, 77]. Thus, integrating EBITDA as a target in business processes and work breakdown structures will provide project managers with the necessary information. Therefore, integrating EBITDA as a target in business processes and work breakdown structures will enable the project manager’s ability to get the information needed.

The above findings reveal that integrating EBITDA as a business process target and WBS enables project managers to improve daily profitability. The integration simplifies the project managers’ ability to demonstrate daily profitability (to avoid information asymmetry). In addition, the integration enables support project manager in achieving successful strategy and PM Target.

Based on our findings, we formulated the following hypothesis:

Hypothesis 3. A: Using EBITDA to measure PM profitability allows project managers and teams to actively manage and maximize PM profitability.
Hypothesis 3. B: Applying EBITDA to EVM and WBS enables companies to display and control daily Project Management profitability.
Hypothesis 3. C: Using daily EBITDA as a target in business processes and Work Breakdown Structure (WBS) eases operational and the project management and allows them to display and control daily profitability.

### 2.7. Enhancing the ability to support and make strategic decision

Building on the previous findings, we are now addressing research question 5: How can a company optimize its support for strategic PM decisions using EBITDA?

Evaluating project success is challenging, as different stakeholders have varying definitions of success. To address this, it’s important to consider the perspectives of the owner, contractor, end user, and the public. While measuring management performance is crucial, financial results often lag behind decision-making, posing challenges for taking timely action [55].

To prevent delays in accessing profitability information, it is recommended to monitor daily EBITDA. By sharing profitability information and displaying planned and actual daily EBITDA, the company can promote transparency and enhance its capabilities in this area. This approach fosters effective communication, supports knowledge management, and reinforces the significance of project management [17, 20, 78].

The equitable distribution of information has a positive impact on both individual and organizational performance. Effective communication by project managers significantly enhances the performance of project teams, as managers dedicate considerable time to communicating with team members and key stakeholders [79]. Sharing information can facilitate manufacturing decisions while visualizing and communicating efficiency and productivity can assist in setting goals and driving performance improvement [80].

The use of EBITDA indicator in every WBS enables PM to indicate deviations and streamline our business processes. This method helps us visualize and evaluate capacity across all processes, synchronize operational capacity, and identify bottlenecks [81, 82].

It is essential to communicate clearly and effectively when sharing information about EBITDA results, including the target, strategy, and implementation. This clear communication is crucial for ensuring that everyone understands the information and can successfully execute the strategy [70, 72, 77].

By summarizing this discussion and those in the previous subsections, we developed the following hypotheses:

comprehension of the concept after eleven months of research in the longitudinal time frame.

Hypothesis 4. A: A company can improve its ability to support PM by sharing the daily target and actual EBITDA result with its supporting unit’s management.
Hypothesis 4. B: A company can improve its ability to support PM by sharing the daily target and actual EBITDA with the board of directors to provide timely strategic decisions to the project manager.
Hypothesis 4. C: A company can improve its ability to support PM by sharing the daily Target and Actual EBITDA with the supporting unit’s management and Board of Directors to gain support and provide timely strategic decisions to Project management.

From the above study, we can summarize that integrating EVM—Income Statements -WBS—EBITDA can broaden the application of EVM in management and business fields.

Further, we will provide a summary table to ease the test of the hypotheses in the next Section.

### 2.8. Summary of literature study and hypothesis development

To answer RQ1, we summarize the finding from previous study and hypothesis in Table 1.

**Table 1:** Summary of Hypothesis development.

	Batch #1	Batch #2
**RQ1. How can a company integrate EVM into I-S to improve Project Management Profitability and elevate application in the Business and Management?**		
1. How can a company integrate EVM into Income Statement		
2. How can a company use integrated EVM- I-S to improve Profitability	V	V
3. How can integrated EVM-Income Statement elevate application in the Business and Management?	V	V
**RQ2. Is EBITDA the appropriate measure for assessing PM profitability?** 1. EBITDA is the appropriate measure of Project management Profitability.		
Hypothesis 1. A: EBITDA is a proper measure of project management profitability because it excludes interest, tax, depreciation, and amortization, which do not fluctuate according to variations in project management efficiency and productivity.	V	
Hypothesis 1. B. EBITDA is a proper measure of project management profitability because it is entirely within the control of the project manager and the team.	V	
Hypothesis 1 C: EBITDA is an appropriate measure of operational Profitability for implementing company operations and project management.		V
**RQ3. How can a company use integrated EVM-I/S to increase profitability and maximize PM EBITDA?**		
Hypothesis 2. A. The Company can maximize PM EBITDA that uses EVM by integrating EBITDA into WBS and applying the target EBITDA margin to every WBS and work package.	V	
Hypothesis 2. B. The company can maximize daily EBITDA by integrating EBITDA as a target in business processes and WBS enables project managers to manage profitability actively.		V
Hypothesis 2. C. The Company can maximize daily EBITDA by integrating EBITDA as a target in business processes and WBS allows project managers to increase profitability.		V
**RQ4. What are the benefits of using EBITDA to measure PM profitability?**		
Hypothesis 3. A. Using EBITDA to measure PM profitability allows project managers and teams to actively manage and maximize PM profitability.	V	
Hypothesis 3. B. Applying EBITDA to EVM and WBS enables companies to display and control daily Project Management profitability.	V	
Hypothesis 3. C. Using daily EBITDA as a target in business processes and Work Breakdown Structure (WBS) eases operational and the project management and allows them to display and control daily profitability.		V
**RQ5. How can a company enhance its ability to support and make strategic PM decisions using EBITDA?**		
Hypothesis 4. A. A company can improve its ability to support PM by sharing the daily target and actual EBITDA result with its supporting unit’s management.	V	
Hypothesis 4. B. A company can improve its ability to support PM by sharing the daily target and actual EBITDA with the board of directors to provide timely strategic decisions to the project manager.	V	
Hypothesis 4. C: A company can improve its ability to support PM by sharing the daily Target and Actual EBITDA with the supporting unit’s management and Board of Directors to gain support and provide timely strategic decisions to Project management.		V

Table 1 provides an overview of the research questions, corresponding hypotheses, planned data collection, and analysis for Batch #1 and #2. The second round of data collection and analysis will be carried out to assess the comprehension of the concept after eleven months of research in the longitudinal time frame.

In reference to the study discussed in Section 2, it was concluded that incorporating earned value management (EVM) into income statements has the potential to improve project management profitability and increase its significance in the business and management fields. This research is a step towards wider acceptance of EVM in these domains. Furthermore, we will delve into the hypotheses in the upcoming section.

## 3. Methods

The authors have confirmed that this study was reviewed and approved by the institutional review board (ethics committee) of the Bandung Institute of Technology prior to commencement. The original approval document was obtained from our academic institution’s ethics committee. The author and the author’s institution have obtained permission from the companies to conduct research for academic purposes and have been authorized to produce academic literature. The ethics statement document includes the written permit (approval) for research within the companies. The researcher/interviewer conducted the study without any prior relationship with the participants. Regarding participant consent, the author and the companies informed the participants that the study was purely for academic purposes. The research participants are company officials and management; no minors were included. The authors commenced research in Shipbuilding on May 15, 2023, and concluded it on July 12, 2024. Additionally, they initiated research in Aircraft Manufacturing on May 22, 2023, and completed it on July 19, 2024.

To explore the connection between EVM and profitability, we conducted a comprehensive literature review using Science Direct, Google Scholar, and Publish or Perish with the keyword "Earned Value Management." Initially, our search resulted in over 1000 articles. We then refined our search by adding the keyword "profit" and setting the publication date from 2014 onwards, which narrowed the relevant articles to 108. Using NVivo, we meticulously analyzed these articles to identify word frequencies. Our analysis revealed that "project = 15.754," "earned = 3.226," "EVM = 3.390," and "costs = 1.068" were frequently mentioned, while "revenue = 66," "profit = 300," and "profitability = 53" appeared less often. Overlooking these critical aspects could lead to information imbalances and conflicts among stakeholders, underscoring the necessity of integrating profitability into EVM for effective project management [78]. The table of this findings is available in S1 Appendix. Later in this chapter, our research indicates that EBITDA is the optimal profitability frontier for project management [67] and can serve as a suitable indicator for PM. By integrating EBITDA into EVM methodology, we can maximize daily EBITDA in every business process, WBS, and work package to enhance PM performance and profitability.

Our research aims to create practical solutions through a pragmatic philosophy. Our methodology combined deduction and induction, and we developed a protocol to guide our research process [83]. We collected qualitative data through interviews and observation, while quantitative data was collected through online surveys. We employed a Multiple Embedded Case Study design. The selection was imperative to examine project complexity factors from an unbiased and comprehensive perspective [84]. Our analysis involved using SPSS for statistical analysis and NVivo for qualitative analysis. The Case Study Protocol and Consolidated Criteria for Reporting Qualitative Research Checklist are available in S2 and S3 Appendices [83, 85].

The corresponding author conducted the interview or focus group. The author has 25 years of experience as a management consultant and is considered an expert in turnaround strategies. The participants know the interviewer as the company’s Turnaround Expert. The interviewers’ genuine interest in the research topic and enthusiasm about conducting a case study that combines long-term practical experience with theory is evident. The study focused on understanding profitability information asymmetry in Project Management using EVM systems. They are eager to share knowledge with academic and practical communities.

The authors of this study delved into relevant theories and conducted surveys to comprehensively explore the effects of information asymmetry on profitability in Project Management, specifically through the use of EVM systems. They found that this information imbalance could potentially lead to conflicts between management and stakeholders.

To gain a deeper understanding, the study employed both qualitative and quantitative data collection and analysis, utilizing multiple embedded case studies and surveys. The research focused on Shipbuilding and Aircraft Manufacturing Companies that integrated EVM with Income Statements and EBITDA into their Work Breakdown Structure (WBS) to improve Project Management Performance and Profitability. Specifically, the study included two project-based organizations in the defense industry.

The authors conducted detailed case studies on two companies that integrated EVM with Income Statements and EBITDA into their WBS. The first company, a prominent shipbuilding company, managed 25 projects, including warships and submarines, while the second company, an aircraft manufacturing company, oversaw 20 projects, including helicopters and rockets. The study included the collection of both qualitative and quantitative data. Qualitative research methods, such as interviews and purposive sampling, were utilized. Invitations were extended to 30 individuals from Shipbuilding and Aircraft Manufacturing Companies, and 20 participants from various levels within the organizations were selected to take part. Regrettably, ten individuals were unable to participate due to time and scheduling constraints. Additional qualitative data was gathered through observations at aircraft manufacturing and shipbuilding companies, as well as through meeting minutes from the corporate office.

The research purpose was to communicate to the interviewees through various means, including face-to-face interviews, emails, WhatsApp messages, and phone calls. The company’s Board of Directors and employees were involved in the survey and interviews. The researcher obtained qualitative data from the corporate office through observations, interviews, and meeting minutes. During the interviews, only the interviewer and the interviewees were present. However, during observations and group discussions, non-participants who were company employees not assigned to the project were in the room.

The researchers provided a question guide and survey questionnaires for the structured interviews. For the unstructured interviews, the interviewees freely expressed their opinions about the program. Repeat interviews with the same person were not conducted. Structured interviews involved asking the same standardized questions to different interviewees. Before recording, the author informed the interviewees that the interview would be recorded and obtained their consent. During the interviews, the author used audio-visual recording via Zoom to gather data, enabling the taking of meeting minutes during the session. Interviews and discussions typically lasted 30–60 minutes, and participants received transcripts for review.

For Interviews, we sent thirty invitations to two companies, ten people unable to participate due to time and schedule constraints. As shown in Table 2, the author held two sessions of interviews with 20 volunteers representing various positions, including board members, senior executives, managers, project managers, and professionals from the PM team. The interview participants are as follows: one COO (Male, master’s degree); one CFO (Male, master’s degree); one EVP (Male, a master’s degree); nine V.P. (1 Male with a Doctorate, 1 Female with a master’s degree, 4 Males with a master’s degree, 3 Males with a bachelor’s degree); and eight Managers (1 Male with a master’s degree, 3 Females with bachelor’s degrees, 4 Males with bachelor’s degrees).

**Table 2:** Demographic data of interviewees.

Gender	Male			Female		
Degrees	Doctor	Master	Bachelor	Doctor	Master	Bachelor
Board of Director		1.C.O.O. 1.C.O.O				
E.V.P		1.E.V.P				
V.P.	1. V.P.	4. V.P.	3. V.P.		1.V.P.	
Managers		1.Manager	4.Manager			3.Manager

The first session consisted of structured interviews with predetermined questions. We distributed research protocols before the questionnaires to ensure the validity and reliability of our study [83, 84].

In the second session, we conducted unstructured interviews to delve deeper into participants’ understanding of integrating EBITDA with EVM and its usefulness in project management for their company. We specifically focused on the benefits of measuring PM EBITDA for project managers and stakeholders [78]. We used a longitudinal time horizon to understand the development of business processes and cultural improvement over time, which is valuable for researchers and project managers [2].

The researcher identified the themes in advance, but the author did not discuss data saturation in this qualitative research. The author used NVivo software to analyze the qualitative data from the interviews. During the project/program briefing, the participants were provided feedback on the findings.

In addition, we combined the case study with the survey method to create a comprehensive mixed-methods research design [83]. The author collected data from two separate participant batches from shipbuilding and aircraft manufacturing companies for quantitative data. We conducted our survey in two batches, with 220 volunteer participants (104 Shipbuilding participants, and 116 Aircraft Manufacturing Participants) surveyed over six months to test Hypotheses.

As shown in Table 3, during the second batch, we involved 125 volunteer participants (from both companies) over 11 months to test hypotheses A, B, C, and D. Our empirical data was collected using questionnaires scored on 7-point Likert scales and was quantitatively analyzed using SPSS. The researcher used the participants’ quotations to explain the findings from the interview results. The participants conveyed their perspectives using standard daily language, adding depth and authenticity to the research. This use of daily language made the findings more transparent and more relatable by showcasing how they were grounded in the participants’ actual words.

**Table 3:** Demographic data of survey respondents.

	POSITION					
Batch #1 Survey Responses	Board of Directors	Senior Managers	Managers	Project Management Team	Staff Functional Units	TOTAL
Ship Building Company	1	16	40	41	18	116
Aircraft Manufacturing Company	3	28	26	28	19	104
						220
Batch #2 Survey Responses	Board of Directors	Senior Managers	Managers	Project Management Team	Staff Functional Units	TOTAL
Ship Building & Aircraft Manufacturing Companies	1	9	54	35	26	125

The presented data and the findings were consistent, and the research findings highlighted the major themes. The researchers described some cases and discussed the minor themes. For instance, the application of EBITDA in Project Management in shipbuilding varies slightly from that of a ferry company to that of an aircraft manufacturer due to different corporate cultures.

## 4. Results and discussions

Referring to section 2.1.3, Fig 2 shows the integration of the income statement’s structure, EVM, and its relationship with management control.

Our research findings from section 2 indicate that companies can integrate EVM into overall business management by aligning it with income statement metrics. We propose expanding the use of EVM beyond project management. Project managers can incorporate EBITDA into every WBS to facilitate the direct translation of EVM indicators into financial reporting language and establish a relevant profitability metric for project management. To validate these conclusions, we comprehensively analyzed quantitative and qualitative data to address all remaining research questions.

### 4.1. Quantitative data collection and analysis proportion testing

#### 4.1.1. First batch of data analysis

In our initial data collection, we analyzed 220 questionnaires using IBM-SPSS to examine the use of EBITDA in project management profitability. We also investigated how a company can maximize daily EBITDA project management in the EVM system and the benefits of using EBITDA to measure project management profitability. This analysis used the following abbreviations: AMC means aircraft manufacturing corporation and SBC means shipbuilding corporation. Supporting Information for the First Batch of data analysis is available in S4 Appendix.

Proportion Testing

Hypothesis

Research Question 2: Is EBITDA the proper measure for project management profitability?

Hypothesis 1.A:

EBITDA is the proper measure of Project Management Profitability because it excludes Interest, Tax, Depreciation, and Amortization, which do not vary due to Project Management efficiency and productivity variation.

Hypothesis 1. B:

EBITDA is the proper measure of project management profitability because it is entirely under the control of the project manager and team.

Research Question 3: How can a company maximize daily EBITDA project management in the EVM system?

The Company can maximize daily EBITDA project management in the EVM system by integrating EBITDA with EVM and applying target EBITDA margins in every work breakdown structure and work package.

Hypothesis 2.A:

The Company can maximize EBITDA with EVM in Project Management by integrating EBITDA to WBS and applying target EBITDA margin in every work breakdown structure and work package.

Research Question 4: What are the benefits of using EBITDA to measure project management profitability?

Hypotheses 3.A:

Using EBITDA as a measure of Project Management Profitability allows Project Managers and Teams to manage and maximize Project Management Profitability actively.

Hypothesis 3.B

Applying EBITDA to EVM and WBS enables companies to display and control Project Management Profitability daily

Research Question 5 How can a company improve its ability to support and make strategic decisions for Project Management using EBITDA?

Hypotheses 4.A:

The Company can improve its ability to support Project management by sharing the daily Target and Actual EBITDA result with its supporting Units.

Hypothesis 4.B:

The Company can improve its ability to support Project management by sharing the daily Target and Actual EBITDA with the Board of Directors to provide timely strategic decisions to Project management

Chi square Testing

This analysis technique belongs to non-parametric statistics, which is a branch of statistics that does not rely on the assumption of a particular distribution of the data. It is often used when the data does not meet the assumption of normality or when the sample size is small. This method is more flexible and can be applied to ordinal data or data that is not measured at intervals.

Hypothesis

**H**_**01**_: π ≤ 0.70

**Ha**_**1**_: π > 0.70

The test statistics used in the population proportion test Chi square test $\chi 2=\frac{{\left(x-E\right)}^{2}}{E}$

$\hat{p}=\frac{x}{n}$, the proportion of people who answered 6 or 7 to the question, and po = 0.70. The decision-making rule was to reject Ho if *χ*2>*χ*2 table with α = 0.05

Table 4 displays the Hypothesis Testing Results.

**Table 4:** The first batch hypothesis testing results.

No	RESEARCH QUESTIONS	Hypotheses	AMC (n = 104)				SBC (n = 116)			
			$\hat{p}$	*χ*2- value	p-value	Conclusion	$\hat{p}$	*χ*2- value	p-value	Conclusion
**1**	RQ2. Is EBITDA the proper measure for project management profitability?	1.A. EBITDA is an apt measure of Project Management Profitability	0,942	29.077	0,000	significant	0,802	5.716	0,008	Significant
		1.B. EBITDA Project Management is fully under the control of the Project Manager and Team	0,837	9.233	0,001	significant	0,724	0.322	0,285	not significant
**2**	RQ3. How can the Company Maximize EBITDA in the Earned Value Management (EVM) in project management?	2.A. The Company can Maximize EBITDA in EVM by integrating EBITDA to WBS and applying the target EBITDA Margin in WBS and Work Package.	0,875	15.167	0,049	significant	0,767	2.498	0,057	not significant
**3**	RQ4. What are the benefits of using EBITDA as a measure of project management profitability?	3.A. Using EBITDA as a measure of Project Management Profitability allows Project Managers and Teams to actively manage and maximize Project Management Profitability	0,885	16.879	0,020	significant	0,793	4.788	0,014	Significant
		3.B. Applying EBITDA to EVM and WBS enables companies to display and control Project Management Profitability daily	0,875	15.167	0,049	significant	0,758	1.898	0,084	not significant
**4**	RQ5. How can the Company improve its ability to support and provide strategic decisions for Project Management by using Project Management EBITDA?	4.A. The Company can improve its ability to support Project management by sharing daily Targets and Actual results of EBITDA with the Company’s supporting Units	0,904	201.579	0,003	significant	0,802	5.716	0,008	Significant
		4.B. The Company can improve its ability to support Project management by sharing the daily Target and Actual EBITDA with the Board of Directors to provide timely strategic decisions to Project management	0,894	18.683	0,008	significant	0,810	6.726	0,005	Significant

Note: χ2 table = 3.481

The First Batch Hypothesis testing results are shown in Table 4.

1. While testing hypothesis 1.A, both AMC and SBC agree that EBITDA is a suitable measure of Project Management Profitability.

2. In evaluating hypothesis 1.B, there is a divergence of opinions between AMC and SBC regarding the control of EBITDA Project Management, with SBC disagreeing with the statement (*χ*2**- value** < 3.481 and p-value>0.05).

3. When testing hypothesis 2.A, there are differing opinions on whether integrating EBITDA to WBS and applying the target EBITDA Margin in WBS and Work Package can maximize EBITDA in EVM. Less than 70% of SBC respondents disagreed with the statement (*χ*2**- value** < 3.481 and p value> 0.05).

4. Hypothesis 3.A. testing indicates that using EBITDA as a measure of Project Management Profitability allows for active management and maximization of profitability, with approval from most AMC and SBC respondents (more than 70%, with *χ*2**- value** > 3.481 and -value < 0.05).

5. Hypothesis 3.B testing reveals a key insight: applying EBITDA to EVM and WBS empowers companies to display and control Project Management Profitability daily. While SBC respondents may have disagreed (*χ*2**- value** < 3.481 and p-value >0.05), the practical implications of this finding are significant, providing a clear path to enhanced profitability management.

6. In testing hypotheses 4.A and 4.B, a unanimous approval was received from all AMC and SBC respondents. This finding underscores the Company’s potential to enhance its ability to support Project management by sharing daily Targets and Actual EBITDA results with the Company’s supporting Units and the Board of Directors, instilling confidence in the proposed strategies.

#### 4.1.2. Second batch of quantitative data collection and analysis

After implementing Maximizing Daily EBITDA in Aircraft Manufacturing and Shipbuilding Companies for eleven months, we conducted the second round of data collection. We gathered responses from 125 questionnaires without differentiating between aircraft manufacturing and shipbuilding companies.

We tested several EBITDA and operational profitability hypotheses using a test statistic, *χ*2. The results of the testing are summarized below. Supporting Information for the Second Batch of data analysis is available in S5 Appendix.

Our commitment to a comprehensive analysis led us to conduct proportion testing on the data, testing a multitude of hypotheses. This rigorous process is a testament to our dedication to providing the most accurate and insightful findings.

- **Hypothesis 1 C:** EBITDA is an appropriate measure of operational Profitability for implementing company operations and project management.
- **Hypothesis 2. B**: The company can maximize daily EBITDA by integrating EBITDA as a target in business processes and work breakdown structures (WBS) enables company operational and project management to manage Profitability actively.
- **Hypothesis 2. C:** The company can maximize daily EBITDA by integrating EBITDA as a target in Business Processes and Work Breakdown Structure (WBS) that ease Operations Management and Project Management, to increase Profitability.
- **Hypothesis 3.C**: 3. Using EBITDA as a target in business processes and work breakdown structures (WBS) eases operational and project management and allows them to display and control profitability daily.
- **Hypothesis 4 C**: A company can improve its ability to support PM by sharing the daily Target and Actual EBITDA with the supporting unit’s management and Board of Directors to gain support and provide timely strategic decisions to Project management.

H_01_) π ≤ 0.70 and Ha_1_) π > 0.70.

The test statistic used is Chi square test $\chi 2=\frac{{\left(x-E\right)}^{2}}{E}$

$\hat{p}=\frac{x}{n}$, the proportion of people who answered 6 or 7 to the question, and po = 0.70. The decision-making rule was to reject Ho if *χ*2>*χ*2 table with α = 0.05

The results are summarized in Table 5 below.

**Table 5:** Proportion testing result.

Variable	AVERAGE	Proportion	STDEV.	SE (p)	CV
EKPI	6,45	0,912	0,677	0,025	10,51%
EWBM	6,47	0,944	0,603	0,021	9,32%
EWBP	6,42	0,904	0,662	0,026	10,32%
EWBS	6,35	0,872	0,765	0,030	12,04%
EISS	6,48	0,928	0,630	0,023	9,72%

Proportion Testing

Hypothesis

**H**_**01**_: π ≤ 0.70

**Ha**_**1**_: π > 0.70

The test statistics used in Chi square test are $\chi 2=\frac{{\left(x-E\right)}^{2}}{E}$

$\hat{p}=\frac{x}{n}$, x is people who answered 6 or 7 on the question

E = n.po and po = 0.70

**decision making: Reject Ho if** p-value < α (= 0.05)

Sumary sub section 4.1.2:

Table 6 displays the Hypothesis Testing Results

- Regarding the second research question, our proportion test analysis showed that the hypothesis 1. C. statement "EBITDA is an appropriate measure of operational profitability for the implementation of company operations and project management" is significant (χ2 value > χ2 table and p-value = 0.000 < 0.05). This result means that EBITDA is the appropriate measure for project management profitability.
- Regarding the third research question, Hypothesis 2. B states, "The company can integrate EBITDA as a target in Business Processes and Work Breakdown Structure (WBS) to manage profitability actively," and Hypothesis 2. C, "The company can maximize daily EBITDA by Integrating EBITDA as a target in Business Processes and Work Breakdown Structure (WBS) to increase the Profitability of Operations and Project Management" are both significant (χ2 value > χ2 table and p-value = 0.000 < 0.05). These results mean that the company can maximize daily EBITDA project management in the EVM system by integrating EBITDA as a target in Business Processes and Work Breakdown Structure (WBS).
- Regarding the fourth research question, Hypothesis 3. C statement " Using EBITDA as a target in Business Processes and Work Breakdown Structure (WBS) eases Operational Management and Project Management to display and control their profitability every day" is significant (χ2 value > χ2 table and p-value = 0.000 < 0.05). This result means that the company can benefit from using EBITDA to measure project management profitability by integrating EBITDA as a target in Business Processes and Work Breakdown Structure (WBS) that will ease Operational Management and Project Management to display and control their profitability every day.
- Regarding the fifth research question, the hypothesis 4.C: The company can improve its ability to support Project Management by sharing the daily Target dan Actual EBITDA with supporting units’ management and Board of Directors to gain support and provide timely strategic decision to project management" is significant (χ2 value > χ2 table and p-value = 0.000 < 0.05). This result means that the company improves its ability to support and make strategic decisions for project management using EBITDA from the availability of daily EBITDA information for divisions and company operational units, as well as project management, to provide support for achieving successful strategy and project management.

**Table 6:** Second batch hypothesis testing results.

Research Questions	Hypothesis	$\hat{p}$	*χ*2- value	p-value	Conclusion
RQ2. Is EBITDA the proper measure for project management profitability?	1. C EBITDA is an appropriate measure of operational Profitability for the implementation of company operations and project management	0,912	26.752	0,000	significant
RQ3.How can a company maximize daily EBITDA project management in the EVM system?	2.B. The Company can maximize daily EBITDA by integrating EBITDA as a target in Business Processes and Work Breakdown Structure (WBS) to manage Profitability actively.	0,944	35.438	0,000	significant
	2.C. The Company can Maximize daily EBITDA by Integrating EBITDA as a target in Business Processes and Work Breakdown Structure (WBS) to ease Project Management to increase the Profitability	0,904	24.771	0,000	significant
RQ4. What are the benefits of using EBITDA to measure project management profitability?	3.C. Using EBITDA as a target in business processes and work breakdown structures (WBS) eases operational and project management and allows them to display and control profitability daily.	0,872	17.61	0,000	significant
RQ5. How can a company improve its ability to support and make strategic decisions for project management using EBITDA?	4.C The Company can improve its ability to support Project management by sharing the daily Target and Actual EBITDA with the Supporting Unit’s Management and Board of Directors to gain support and provide timely strategic decisions to Project management	0,928	30.943	0,000	significant

Note: if χ2 value > χ2 table (α = 0.05, df = 1, χ2 table = 3.481) then the null hypothesis is rejected (significant)

All proportions who answered 6 and 7 were greater than 0.70 (70%) with a small standard error. Standard Error measures the precision of estimates/estimates of population parameters, reflecting the accuracy of the values calculated from the sample against the population. A small standard error means that the sample mean/mean spread is also small, so the estimate of the population parameter is accurate.

### 4.2. Qualitative data analysis

The qualitative analysis conducted with NVivo showcases the findings from structured interviews. The comprehensive results obtained from NVivo encompasses both structured and unstructured interviews. After completing a comprehensive qualitative data analysis, it has become evident that EBITDA is the most suitable operating profit indicator for the EVM system in Project Management. Integrating EBITDA into EVM involves establishing the budget’s EBITDA value to determine plan costs and applying the EBITDA margin in every work breakdown structure and work package. By using EBITDA to evaluate project management profitability, the company can empower the project manager and team to understand the target EBITDA and maximize profitability. Sharing EBITDA can also enhance support and facilitate timely strategic decisions for Project Management.

The qualitative analysis conducted with NVivo, as depicted in Table 4, showcases the findings from structured interviews. The comprehensive results obtained from NVivo encompass both structured and unstructured interviews.

Discussion of qualitative analysis with NVivo Table 7 and Fig A-3 until A-6 in S1 Appendix display the results of the qualitative analysis using structured interviews by NVivo. Supporting Information of the interview is available in S6 Appendix.

**Table 7:** NVivo qualitative analysis for structured interview.

	1: Strongly disagree– 7 Strongly agree							
	NVivo Qualitative Analysis LIKERT SCALE	1	2	3	4	5	6	7
**RQ1**	**EBITDA is the proper measure of Project Management Profitability**							
1	EBITDA is an appropriate measure of Project Management Profitability because EBITDA is Operational Profitability before payment of Interest, Taxes, Depreciation, and Amortization expenses. The value of EBITDA is not affected by interest, taxes, depreciation, and amortization. EBITDA value is only affected by Project Management Efficiency and Productivity.						3	17
2	EBITDA is the proper measure of Project Management Profitability because EBITDA is entirely under the control of the Project Manager and the Team.						5	15
**RQ2**	**The Company can integrate EBITDA with EVM in project management by determining the budget’s EBITDA value to find the plan costs and applying the EBITDA margin in every work breakdown structure and work package.**							
3	Companies can integrate EBITDA into EVM by establishing a Revenue Plan from the Authorized Budget and an EBITDA Plan to determine the Management Cost Plan.						3	17
4	Companies can integrate EBITDA into EVM by setting a Target EBITDA Margin % in each Work Breakdown Structure and Work Package.						3	17
**RQ3**	**The Company benefits from using EBITDA as a project management profitability measure because the Project Manager and Team can understand the Target EBITDA and maximize Project Management’s Profitability.**							
5	Companies benefit from using EBITDA as a measure of Project Management profitability because EBITDA is a standard operating profitability measure for Financial Statements so that Company Management can understand Project Management Profitability.						4	16
6	Companies benefit from using EBITDA to measure Project Management profitability because it allows Project Managers and Teams to know their EBITDA Targets to maximize Project Management profitability.						4	16
**RQ4**	**The Company can improve its ability to support and provide timely strategic decisions for Project Management by sharing Project Management EBITDA.**							
7	To increase support from the Supporting Unit to Project Management, the Project Manager must report the achievement of Actual EBITDA and Target EBITDA to the Company’s Supporting Unit.						3	17
8	To make it easier for the Board of Directors to provide timely strategic decisions to Project Management, the Project Manager must report the achievement of Actual EBITDA and Target EBITDA to the Board of Directors.						1	19

The complete NVivo results, including unstructured interviews. From this qualitative data analysis, we summarize the following:

### 4.3. Discussion

Referring to the Quantitative and Qualitative data results, we found that some employees needed to be aware of the importance of EBITDA for Project Management during the early assessment.

Regarding H2, less than 70% of the SBC respondents agreed that EBITDA project management is entirely under the control of the project manager and the team. This response is because some operational and project management personnel remained unaware of the EBITDA concept. Many of them were not concerned with controlling EBITDA. The Interview with the chief financial and human capital officer (Interviewee O) confirmed this result.

- *In terms of EBITDA implementation*, *it is the knowledge that is related to human capital*. *So*, *human behavior is my homework (responsibility)*. *All fellows know that EBITDA is important and is under control*. *However*, *this knowledge becomes essential when you remind them*. *They already know the definition of direct costs and indirect costs and responsibilities*. *However*, *when you explain how to maximize daily EBITDA*, *they realize it is an integrated system under their control*. *So they feel more responsible*. *To make this system work*, *I have homework in human capital because this concerns organizational behavior; that is what I see*.

Regarding H3, less than 70% of SBC respondents agreed that a company can maximize EBITDA in EVM by integrating EBITDA into WBS and applying the target EBITDA margin in WBS and the Work Package. This response is because some people in the SBC did not have experience implementing the EBITDA–EVM integration. The SBC was in the early stage of implementing EBITDA–EVM integration using its information technology system, the ITS. The senior vice president of corporate transformation (Interviewee D) confirmed this explanation in the Interview.

- *We can trace every Project Management indicator to this EBITDA implementation system and the work breakdown structure*. *Ultimately*, *it will be an institutional business in the direction of profit and lose continuity from the sustainability [achieved] earlier*. *Controlling the daily EBITDA of the EVM with these Information Technology System (ITS) tools is an extraordinary combination for managing an enterprise*. *Moreover*, *we know that the project’s development is a one-time event*. *There cannot be any delays*. *We must synergize all resources and balance quality*, *cost*, *and delivery time*. *With these tools*, *we get help to control the project management*. *That is my opinion*.

Regarding H5, less than 70% of the SBC respondents agreed that applying EBITDA to EVM and WBS enables companies to display and control project management profitability daily. This response is because some SBC employees need experience managing daily Profitability. However, the management expected the situation to change because the Company has begun implementing daily control and maximizing daily EBITDA in the EVM system, supported by their information system, ITS. The vice president of human capital (Interviewee M) confirmed this explanation and explained the Company’s policy to control employees’ daily activities using handphone devices provided by the Company.

- *Integrating the daily EBITDA-maximizing program into the EVM is especially important now that the Company has issued a mandate to integrate the program through the IT system using handphone devices*. *The Company has distributed 1*,*500 handphone devices to employees to control and report on their activities and working hours*. *They must fill in the timesheet via handphone to record their daily attendance and activities*. *Of course*, *every report must be supervised and validated by their respective superiors*.

After eleven months of maximizing daily EBITDA in the EVM system project management, we found significant improvement in understanding the concept from the survey of both companies.

Regarding the first research question, we found that the respondents agree that EBITDA is appropriate for project management profitability. Fig 2 displays the integration of EVM into the Income Statement using seven earnings steps. Fig 3 shows EBITDA under the control of Operation Management and Project Management. On the other hand, Depreciation, Amortization, Interest, and Tax are not under the control of Operation Management but of the Board of Directors.

Regarding the second research question, we found that respondents agree that the Company can maximize daily EBITDA project management in the EVM system by Integrating EBITDA as a target in Business Processes and Work Breakdown Structure (WBS).

Regarding the third Research Question, we found that the respondents agree that the Company can get the benefits of using EBITDA to measure project management profitability from Integrating EBITDA as a target in Business Processes and Work Breakdown Structure (WBS) that will ease Operational Management and Project Management to display their Profitability every day.

Regarding the fourth research question, we found that respondents agree that the Company improved its ability to support and make strategic decisions for project management using EBITDA from the availability of daily EBITDA information for divisions and company operational units, as well as project management to provide support for achieving successful strategy and project management.

All proportions who answered 6 and 7 were more significant than 0.70 (70%) with a relatively small standard error. Standard Error measures the precision of estimates/estimates of population parameters, reflecting the accuracy of the values ​​calculated from the sample against the population. A small standard error means that the sample mean/mean spread is also small, so the estimate of the population parameter is accurate.

Based on our assessment, some employees need to understand the significance of EBITDA for project management. Less than 70% of the SBC respondents initially agreed to incorporate EBITDA into WBS and apply the target EBITDA margin in WBS and the Work Package to optimize EVM. The SBC is still in the early stages of integrating EBITDA and EVM using its information technology system, the ITS. Significant improvements were observed after nearly a year of focusing on integrating EVM into income statements and maximizing daily EBITDA in the EVM system project management. The respondents concur that EBITDA is the appropriate measure for project management profitability. The consensus among respondents was that the company could enhance daily EBITDA project management in the EVM system by integrating EBITDA as a target in business processes and work breakdown structures (WBS). Concerning management practices, when proposing a program like integrating EVM into an income statement in project management, managers consider the expected costs of adoption against the expected benefits. The new system’s speed and ease of adoption will depend on corporate and national cultures [86].

It is strategically advantageous to incorporate EBITDA into every Work Breakdown Structure (WBS) and Work Package. This integration allows the management team to effectively oversee job assignments. When combined with Earned Value Management (EVM), this approach can significantly enhance profitability by improving efficiency and productivity.

The integration of EVM into income statements and EBITDA into WBS aligns project management and financial systems. This common language enables leaders to cultivate a robust corporate culture, enhance efficiency, and improve productivity for superior performance and profitability. By comparing EBITDA, EBITDA Margin, and their respective trends before and during project management execution, organizations can make informed decisions about resource allocation priorities in each WBS Project portfolio.

Despite the importance and widespread nature of projects, there is currently little space devoted to project management research in leading business and management journals. We need more research for systemic approaches to projects, situating them in broader organizational landscapes. Addressing grand challenges involves changing project management practices to create and distribute value among diverse stakeholders [3].

The principles of Earned Value Management (EVM) and Project Management theory are accessible to all project management professionals and can be applied across various business and management domains. It is hoped that professionals in fields other than project management will adopt these concepts.

## 5. Conclusions

This study demonstrates that incorporating EVM into income statements can enhance project management profitability, reduce information asymmetry, and garner support from stakeholders for making strategic decisions to ensure project management success. Specifically, this integration allows project managers to optimize Daily EBITDA within each Work Breakdown Structure (WBS) and achieve improved performance and profitability. Integrating EVM into the income statement was challenging and required a shift in organizational culture. However, after eleven months of focusing on maximizing daily EBITDA within the EVM system, there was a notable improvement in understanding the concept based on the survey responses from participating companies. The study also highlights that EBITDA is a suitable metric for assessing project management profitability, as it is within the control of Operations Management and Project Management. Conversely, factors such as depreciation, amortization, interest, and taxes fall under the purview of the board of directors rather than operations management.

Furthermore, by incorporating EBITDA as a target within each WBS, companies can effectively maximize daily EBITDA in project management within the EVM system, providing a clear view of profitability daily. This integration will facilitate communication between project management professionals and business management and foster greater acceptance of project management concepts within the business and management fields. Hopefully, this will also encourage dialogue between project management scholars and those in related disciplines, particularly business and management.

The integration of EVM into income statements and EBITDA into WBS not only combines the project management and financial systems but also integrates the language of both fields, enabling leaders to foster a strong corporate culture, improve efficiency, and enhance productivity for better performance and profitability.

The Organization can improve its decision-making regarding resource allocation priorities in each WBS Project portfolio by conducting a thorough comparison of EBITDA, EBITDA Margin, and their respective trends both before and during project management execution.

This study provides valuable insights and evidence-based ideas about project management and can potentially enhance its use in the business and management sectors. The author’s unique contribution lies in integrating EVM into the income statement with EBITDA into WBS. This research ensures we can apply the theoretical concepts effectively in practical settings with broader implications.

The implications of Earned Value Management (EVM) and Project Management theory are understandable to any project management professional and can be applied across various business and management sectors. It is hoped that professionals in fields beyond project management will embrace these concepts. These topics warrant further in-depth exploration in future research.

## Supporting information

S1 Appendix EVM-income statement rev 241021. (DOCX)

S2 Appendix Case study protocol EVM 241021. (DOCX)

S3 Appendix COREQ checklist EVM PM 241021. (DOCX)

S4 Appendix #1 Batch EVM PM 241021. (XLSX)

S5 Appendix #2 Batch 125 questionnaire EVM PM. (XLSX)

S6 Appendix Binder interview EVM–Rev 241021. (DOCX)
