# Supplementary material for: Incorporating earned value management into income statements to improve project management profitability and elevate application in the business and management
Source: PLoS One. 2025 Jan 3;20(1):e0312956. doi: 10.1371/journal.pone.0312956 (PMC11698386; doi:10.1371/journal.pone.0312956)
Supplement: S1 Appendix — (DOCX) [file pone.0312956.s001.docx]

**S1 APPENDIX -1 SUPPORTING INFORMATION**

**Incorporating Earned Value Management into Income Statements**

**to improve Project Management Performance and Profitability**

**and Elevate Application in the Business and Management**

Figure A-1. Tabular matrix of shortlisted EVM literature and the word frequency of Earned, Earnings, Profit, and Revenue.

Figure A-2. Tabular matrix of shortlisted EVM literature and the word frequency of Earned, Earnings, Profit, and Revenue.

Discussion of qualitative analysis with NVivo Table 4 (in the manuscript) and Figure A-3  until A-6 display the results of the qualitative analysis using structured interviews by NVivo. The complete NVivo results, including unstructured interviews. From this qualitative data analysis, we summarize the following:

- EBITDA is the proper operating profit indicator for the EVM system for Project Management.


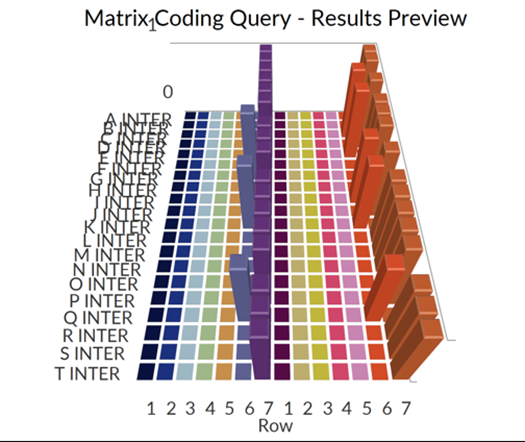


Figure A-3 EBITDA is a proper measure of Project Management Profitability, NVivo result.

- The Company can integrate EBITDA into EVM in project management by determining the budget's EBITDA value to determine the plan costs and applying the EBITDA margin in every work breakdown structure and work package.


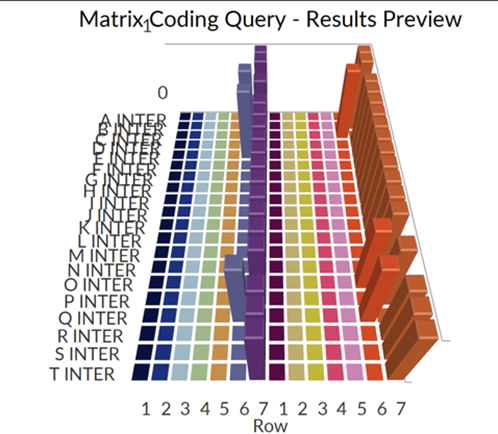


Figure A-4. EBITDA – EVM integration: Authorized Budget = Revenue, NVivo result.

- The Company benefits from using EBITDA as a measure of project management profitability because the project manager and team can understand the target EBITDA and maximize its Profitability.


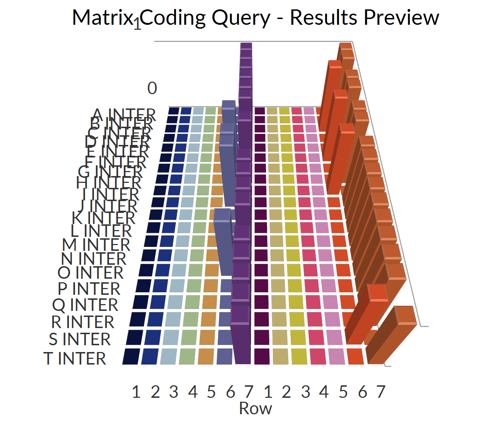


Figure A-5. EBITDA Project Management Benefit: Stakeholders and PM Profitability

- The Company can improve its support and provide timely strategic decisions for Project management by sharing EBITDA.


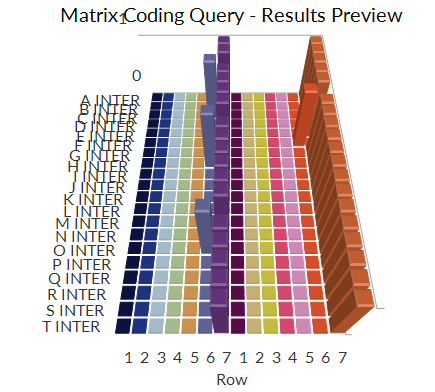


**Figure A-6. EBITDA Project Management Benefit: Stakeholders and PM Profitability**


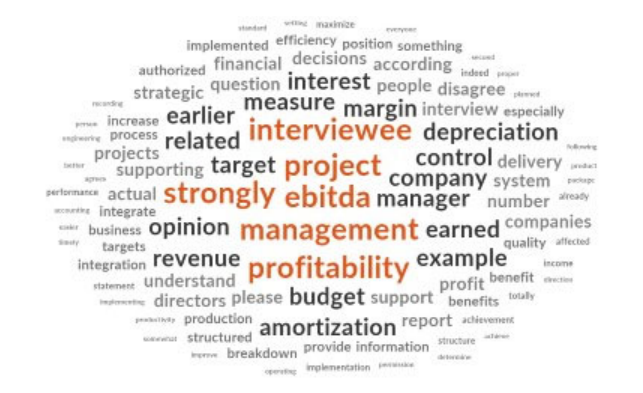


**Figure A-7 Word Cloud, minimum length = 6, most frequent 1000 words**


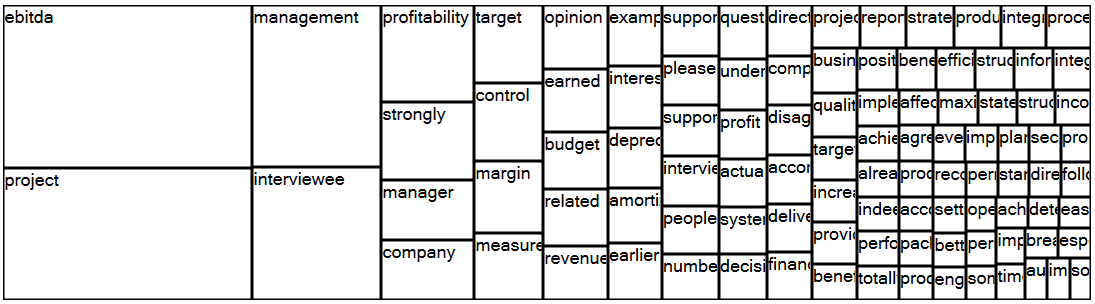


**Figure A-8. Word Tree Map, minimum length = 6, most frequent 1000 words**

**Table A-1 Codes. NVivo Result**

EBITDA-EVM Project Management

Codes

| Subjects | Description | Files | References |
| --- | --- | --- | --- |
| 1. EBITDA is a proper measure of PM profitability | EBITDA is the proper measure of Project Management Profitability | 20 | 41 |
| 1 EBITDA is a measure of efficiency and productivity | EBITDA is an appropriate measure of Project Management Profitability because EBITDA is Operational Profitability before payment of Interest, Taxes, Depreciation, and Amortization expenses. The value of EBITDA is not affected by interest, taxes, depreciation, and amortization. EBITDA value is only affected by Project Management Efficiency and Productivity | 20 | 20 |
| 1 | Strongly Disagree that EBITDA is Efficiency & Productivity | 0 | 0 |
| 2 | Somewhat disagree that EBITDA is Efficiency & Productivity | 0 | 0 |
| 3 | Disagree EBITDA is Efficiency & Productivity | 0 | 0 |
| 4 | Neutral EBITDA is Efficiency & Productivity | 0 | 0 |
| 5 | Somewhat agree that EBITDA is Efficiency & Productivity | 0 | 0 |
| 6 | Agree EBITDA is Efficiency & Productivity | 3 | 3 |
| 7 | Strongly agree that EBITDA is Efficiency & Productivity | 17 | 17 |
| 2 EBITDA controlled by PM &Team | EBITDA is the proper measure of Project Management Profitability because EBITDA is entirely under the control of the Project Manager and the Team. | 20 | 20 |
| 1 | Strongly disagree that EBITDA is Efficiency & Productivity | 0 | 0 |
| 2 | Disagree EBITDA is Efficiency & Productivity | 0 | 0 |
| 3 | Somewhat disagree that EBITDA is controlled by Project Manager and Teams | 0 | 0 |
| 4 | Neutral EBITDA is controlled by the Project Manager and Teams | 0 | 0 |
| 5 | Somewhat agree that EBITDA is controlled by the Project Manager and Teams | 0 | 0 |
| 6 | Agree the Project Manager and Teams control EBITDA | 5 | 5 |
| 7 | Strongly agree that EBITDA is controlled by the Project Manager and Teams | 15 | 15 |
| 2. EBITDA - EVM integration | The Company can integrate EBITDA with EVM in project management by determining the EBITDA value to the budget to find the plan costs and apply the EBITDA margin in every work breakdown structure and work package. | 20 | 36 |
| 1 EBITDA - EVM Budget Revenue | Companies can integrate EBITDA into EVM by establishing a Revenue Plan from the Authorized Budget and an EBITDA Plan to determine the Management Cost Plan. | 20 | 22 |
| 1 | Strongly disagree EBITDA can be integrated with EVM by determining Authorized Budget as Planned Revenue of Project Contractor, Determining Planned EBITDA, and Planned Costs = Planned Revenue - Planned EBITDA | 0 | 0 |
| 2 | Disagree EBITDA can be integrated with EVM by determining Authorized Budget as Planned Revenue of Project Contractor, Determining Planned EBITDA, and Planned Costs = Planned Revenue - Planned EBITDA | 0 | 0 |
| 3 | Somewhat disagree EBITDA can be integrated with EVM by determining Authorized Budget as Planned Revenue of Project Contractor, Determining Planned EBITDA, and Planned Costs = Planned Revenue - Planned EBITDA | 0 | 0 |
| 4 | Neutral EBITDA can be integrated with EVM by determining Authorized Budget as Planned Revenue of Project Contractor, Determining Planned EBITDA, and Planned Costs = Planned Revenue - Planned EBITDA | 0 | 0 |
| 5 | Somewhat agree EBITDA can be integrated with EVM by determining Authorized Budget as Planned Revenue of Project Contractor, Determining Planned EBITDA, and Planned Costs = Planned Revenue - Planned EBITDA | 0 | 0 |
| 6 | Agree EBITDA can be integrated with EVM by determining Authorized Budget as Planned Revenue of Project Contractor, Determining Planned EBITDA, and Planned Costs = Planned Revenue - Planned EBITDA | 3 | 3 |
| 7 | Strongly agree that EBITDA can be integrated with EVM by determining Authorized Budget as Planned Revenue of the Project Contractor, Determining Planned EBITDA, and Planned Costs = Planned Revenue - Planned EBITDA | 17 | 17 |
| 2 EBITDA - EVM WBS Margin | Companies can integrate EBITDA into EVM by setting a Target EBITDA Margin % in each Work Breakdown Structure and Work Package. | 20 | 20 |
| 1 | Strongly disagree that EBITDA can be integrated with EVM by determining the EBITDA Margin of Project Management to WBS and Work Packages, Top-Down and Bottom-Up | 0 | 0 |
| 2 | Disagree EBITDA can be integrated with EVM by determining the EBITDA Margin of Project Management to WBS and Work Packages, Top-Down, and Bottom-Up | 0 | 0 |
| 3 | Somewhat disagree EBITDA can be integrated with EVM by determining the EBITDA Margin of Project Management to WBS and Work Packages, Top-Down, and Bottom-Up | 0 | 0 |
| 4 | Neutral EBITDA can be integrated with EVM by determining the EBITDA Margin of Project Management to WBS and Work Packages, Top-Down, and Bottom-Up | 0 | 0 |
| 5 | Somewhat agree that EBITDA can be integrated with EVM by determining the EBITDA Margin of Project Management to WBS and Work Packages, Top-Down and Bottom-Up | 0 | 0 |
| 6 | Agree EBITDA can be integrated with EVM by determining the EBITDA Margin of Project Management to WBS and Work Packages, Top-Down, and Bottom-Up | 3 | 3 |
| 7 | Strongly agree that EBITDA can be integrated with EVM by determining the EBITDA Margin of Project Management to WBS and Work Packages, Top-Down, and Bottom-Up | 17 | 17 |
| 3. Benefit EBITDA | The Company benefits from using EBITDA as a project management profitability measure because the Project Manager and Team can understand the Target EBITDA and maximize Project Management's Profitability. | 20 | 35 |
| 1 Benefit Standard Financial Report | Companies benefit from using EBITDA as a measure of Project Management profitability because EBITDA is a standard operating profitability measure for Financial Statements so that Company Management can understand Project Management Profitability. | 20 | 21 |
| 1 | Strongly disagree. The Company and stakeholders benefit from EBITDA-EVM integration that enables them to know project management profitability at any time of the event. | 0 | 0 |
| 2 | Disagree: The Company and stakeholders benefit from EBITDA-EVM integration that enables them to know project management profitability at any time of the event. | 0 | 0 |
| 3 | Somewhat disagree. The Company and stakeholders benefit from EBITDA-EVM integration that enables them to know project management profitability at any time of the event. | 0 | 0 |
| 4 | Neutral The Company and stakeholders benefit from EBITDA-EVM integration that enables them to know project management profitability at any time of the event. | 0 | 0 |
| 5 | Somewhat agree. The Company and stakeholders benefit from EBITDA-EVM integration that enables them to know project management profitability at any time of the event. | 0 | 0 |
| 6 | Agree: The Company and stakeholders benefit from EBITDA-EVM integration that enables them to know project management profitability at any time of the event. | 4 | 4 |
| 7 | Strongly agree. The Company and stakeholders benefit from EBITDA-EVM integration that enables them to know project management profitability at any time of the event. | 16 | 16 |
| 2 Benefit PM Maximize EBITDA | Companies benefit from using EBITDA to measure Project Management profitability because it allows Project Managers and Teams to know their EBITDA Targets to maximize Project Management profitability. | 20 | 20 |
| 1 | Strongly disagree The Project Management benefits from EBITDA-EVM integration that enables Project Managers and Teams to maximize EBITDA under constraints of Planned Quality, Delivery, and Safety. | 0 | 0 |
| 2 | Disagree: The Project Management benefits from EBITDA-EVM integration that enables the Project Manager and Teams to maximize EBITDA under constraints of Planned Quality, Delivery, and Safety. | 0 | 0 |
| 3 | Somewhat disagree. The Project Management benefits from EBITDA-EVM integration that enables Project Managers and Teams to maximize EBITDA under constraints of Planned Quality, Delivery, and Safety. | 0 | 0 |
| 4 | Neutral The Project Management benefits from EBITDA-EVM integration that enables the Project Manager and Teams to maximize EBITDA under constraints of Planned Quality, Delivery, and Safety. | 0 | 0 |
| 5 | Somewhat agree That Project Management benefits from EBITDA-EVM integration that enables Project Managers and Teams to maximize EBITDA under constraints of Planned Quality, Delivery, and Safety. | 0 | 0 |
| 6 | Agree The Project Management benefits from EBITDA-EVM integration that enables the Project Manager and Teams to maximize EBITDA under constraints of Planned Quality, Delivery, and Safety. | 4 | 4 |
| 7 | Strongly agree That Project Management benefits from EBITDA-EVM integration that enables Project Managers and Teams to maximize EBITDA under constraints of Planned Quality, Delivery, and Safety. | 16 | 16 |
| 4. Share EBITDA to get support | The Company can improve its ability to support and provide timely strategic decisions for Project Management by sharing Project Management EBITDA. | 20 | 38 |
| 1 Share to Supporting Units | To increase support from the Supporting Unit to Project Management, the Project Manager must report the achievement of Actual EBITDA and Target EBITDA to the Company's Supporting Unit. | 20 | 21 |
| 1 | Strongly disagree. Sharing the Project Management’s EBITDA Target and Achievement with the company management enables the management to provide better support to the Project Management to achieve better performance and Profitability. | 0 | 0 |
| 2 | Disagree: Sharing the Project Management’s EBITDA Target and Achievement with the company management enables the management to provide better support to the Project Management to achieve better performance and Profitability. | 0 | 0 |
| 3 | Somewhat disagree; sharing the Project Management’s EBITDA Target and Achievement with the company management enables the management to provide better support to the Project Management to achieve better performance and Profitability. | 0 | 0 |
| 4 | Neutral Sharing the Project Management’s EBITDA Target and Achievement to the company management enables the management to provide better support to the Project Management to achieve better performance and Profitability. | 0 | 0 |
| 5 | Somewhat agree that Sharing the Project Management’s EBITDA Target and Achievement to the company management enables the management to provide better support to the Project Management to achieve better performance and Profitability. | 0 | 0 |
| 6 | Agree that sharing the Project Management’s EBITDA Target and Achievement with the company management enables the management to provide better support to the Project Management to achieve better performance and Profitability. | 3 | 3 |
| 7 | Strongly agree that Sharing the Project Management’s EBITDA Target and Achievement with the company management enables the management to provide better support to the Project Management to achieve better performance and Profitability. | 17 | 17 |
| 2 Share to BOD | To make it easier for the Board of Directors to provide timely strategic decisions to Project Management, the Project Manager must report the achievement of Actual EBITDA and Target EBITDA to the Board of Directors. | 20 | 20 |
| 1 | Strongly disagree. Sharing the Project Management’s EBITDA Target and Achievement with the Company Board of Directors (BOD) enables the BOD to make timely direction and strategic decisions beyond the Project Manager’s responsibility to achieve better project management performance and Profitability. | 0 | 0 |
| 2 | Disagree. Sharing the Project Management’s EBITDA Target and Achievement with the Company Board of Directors (BOD) enables the BOD to make timely direction and strategic decisions beyond the Project Manager’s responsibility to achieve better project management performance and Profitability. | 0 | 0 |
| 3 | Somewhat disagree. Sharing the Project Management’s EBITDA Target and Achievement with the Company Board of Directors (BOD) enables the BOD to make timely direction and strategic decisions beyond the Project Manager’s responsibility to achieve better project management performance and Profitability. | 0 | 0 |
| 4 | Neutrally sharing the project management’s EBITDA target and achievement with the company board of directors (BOD) enables the BOD to make timely direction and strategic decisions beyond the project manager’s responsibility to achieve better project management performance and Profitability. | 0 | 0 |
| 5 | Somewhat agree. Sharing the Project Management’s EBITDA Target and Achievement with the Company Board of Directors (BOD) enables the BOD to make timely direction and strategic decisions beyond the Project Manager’s responsibility to achieve better project management performance and Profitability. | 0 | 0 |
| 6 | Agree that sharing the project management’s EBITDA target and achievement with the company board of directors (BOD) enables the BOD to make timely direction and strategic decisions beyond the project manager’s responsibility to achieve better project management performance and Profitability. | 1 | 1 |
| 7 | I strongly agree that sharing the project management’s EBITDA target and achievement with the company board of directors (BOD) enables the BOD to make timely direction and strategic decisions beyond the project manager’s responsibility to achieve better project management performance and Profitability. | 19 | 19 |

Table A-1 shows the results from N-vivo Analysis.

**Supplementary Material**

**Standard Operation Procedure for Maximizing Daily EBITDA System**

**in EVM Project Management.**

1. For the long-term Project Planning, conduct the following steps in Figure A-9:
   1. Translate the project plan into WBS and Work Package
   2. Determine Project Management Authorized Budget, Plan Revenue, Plan Cost, Plan EBITDA, and Plan EBITDA Margin.
   3. Distribute Plan revenue, Plan Costs, Plan EBITDA, and Plan EBITDA Margin to all levels of WBS and Work Package (Top-Down)
   4. Verify the Plan revenue, Plan Costs, Plan EBITDA, and Plan EBITDA Margin from the lowest level of WBS and Work Package (Bottom-Up)
   5. Use the EBITDA Matrix to find the optimum efficiency and productivity to maximize EBITDA.
   6. Use the Capacity Matrix to prepare Capacity requirements and find bottlenecks and overcapacity.
   7. Solve the bottleneck and over-capacity problem to maximize Project Management EBITDA.


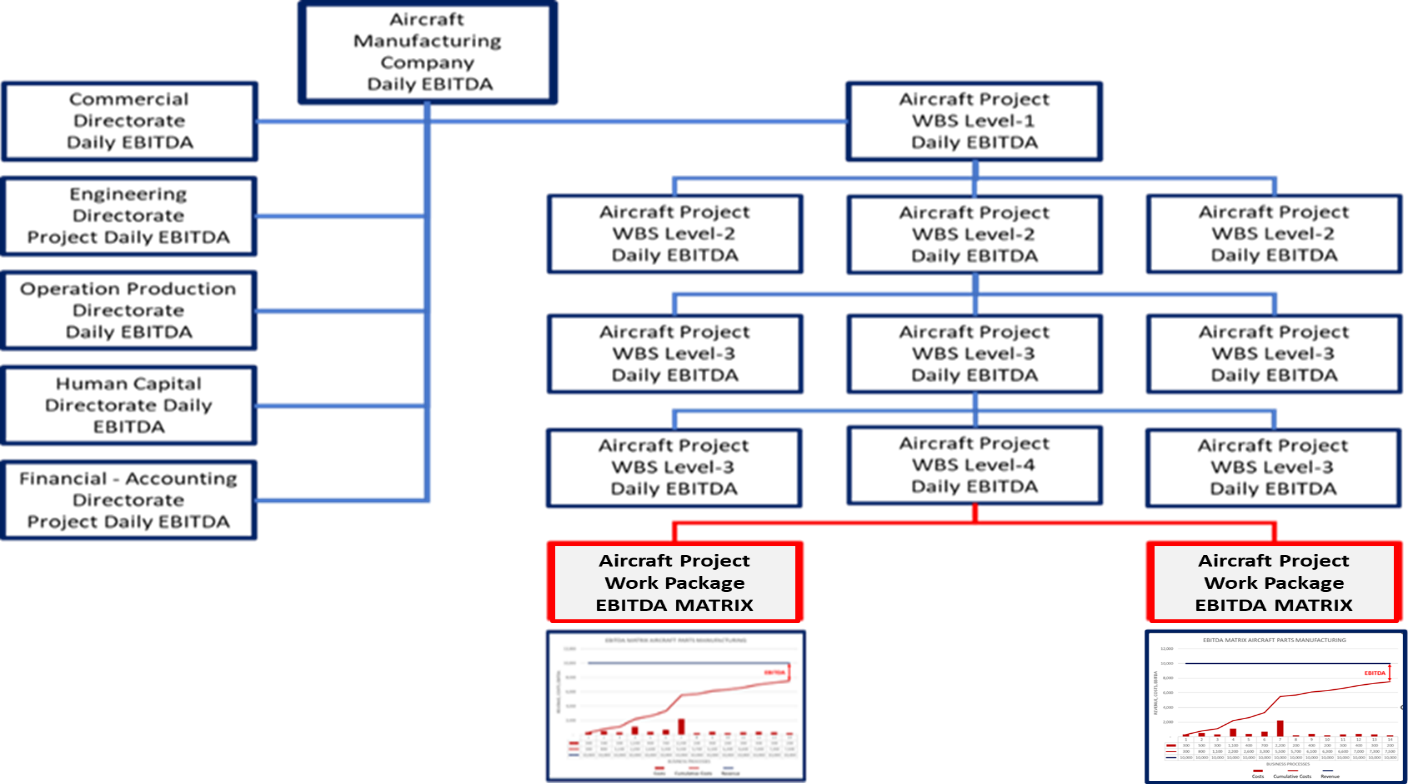


Figure A-9. Distribution of EBITDA and EBITDA Margin to every WBS and Work Package

1. For daily project management to maximize Daily EBITDA, take the following steps.
   1. Translate WBS and Work Package into Daily EBITDA Matrix and daily Capacity Matrix.
      1. Conduct external environmental scans to identify opportunities and constraints and internal environmental scans to identify resource availability and constraints.
   2. Conduct simulation to maximize Daily EBITDA and produce the best operation / Project daily plan scenario.
   3. Prepare all resources, equipment, tools, human resources, materials, documents, and information for Project management execution.
   4. Execute Project Management
   5. Conduct Daily Briefing and Feedback Control of Daily EBITDA Targets, Results, and gaps.
   6. *Follow up with regular EBITDA Control meetings, weekly and monthly.*
   7. *Follow up with a continuous improvement program.*

**
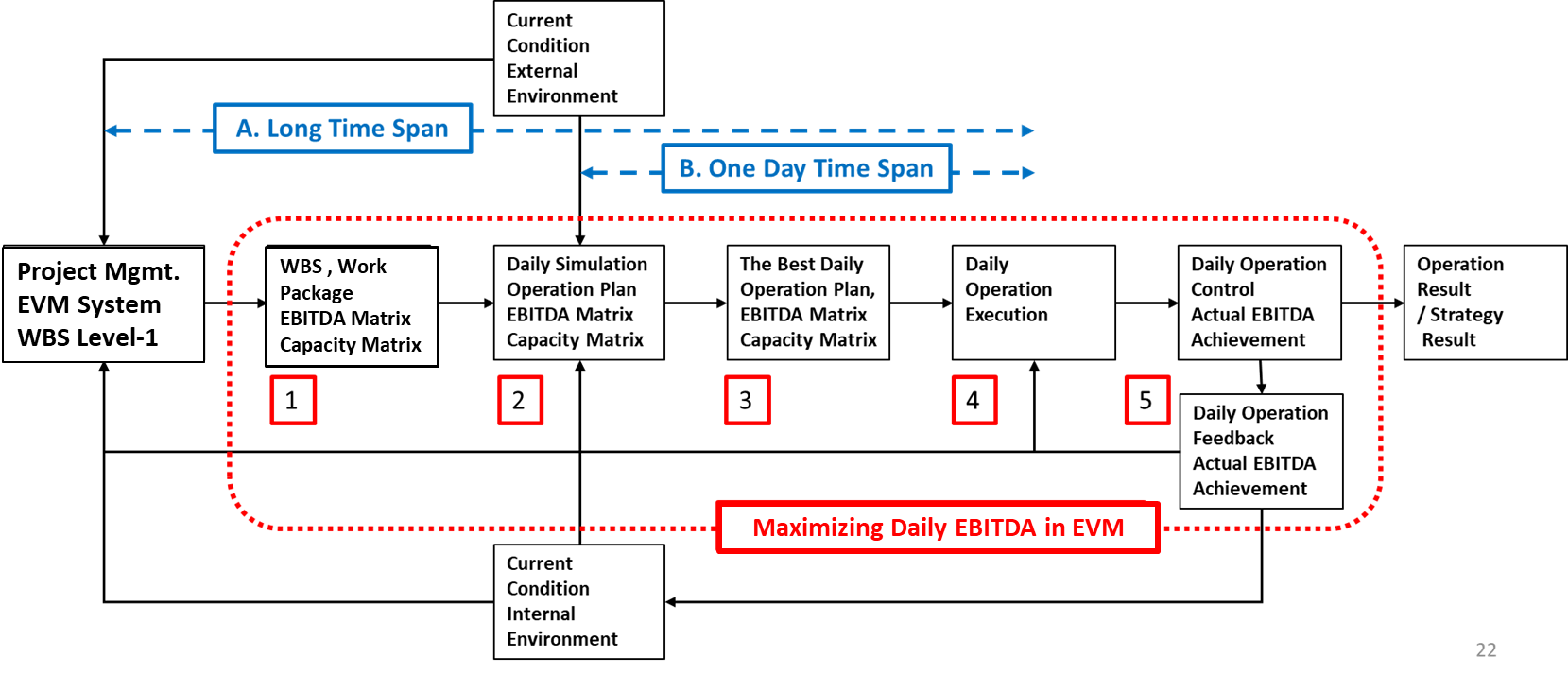
**

Figure A-10. Maximizing Daily EBITDA Simulation in every WBS and Work Package
