# Supplementary material for: Incorporating earned value management into income statements to improve project management profitability and elevate application in the business and management
Source: PLoS One. 2025 Jan 3;20(1):e0312956. doi: 10.1371/journal.pone.0312956 (PMC11698386; doi:10.1371/journal.pone.0312956)
Supplement: S2 Appendix — (DOCX) [file pone.0312956.s002.docx]

S2 APPENDIX 2 SUPPORTING INFORMATION

CASE STUDY PROTOCOL

Incorporating Earned Value Management into Income Statements to improve Project Management Profitability

and Elevate Application in the Business and Management

| **Section A. Overview of the Case Study** | | |
| --- | --- | --- |
| 1 |  | Mission & Goals: to provide practical management to incorporate earned value management into income statements to improve project management profitability and elevate applications in business and management. |
| 2 | A | Case Study Questions:  1.  RQ1. How can a company enhance its EVM application beyond PM to General Business Management?  2.  RQ2. Is EBITDA the appropriate measure for assessing PM profitability?  3.  RQ3. How can a company maximize daily EBITDA in the EVM approach to PM?  4.  RQ4. What are the benefits of using EBITDA to measure PM profitability?  5.  RQ5. How can a company enhance its ability to support and make strategic PM decisions using EBITDA? |
|  | B | Hypothesis 1: EBITDA is a suitable measure of project management profitability because it excludes interest, tax, depreciation, and amortization, which do not fluctuate according to variations in project management efficiency and productivity.  Hypothesis 2: EBITDA is a suitable measure of project management profitability because it is entirely within the control of the project manager and the team.  Hypothesis A: Integrating EBITDA as a target in business processes and WBS enables project managers to manage profitability actively.  Hypothesis B: Integrating EBITDA as a target in business processes and WBS allows project managers to increase profitability.  Hypothesis 3: The Company can maximize EBITDA with EVM in PM by integrating EBITDA into WBS and applying the target EBITDA margin to every WBS and work package.  Hypothesis C: Integrating EBITDA as a target in business processes and work breakdown structures simplifies the project manager's ability to demonstrate daily profitability.  Hypothesis D: The availability of daily EBITDA information for divisions, company operational units, and PM is essential to support the project manager in achieving successful strategy and PM targets.  Hypothesis 4: Using EBITDA to measure PM profitability allows project managers and teams to actively manage and maximize PM profitability.  Hypothesis 5: Applying EBITDA to EVM and WBS enables companies to display and control daily PM profitability.  Hypothesis 6: A company can improve its ability to support PM by sharing the daily target and actual EBITDA result with its supporting units.  Hypothesis 7: A company can improve its ability to support PM by sharing the daily target and actual EBITDA with the board of directors to provide timely strategic decisions to the project manager. |
|  |  |  |
|  |  | **Figure 1. The seven layers of the structure of an income statement, EVM, and its relationship with management control. (Author’s property)** |
|  |  | 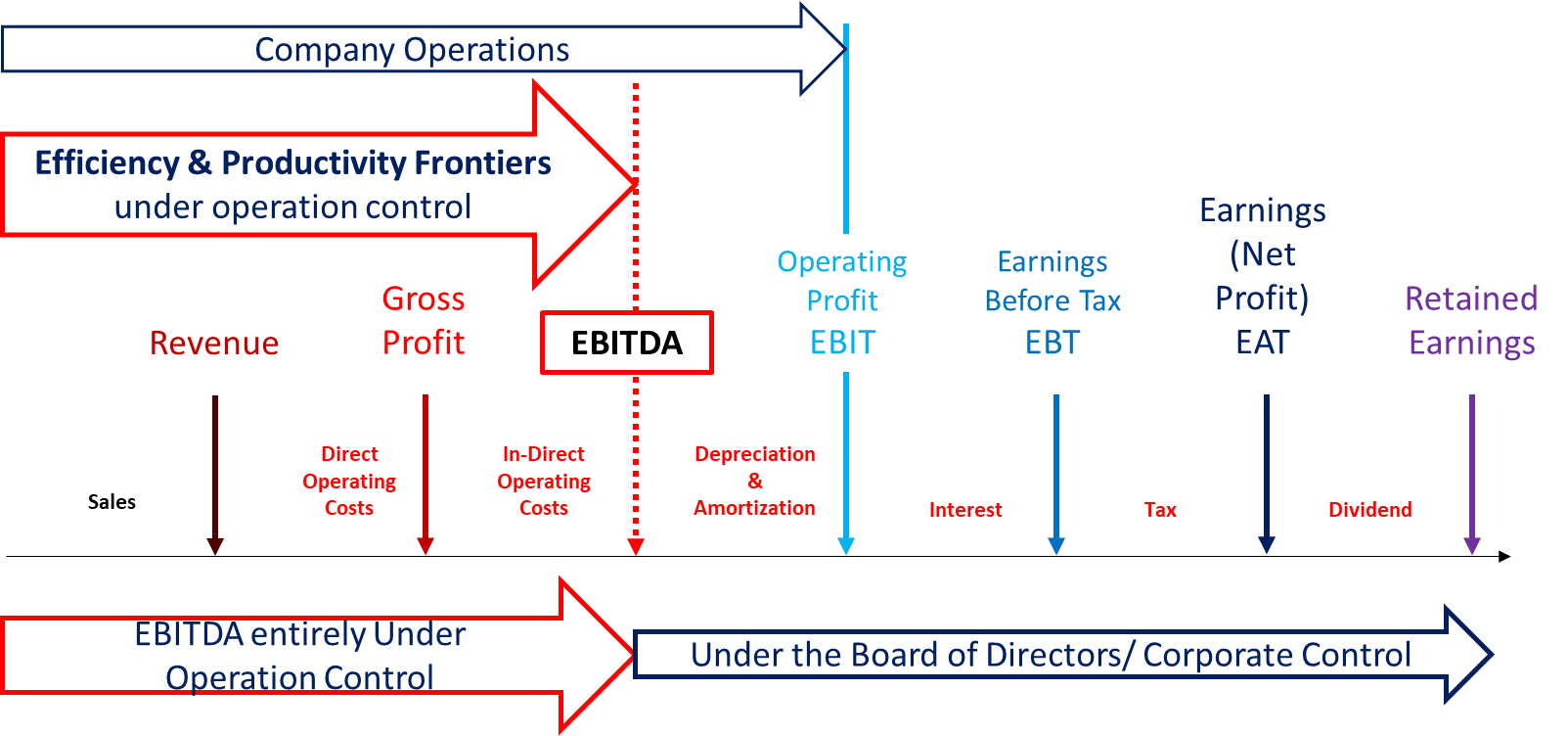 |
|  |  | **Figure 2. Company Earnings and their relationship with the Management Authority and Control (Author’s property)** |
|  |  | 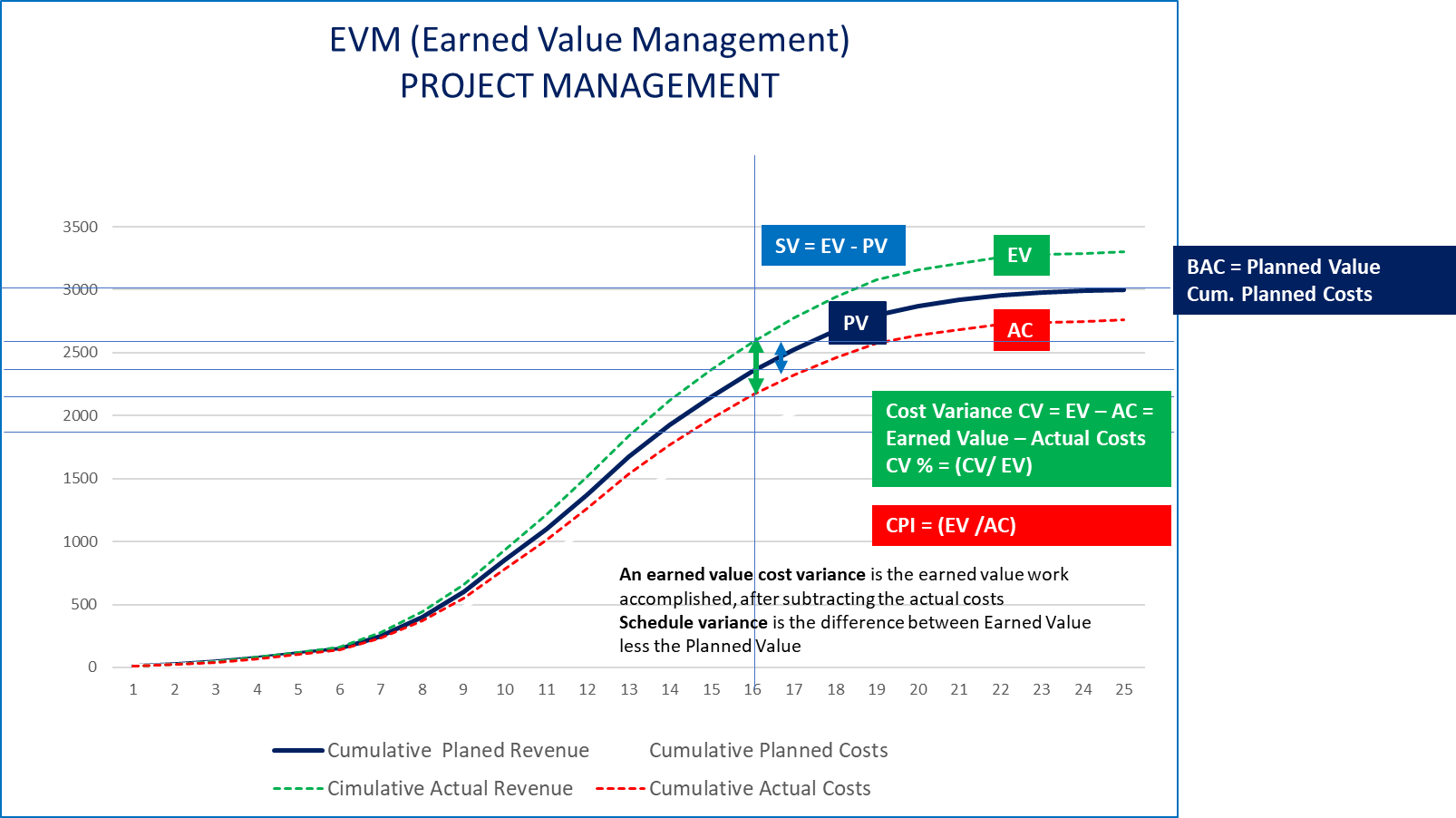 |
|  |  | **Figure 3. S-Curve EVM project management from a project owner’s perspective (Author’s property)** |
|  |  | 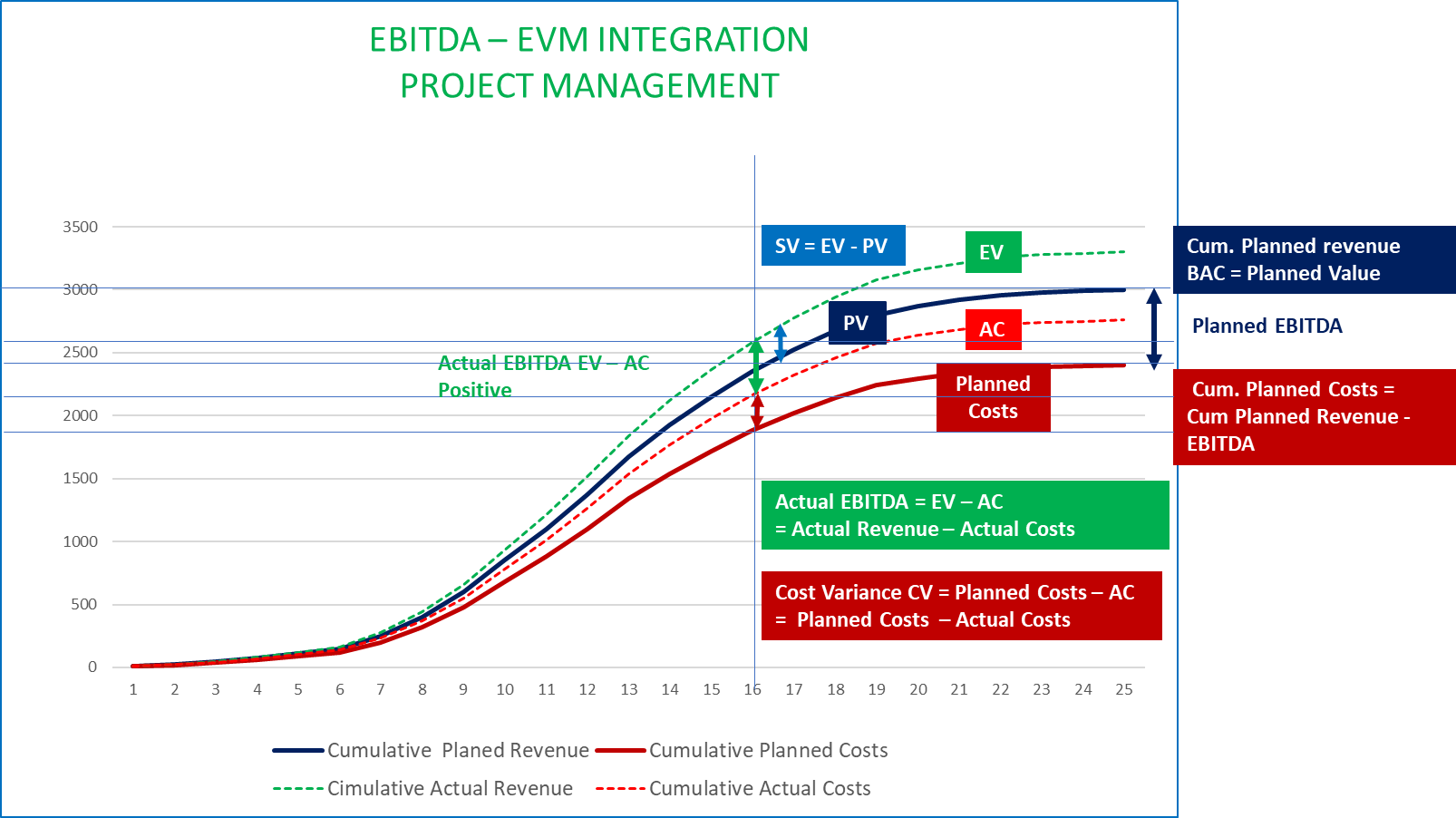 |
|  |  | **Figure 4. S- Curve EBITDA–EVM Integration in project management with a positive actual EBITDA from the project manager’s perspective (Author’s property)** |

| 3 |  | The theoretical framework for the case study includes the following:  1. Project-Based Organization  2. Agency Theory  3. Theory of Production Frontiers  4. Best Practices  5. Project Management  6. Earned Value Management  7. Financial Management  8. Business Process Management  9. Time-Driven Activity-Based Costing  10. Operation Management |
| --- | --- | --- |

| 4 |  | Role of Protocol in guiding the case study researcher ( Protocol serves as the agenda for the researchers' line of inquiry) |
| --- | --- | --- |
| **Section B. Data Collection Procedures.** | | |
| 1 |  | List of Contact Persons for Fieldwork:  1. CFO (Chief Financial Officer)  2. COO (Chief Operating Officer)  3. EVP Transformation  4. EVP Technology & Naval Systems  5. VP Finance  6. VP Accounting  7. VP Human Resources  8. VP Engineering  9. VP Information Technology  10. VP Production  11. Project Management Team |
| 2 |  | Data Collection Plan:  1. Types of Evidence to be Expected:  a. Interview Records  b. Questionnaire Results  c. Photographs  d. Minutes of Meetings  e. EBITDA Reports  2. Roles of People to be Interviewed:  a. President Director/CEO  b. Board of Directors  c. Vice Presidents  d. General Managers  e. Managers  f. Assistant Managers  g. PIC of Operation Control Center/Production Planning & Control  3. Events to be Observed:  a. Training Workshops  b. EVM Integration to Income Statement  c. EBITDA Integration to WBS  d. EBITDA Control Meeting Daily, Weekly, Monthly  4. Documents to be Reviewed in the Field:  a. Project Plan  b. Project Management Plan  c. EBITDA Matrix  d. Capacity Matrix  e. EBITDA Tree  f. Minutes of Meetings |

| 3 |  | Expected preparation before fieldwork:  Written letter for research from SBM ITB - done  Written approval from the company - done |
| --- | --- | --- |
| **Section C. Protocol Questions.** | | |
| 1 | **The Practice in Operation and its innovativeness** | |
|  | a | The practice involves deploying Team Leaders and Experts to the field, preparing to establish an IT system to monitor the program, conducting training, workshops, and accompaniment to implement EVM integration into the Income statement, and conducting regular meetings for EBITDA Control on a daily, weekly, and monthly basis. |
|  | b | The collaborative efforts between the researcher and the company are characterized by the researcher's role as a consultant. This role involves implementing the EVM integration into the income statement and EBITDA integration into WBS in project management. |
|  | c | The idea for the practice started when the researcher experienced the phenomenon of many companies failing to execute their project management profitably. Moreover, the researcher has successfully improved several companies' operational efficiency, productivity, and profitability by implementing EBITDA as the primary KPI while executing project management.  The researcher's ultimate goal is to share this valuable experience and knowledge by writing literature and a dissertation for the Ph.D. program at the Bandung Institute of Technology.  The researcher proposed the integration of EVM, income statement, EBITDA, and WBS. |
|  | d | The Board of Directors has decided to integrate Earned Value Management (EVM) with the Income Statement of the entire Project Management in 2023 |
|  | e | Our proposed integration of EVM with the Income Statement is an innovative practice that sets us apart from traditional methods and positions us for enhanced project management. Currently, project management relies on the EVM system to track project time and costs. However, the system does not provide real-time insights into Project Management Profitability, leading to potential information asymmetry and stakeholder conflicts.  Integration of EVM into Income Statement enables Project Management and other management to understand PM Profitability at any time. Furthermore, Integration of EBITDA into every Work Breakdown Structure (WBS) enables PM to manage and maximize EBITDA daily.  The system will use EBITDA as a WBS Key Performance Indicator (KPI) for PM to focus on and align PM execution while maximizing profitability by optimizing efficiency and productivity in every WBS.  The system will use EBITDA in Every WBS to optimize capacity utilization, efficiency, and productivity. Project Management's EBITDA is maximized daily during PM execution in all WBS. Maximizing daily EBITDA means finding weaknesses in execution and providing feedback for continuous improvement to ensure efficient, productive, and profitable Project Management execution. |
|  | f | The companies’ Board of Directors (BOD) decided to integrate EVM (Earned Value Management) into the income statement and integrate EBITDA (Earnings Before Interest, Taxes, Depreciation, and Amortization) into WBS (Work Breakdown Structure) in 2023. The company then practiced this integration with the management.  As a consultant, the author played a pivotal role in implementing EBITDAMAX in the company, providing expert guidance and ensuring successful integration.  As a Ph.D. student at Bandung Institute of Technology, the author took the initiative to request permission to conduct Case Study Research aimed at improving project management performance and profitability by integrating EVM into income statements and EBITDA into WBS to deliver superior performance to companies.  The Board of Directors permitted the author to conduct the research and collect data for academic purposes only.  In this case, the author received a consultancy fee from the company to improve the Project Management Performance and Profitability. However, the author did not receive any financial support for the research from the company. |

| 2 | **Evaluation of the innovative practice:** | |
| --- | --- | --- |
|  | a | **EVM (Earned Value Management) plays a crucial role in project management execution, emphasizing its importance in delivering optimal performance.**  Project management utilizes EVM to achieve the best performance and profitability. Unfortunately, EVM does not have a profitability indicator.  To enhance the performance and profitability of project management, the company should consider the following:  - Integrating EVM into the income statement  - Integrating EBITDA (Earnings Before Interest, Taxes, Depreciation, and Amortization) into WBS (Work Breakdown Structure)  - Maximizing daily EBITDA in every WBS  To ensure an efficient and productive business process, the company should focus on:  - Overcoming bottlenecks and utilizing overcapacity in the WBS and the project.  To mitigate risks related to dynamic environmental changes, the company should consider:  - Conducting simulations to maximize daily EBITDA in every WBS  During the case study research, the researcher conducted observations, interviews, and surveys for this activity. |
|  | b | The project manager (PM) oversees WBS's daily EBITDA and capacity to ensure optimal performance and profit. |
|  | c | The company focuses on maximizing EBITDA during project execution and daily operations to ensure profitability. EBITDA is a key performance indicator (KPI) for project management (PM) profitability integrated into the Work Breakdown Structure (WBS) in the earned value management (EVM) system. This integration of EBITDA enables PM to enhance performance and profitability. |
|  | d | Contrary to claims from other companies, the researcher has successfully implemented the daily EBITDA maximization method in various industries such as airlines, hospitals, and mining operations. However, it is essential to note that the author did not collect research data during these previous applications. The researcher solely conducted the program as part of management consultancy services aimed at improving company performance.  The author intends to share this valuable knowledge and experience with the academic and practitioner community to encourage collaboration and shared learning.  To facilitate the broader application of EBITDA within WBS and EVM, the researcher is committed to conducting case study research that can be applied to other project management contexts. This commitment reassures the audience about the future of the research and its potential impact on their own practices. |
| **Section D. Tentative Outline for the Case Study report.** | | |
| 1 |  | Audiences for the report include the Doctorate Program School of Business and Management at Bandung Institute of Technology (ITB), the academic community, and the Company Board of Directors. The communication style with ITB and the academic community will be literary for the dissertation and academic literature. The management report will be styled for the company's Board of Directors. |
| 2 |  | The research process adheres to the procedure for Doctorate Research from ITB, the company's rules and regulations, and all applicable laws. This detailed methodology is of particular interest to the Doctorate Program School of Business and Management, Bandung Institute of Technology, and the academic community. |
| 3 |  | The research presents a unique approach to law enforcement practice, which is a key point of interest for the Doctorate Program at the School of Business and Management, Bandung Institute of Technology, and the academic community. |
| 4 |  | Integrating EVM into the Income Statement and EBITDA into WBS will result in straightforward Project Management Executions that deliver superior performance. This system aims to prevent information asymmetry and conflict between stakeholders, as well as facilitate PM and team participation and engagement in achieving performance and profitability targets.  Additionally, the integration aims to enhance the use of project management in other business and management domains.  Many companies have successfully used EBITDA as a primary indicator to increase efficiency, productivity, and profitability in their Management practices to date. |
| 5 |  | The law enforcement agency context and history of the practice: None |
| 6 |  | The exhibits to be developed include:  - The Chronology of events covering the implementation and outcomes of the practice at the company.  - Logic Model for the practice  - Arrays of Presenting Outcome and other data  - Reference to relevant documents  - List of the persons interviewed. |

**Reference:** Case Study Research and Application, Design & Models. (Robert K. Yin,2018)
