# Supplementary material for: Incorporating earned value management into income statements to improve project management profitability and elevate application in the business and management
Source: PLoS One. 2025 Jan 3;20(1):e0312956. doi: 10.1371/journal.pone.0312956 (PMC11698386; doi:10.1371/journal.pone.0312956)
Supplement: S3 Appendix — (DOCX) [file pone.0312956.s003.docx]

S3 APPENDIX 3 SUPPORTING INFORMATION

COREQ (Consolidated criteria for REporting Qualitative research) Checklist

**Incorporating Earned Value Management into Income Statements to improve Project Management Profitability and Elevate Application in the Business and Management**

A checklist of items that should be included in reports of qualitative research. You must report the page number in your manuscript where you consider each of the items listed in this checklist. If you have not included this information, either revise your manuscript accordingly before submitting or note N/A.

| **Topic** | **Item No.** | **Guide Questions/Description** | **Reported on**  **Page No.** |  |  |  |
| --- | --- | --- | --- | --- | --- | --- |
| **Domain 1: Research team**  **and reflexivity** | | | |  |  |  |
| *Personal characteristics* | | | |  |  |  |
| Interviewer/facilitator  Which author/s conducted the interview or focus group? | 1 | The corresponding author conducted the interview or focus group | 13 |  |  |  |
| What were the researcher's credentials? E.g. PhD, MD | 2 | The corresponding author was a Master’s , the co-authors were PhD and Professors in business and management. | 13 |  |  |  |
| Occupation  What was their occupation at the time of the study? | 3 | PhD Student and Professors in business and management | 13 |  |  |  |
| Gender  Was the researcher person or person? | 4 |  |  |  |  |  |
| Experience and training  What experience or training did the researcher have? | 5 | The author was a master’s degree and has 25 years of experience as a management consultant turnaround expert. The Co-Authors were PhDs , Professors in business and management | 13 |  |  |  |
| *Relationship with*  *participants* | | | |  |  |  |
| Relationship established  Was a relationship established prior to study commencement? | 6 | There was no relationship between the interviewer and the participants before the study commenced.  The interviewer was a PhD student, and the co-authors were PhD, Professors in business and management | 13 |  |  |  |
| Participant knowledge of  the interviewer  What did the participants know about the researcher? e.g. personal  goals, reasons for doing the research | 7 | As a PhD student, the author requested permission from the company to conduct research for academic purposes, and they granted me a permit to write academic literature as part of the PhD requirements. The participants are familiar with the interviewer who is known as the company's Turn Around Expert. |  |  |  |  |
|  |  |  | 13 |  |  |  |
| Interviewer characteristics  What characteristics were reported about the inter viewer/facilitator?  e.g. Bias, assumptions, reasons and interests in the research topic | 8 | The interviewer is interested in the research topic and is eager to conduct a case study that relates long-term practical experience with theory.  The authors want to share their knowledge with the academic and practical community. |  |  |  |  |
|  |  |  |  |  |  |  |
|  |  |  | 13 |  |  |  |
| **Domain 2: Study design** | | | |  |  |  |
| *Theoretical framework* | | | |  |  |  |
| Methodological orientation and Theory  What methodological orientation was stated to underpin the study? e.g. grounded theory, discourse analysis, ethnography, phenomenology,  content analysis | 9 | The authors utilized multiple embedded case studies and surveys to gain a thorough understanding of profitability information asymmetry in Project Management using EVM systems. This information asymmetry could lead to conflicts between management and stakeholders. The approach involved comprehensive qualitative and quantitative data collection and analysis.  The multiple embedded case study focused on Shipbuilding and Aircraft Manufacturing Companies that integrated EVM with Income Statements and EBITDA into WBS to enhance Project Management Performance and Profitability.  Simultaneously, multiple embedded case studies were carried out in two project-based organizations in the defense industry. In 2023, the first company was a leading shipbuilding company with 25 projects, including warships and submarines. The second company was an aircraft manufacturing company with 20 projects including helicopter and rocket projects.  . |  |  |  |  |
|  |  |  |  |  |  |  |
|  |  |  | 13 |  |  |  |
| *Participant selection* | | | |  |  |  |
| Sampling  How were participants selected? e.g. purposive, convenience,  consecutive, snowball | 10 | The author utilized qualitative research methods employing interviews. They employed purposive sampling and interviewed a total of 20 individuals from Shipbuilding and Aircraft Manufacturing Companies. This included individuals such as the CFO, COO, Executive Vice Presidents, Vice Presidents, Project Managers, and Managers involved in the program. Prior to the interviews, the author meticulously prepared a protocol for the case study, offering a detailed explanation of the process.  The interviewees represented various departments including Production and Operations, Project Management, Production Planning and Control, Finance, Accounting, Engineering, Human Resources, and Information Technology. In the interviews, there were eleven participants from Aircraft Manufacturing Corporations and nine from Ship Building Corporations, comprising of twenty interviewees.  Quantitative Method by Survey:  - Ship Building and Aircraft Manufacturing: 220 Participants | 14 |  |  |  |
|  |  |  |  |  |  |  |
|  |  |  |  |  |  |  |
| Method of approach  How were participants approached? e.g. face-to-face, telephone, mail,  email | 11 | The author communicated the research purpose to the interviewees through face-to-face interviews, emails, WhatsApp messages, and phone calls. |  |  |  |  |
|  |  |  |  |  |  |  |
|  |  |  | 14 |  |  |  |
| Sample size  How many participants were in the study? | 12 | Qualitative Data Analysis (Interview):  The author used qualitative research methods, such as interviews, and employed purposive sampling to interview 20 individuals from Shipbuilding and Aircraft Manufacturing Companies.  Quantitative Data (Survey):  - First Batch: 220 participants from Ship Building & Aircraft Manufacturing  - Second Batch: 125 participants from Ship Building & Aircraft Manufacturing | 14 |  |  |  |
| Non-participation  How many people refused to participate or dropped out? Reasons? | 13 | For Interviews, we  sent 30 invitations to two companies  We selected the participants from all position levels and decided that 20 participants from different levels of the company's organization would be sufficient. Ten people unable to participate due to time and schedule constraints | 14 |  |  |  |
| *Setting* | | | |  |  |  |
| Setting of data collection  Where was the data collected? e.g. home, clinic, workplace | 14 | The author gathered both qualitative and quantitative data for the research. Qualitative data was obtained through observations and interviews at aircraft manufacturing and shipbuilding companies, as well as through meeting minutes from the corporate office. Quantitative data, on the other hand, was collected through online surveys and interviews via Zoom. | 14 |  |  |  |
| Presence of non-  Participants  Was anyone else present besides the participants and researchers? | 15 | During the interview, only the interviewer and the interviewees were present. However, during observations and group discussions, there were non-participants in the room who were company employees not assigned to the project. |  |  |  |  |
|  |  |  | 14 |  |  |  |
|  |  |  |  |  |  |  |
| Description of sample  What are the important characteristics of the sample? e.g. demographic  data, date | 16 | Interview data:  1. COO (Person, master’s degree)  1. CFO (Person, master’s degree)  1. EVP (Person, master’s degree)  9 V.P. (1 Person with a Doctorate degree, 5 Person with a master’s degree, 3 Persons with bachelor’s degrees)  8 Managers (1 Person with a master’s degree, 7 Persons with bachelor’s Degrees) |  |  |  |  |
|  |  |  | 14 |  |  |  |
|  |  |  |  |  |  |  |
| *Data collection* | | | |  |  |  |
| Interview guide  Were questions, prompts, guides provided by the authors? Was it pilot  tested? | 17 | The researchers provided a question guide along with the survey questionnaires for the structured interviews. For the unstructured interviews, the interviewees freely expressed their opinions about the program. A Case Study Protocol was prepared by the author, and the questions were tested during discussions to understand the program's application for the research project. | 14 |  |  |  |
| Repeat interviews  Were repeat interviews carried out? If yes, how many? | 18 | Repeat interviews with the same person were not conducted. Structured interviews involved asking the same standardized questions to different interviewees. | 14 |  |  |  |
| Audio/visual recording  Did the research use audio or visual recording to collect the data? | 19 | During the interviews, the author used audio-visual recording via Zoom to gather data. Before recording, the author informed the interviewees that the interview would be recorded and obtained their consent. | 14 |  |  |  |
| Field notes  Were field notes made during and/or after the inter view or focus group? | 20 | . Video conferencing was utilized during the interviews, enabling the taking of meeting minutes during the session. | 14 |  |  |  |
| Duration  What was the duration of the inter views or focus group? | 21 | Interviews and discussions typically lasted 30-60 minutes, and participants did not receive transcripts for review. | 14 |  |  |  |
| Data saturation  Was data saturation discussed? | 22 | The author did not discuss data saturation. | 14 |  |  |  |
| Transcripts returned  Were transcripts returned to participants for comment and/or correction? | 23 | However, participants did receive transcripts for review. | 14 |  |  |  |

| **Topic** | **Item No.** | **Guide Questions/Description** | **Reported on**  **Page No.** |
| --- | --- | --- | --- |
|  |  |  |  |
| **Domain 3: analysis and**  **findings** | | | |
| *Data analysis* | | | |
| Number of data coders  How many data coders coded the data? | 24 | There are 13 data coders that can be found in the following coding tree. |  |
| Description of the coding  Tree  Did authors provide a description of the coding tree? | 25 | Project Management   - EVM   - WBS     - Work Package   Income Statement   - Revenue - Costs   - Operating Costs     - Direct Operating Costs (DOC)       - DOC Variables       - DOC Fixed     - Indirect Operating Costs - EBITDA |  |
|  |  |  |  |
| Derivation of themes  Were themes identified in advance or derived from the data? | 26 | The Themes were identified in advance | 14 |
| Software  What software, if applicable, was used to manage the data? | 27 | To the qualitative data analysis from the interview result the author used NVivo Software  For Quantitative data analysis from survey the author used SPSS and PLS SEM | 14 |
| Participant checking  Did participants provide feedback on the findings? | **28** | Ye the participants were provided feedback on the findings during the project/ program briefing | 14 |
| *Reporting* | | | |
| Quotations presented  Were participant quotations presented to illustrate the themes/findings?  Was each quotation identified? e.g. participant number | 29 | The quotations from the participants were used to explain the findings from the interview results. Here's the evidence: The participants provided specific examples to demonstrate the importance of EBITDA as a proper primary Operations/ Project Management Performance Indicator, which were identified by the researchers. The participants conveyed their perspectives using standard language, adding depth and authenticity to the research. This made the findings clearer and more relatable by showcasing how they were grounded in the participants' actual words. |  |
|  |  |  |  |
|  |  |  | 15 |
| Data and findings consistent  Was there consistency between the data presented and the findings | 30 | There was consistency between the presented data and the findings | 15 |
| Clarity of major themes  Were major themes clearly presented in the findings? | 31 | The major themes highlighted in the research findings. | 15 |
| Clarity of minor themes  Is there a description of diverse cases or discussion of minor themes? | 32 | Some cases are described, and minor themes are discussed. For instance, the application of EBITDA in Project Management in shipbuilding varies slightly from that of a ferry company to that of an aircraft manufacturer due to different corporate cultures. | 15 |

Developed from: (Tong et al., 2007): a 32-item checklist for interviews and focus groups. *International Journal for Quality in Health Care.* 2007. Volume 19, Number 6: pp. 349 - 357

**Once you have completed this checklist, please save a copy and upload it as part of your submission. DO NOT include this checklist as part of the main manuscript document. It must be uploaded as a separate file.**

TONG, A., SAINSBURY, P. & CRAIG, J. 2007. Consolidated Criteria for Reporting Qualitative Sutdies (COREQ): 32-item checklist. *International Journal for Quality in Health Care,* 19**,** 349-357.
