# Supplementary material for: Incorporating earned value management into income statements to improve project management profitability and elevate application in the business and management
Source: PLoS One. 2025 Jan 3;20(1):e0312956. doi: 10.1371/journal.pone.0312956 (PMC11698386; doi:10.1371/journal.pone.0312956)
Supplement: S6 Appendix — (DOCX) [file pone.0312956.s006.docx]

**S6 APPENDIX 6 SUPPORTING INFORMATION**

**INTERVIEW INTERVIEWEE B**

Interviewer

Good morning, Interviewee B.

Interviewee B

Good morning, Sir.

Interviewer

We will start. We'll use this interview as a case study. Case study of how COMPANY implements EBITDA and is integrated into earned value management for project management at COMPANY. Because it is essential so far, many people outside the project do not understand its profitability. Then project management is project management managed by COMPANY. Earned value management is the earned value management implemented at COMPANY for project management. Then the WBS is the work breakdown structure that is applied to the implementation of project management with earned value management. Then the work package is the work package for WBS. EBITDA is earnings before interest tax depreciation and amortization. And EBITDA margin, you already know, Sir. Interviewee B is the Head of the accounting division. In this question, we use the 7 Likert scale. Scale 1, strongly disagree. Scale 7, strongly agree. Then if the scale is 2, disagree. Scale 3, somewhat disagree. Scale 4, neutral. Scale 5, somewhat agree. Scale 6, agree. Scale 7, strongly agree.

Let us start with the First question, Sir. EBITDA is a proper measure of profitability of project management because EBITDA is operational profitability or operating profit before payment of interest, taxes, depreciation, and amortization. And EBITDA is not affected by interest, tax, depreciation, and amortization. And EBITDA is only affected by project management efficiency or productivity. What opinion, Sir?

Interviewee B

OK, Thank you, Interviewer. Regarding question number one, I totally agree, Sir. Related to this question, is EBITDA a good profitability indicator as Interviewer's explanation or what?

Interviewer Yes, please.

Interviewee B

Oh yeah, OK. Thank You. So, I strongly agree with this number one statement because EBITDA is one of the benchmarks in measuring project productivity. Where we get EBITDA from revenue minus all operating costs, both are direct operating costs and indirect operating costs. So, EBITDA is formed from the pure results of the company's operations. And this EBITDA cannot be modified by a financial engineer, Sir, and related to this EBITDA. So that EBITDA is fully there to improve it, one of which is the efficiency and productivity of project management. So that the more efficiently the costs are used, and the higher the project's productivity, the EBITDA will increase. EBITDA is also entirely in control of the company's operations and management operations within the company. For example, at COMPANY, we have several directorates directly related to

production, namely the production directorate, aircraft services or MRO, and the technology and development directorate. And to increase this EBITDA, we can also use several methods to increase it with Toyota Ways and others. So related to this point, Sir, my answer is very agreed to use EBITDA to measure project management profitability.

Interviewer

OK, Interviewee B, thank you. And thanks for the explanation too. Second question. EBITDA is the proper measure of project management profitability because EBITDA is under the control of the project manager and his team. Opinion, Sir?

Interviewee B

OK, Sir. Regarding question number two, I agree, Sir. Indeed, we have several measures that can be used to assess profitability, whether using return on equity or assets, and others, gross profit margin or net profit, for example. But for project management, I think EBITDA is the closest and can better describe project performance. Because EBITDA itself is formed from company operations free from external factors, it can describe the pure value of the results of its project management operations. This EBITDA will be one of the tasks of the project manager to monitor and control the project's performance, one of which is by monitoring the EBITDA delivery routinely, either daily or weekly, for example, or daily. And this is very useful to do, because if there is a cost that exceeds the target in one process activity, or there are obstacles in productivity, for example, there is a stopped operation or something else, it can be followed up immediately so that this EBITDA target for the project can be achieved.

Interviewer

OK, Sir, the scales are 6, 7, 4, 3,

Interviewee B Six Sir

Interviewer

Six Sir. Good. Third, companies can integrate EBITDA into earned value management by determining revenue from the budget. So, for example, for the Aero=structure of Airbus, the budget we are given is income. And this is not a cost, but the Company's income, income plan, then deducting the new EBITDA, we can use project costs. So, we insert the EBITDA first. According to you?

Interviewee B

Yes, OK, Sir, for number 3, this is my answer to scale 6; Sir, I agree. So, the explanation is that in implementing EVM by integrating EBITDA, then in a project before the operational activities are carried out, it is necessary to set revenue targets and target costs for each activity. We also target the EBITDA and profit that will be achieved for the project. This EBITDA achievement will be a reference for the project manager in carrying out activities and monitoring using the EVM method periodically. So, integrating EBITDA into EVM can be done and is one of the right ways to control and improve project performance.

Interviewer

OK, Sir, what was the scale?

Interviewee B Six Sir

Interviewer

Six, Sir, OK. Next, the company can integrate EBITDA into EVM by setting the EBITDA margin of the project. Then this EBITDA margin is spread out and applied across all WBS and Work packages. Your opinion?

Interviewee B

Good. For number 4, my answer is a scale of 7, Sir. I totally agree with this presentation. So, to implement EBTIDA into this EVM, we must do cascading first, Sir. From the highest level, general level, to the WBS level or work package. And we target the EBITDA margin for each of these levels. Then we conduct routine control and monitoring. And it is hoped that with the control and monitoring at the WBS level and this work package, the overall project target can be maintained, and we can achieve it. Also, detailing this will make it easier for the project manager to find out which activities have problems in the WBS or in which work packages there are problems, and corrective action can be taken immediately to solve them. So, this time it is essential to do

Interviewer

All right, what is the scale, Sir?

Interviewee B 7 sirs.

Interviewer

7, Sir, thank you. Then the next question is whether companies benefit from using EBITDA to measure project management profitability. Because EBITDA is a standard measure of operating profitability in financial statements. Thus, the company's management can understand the profitability of the project. Because if there is only an EVM with indicators of operations, many managers or management do not know the project's profitability. Please, Sir, what do you think?

Interviewee B

Yes Sir. The answer I agree with, Sir, related to this. EBITDA is a common indicator used in every financial statement and by management to understand and measure project profitability. If this EBITDA is regulated or managed, it will still be reported, Sir. And this is a general size, yes. And in state-owned companies, for example, in COMPANY, EBITDA is one of the KPI targets in management contracts because EBITDA considers that this EBITDA describes us as a business company that can be fully controlled by management. And need to be accountable to shareholders. And this is, and apart from that, EBITDA is also one of the factors considered by

investors to cooperate or invest. Because it better describes the real project performance conditions compared to when we look at it from the net profit side, Sir.

Interviewer

OK, Sir. OK, what was the scale, Sir?

Interviewee B

On a scale of 7, Sir. Interviewer

Scale 7 is fine. Then question number 6, the company benefits from using EBITDA to measure project profitability. Because then the project manager and team know the target, and they can maximize the project's profitability. Your opinion?

Interviewee B

Yes, I agree very much, Sir. Because this is like what I said before, this is like a reference, right? What is the name of guidance for the project manager? So, one of the benefits of using EBITDA is that we can monitor project profitability so that project managers and teams can find out the achievement of cost and revenue from each project and can formulate strategies to be taken to carry out cost efficiency and increase productivity as an effort to maximize the profitability of the previous project. These things make EBITDA a standard measure used by companies to measure the performance of a project.

Interviewer

Good. So, what's the scale, Sir?

Interviewee B 7 sirs.

Interviewer

7 Fine. The next question is, to increase support from supporting units to project management, the project manager needs to report the actual EBITDA achievement compared to the target to the supporting units. So that those who are not involved in the project can help him. What do you think?

Interviewee B

Good. For point 7, this is my answer to point 6, Sir (agree) Sir. So related to reporting actual EBITDA achievements and EBITDA targets to the company's supporting units, this is one of the efforts to increase cost awareness of each supporting unit so that it can support the company by carrying

out supporting actions, for example, with cost efficiency and helping expedite project completion so that it is on time, on cost, and quality. An example is, for example, the timely procurement of materials at the most competitive prices or speeding up project administration processes, for example. And all the support from this supporting unit can help the company achieve the EBITDA target we have set. So, Sir.

Interviewer

What's the scale, Sir?

Interviewee B At 7 sirs.

Interviewer

7 sirs. Good. Then the last structured question. To make it easier for the Board of Directors of one director to provide timely strategic decisions to project management, the project manager needs to report the actual achievement of EBITDA and targets to BOD so that they can provide direction and strategic decisions which are often beyond the project manager's victory. What do you think?

Interviewee B

Yes, for point 8, my answer is scale 7, Sir. Strongly agree. So, reporting the actual achievement of EBITDA and EBITDA targets to the BOD, the Board of Directors, is very important to do, Sir, because we need to report related to the performance of a project on the target that has been set. So that if there is a gap in the target and realization that occurs, it can be informed to the board of directors, and if there are things that cannot be done or resolved by the technical team or under the board of directors, it can immediately get a resolution from the board of directors Sir, both strategic and other. So that by reporting these matters too, BOD can make the right decisions, Sir, in determining strategies and policies to resolve these problems. And reporting on the actual achievement of EBITDA and EBITDA targets to BOD needs to be done regularly. So that BOD can also follow up on solving problems that previously occurred.

Interviewer

OK, Sir. What's the scale, Sir?

Interviewee B

Seven Sir, it is essential Sir this Sir. Strongly agree.

Interviewer

Now the structured question is done. I ask for your opinion about the benefits of EBITDA integration in project management and for project-based companies like COMPANY. What is it, Sir? So, please tell me freely, Sir. Please.

Interviewee B

OK, Sir. Good. So, the COMPANY itself is based on a project, Sir, not only related to financing and other things, but we also live from the project. So, this project needs to be maintained, and a system is implemented to control and monitor it so that the project we get can follow business events because we use business events, Sir. Well, what happened before was that we reported it after everything happened, Sir, at the end of a month. Now, with regular EBITDA reporting, what will it be? It's not surprising in the end, Sir. We immediately found out that.

Interviewer

You can prevent that.

Interviewee B

It can be prevented, Sir. So, we can immediately be informed that EBITDA, Daily EBITDA, is what we call joint control, Sir, related to the realization of costs in each project activity. So doing Daily EBITDA will help the company achieve the expected project performance. But it's not only related to the financial side, but this also must pay attention to safety, then still pay attention to the quality of the project, as well as the quality of the project, Sir. And it also does have an impact later, in the end, is timely delivery, that's Sir. So, applying EBITDA for project-based is essential, Sir, because it was our joint control earlier.

Interviewer

Yes, earlier, you said that maximizing a project's EBITDA must still be in the corridor of maintaining target delivery, quality, and safety. So, we play in an arena that is constrained. But in this way, the project manager can maximize its performance and business processes as well as possible in this corridor. Your opinion? Please, Sir.

Interviewee B

Right, Sir. So indeed, with this implementation, it does not mean that we also ignore other things, Sir, which are equally important in terms of quality as well as the previous one. So, this EBITDA must have considered the QCD matters that were discussed earlier, Sir. And to maintain our performance according to business events or exceed targets in business events, control is needed. The control is the most possible. The one that best describes it is by using the EBITDA that was

So, if we can control according to what is in the business study, automatically the quality matters, related to quality matters, are related to what was called earlier.

Interviewer Yes

Interviewee B

Safety and delivery will be achieved, Sir. Because this Business Study is our standard reference, Sir. Just control what to use. Yes, EBITDA was under our control.

Interviewer

OK, maybe the last one, I ask you to convey your expectations with implementing EBITDA in the Earn Value Management Project Manager. What are your hopes for PDI, Sir?

Interviewee B

All right, my hope is that we need to make continuous improvement, Sir. So, if we already have a previous report card, Sir, continuous improvement can be made. We can evaluate if we have a report on our previous performance. By implementing this, EVM, we can see and evaluate which sides can increase productivity. Or which side of the cost we can do efficiently. I hope that this implementation will open one's eyes and guide fellow project managers to continue to make continuous improvements. So that the project that we get later will have a significant impact on the COMPANY, Sir. Go, Sir,

Interviewer

It's finished, Sir; please allow me to close the recording.

Interviewee B OK, Sir.

INTERVIEW INTRVIEWEE C

Interviewer

All right, Good evening Intrviewee C

Interviewee C

Good evening sir

Interviewer

In this interview, I'll explain first some of the terminologies. As we know that Company is a project-based company or project-based corporation; therefore, COMPANY needs to increase EBITDA Project Management (PM). Now, to determine management's understanding of EBITDA integration into information systems and Earned Value Management.

PM is Project Management managed by COMPANY.

Then, EVM or Earned Value Management is the applied EVM for PM. WBS or Work Breakdown Structure is the WBS implemented at COMPANY. And work Package is a work order used for WBS Earned Value Management.

EBITDA is income before Interest, Tax, Depreciation, and Amortization payments. And EBITDA Margin is EBITDA divided by revenue.

We have two sessions, sir; the first session is a structured interview, the questions are structured, and in the second session, you are free to talk about the implementation of EBITDA in EVM and what the Company hopes to implement later in the second session.

Interviewer

So for the first session, that's the question.

Implementing EBITDA in PM using Earned Value Management and WBS, questionnaire using seven on a Likert scale.

Scale 1 to 7. Scale 1 strongly disagree - Scale 7 Strongly agree.

Among them, scale 2 - disagree, Scale 3 somewhat disagree, scale 4 neutral, scale 5 somewhat agree, scale 6 agree, and scale 7, strongly agree.

Can I start with a question, sir?

Interviewee C

Interviewer, I'm sorry; I'm just asking permission for a moment, sir.

Interviewer Okay, okay

Interviewee C

I changed my clothes because you wear formal clothing (laughs). Okay, sir, I'm ready, sir.

Interviewer

Okay, let's start, sir.

No. 1, EBITDA is the proper measure of PM profitability because EBITDA is operational profitability before interest payments, taxes, depreciation, and amortization. And the value of EBITDA is not affected by interest expense, taxes, depreciation, and amortization. Because it doesn't include it

And the EBITDA Value is only influenced by the efficiency and productivity of PM. Disagree or agree, sir?

Interviewee C

I totally agree, Interviewer,

Interviewer

All right, sir, scale 7, yes, sir

Both EBITDA is an appropriate measure of PM profitability because EBITDA PM is entirely under the control of the Project Manager and Team.

Meanwhile, if there is EBIT, there is depreciation and amortization that is beyond the control of PM. Strongly agree or strongly disagree.

Interviewee C

I'm a 6, sir; I agree on a scale of 6.

Interviewer

Okay, sir. Then six means agree, sir.

Then statement 3. The company can integrate EBITDA with EVM by setting a Revenue Plan from the Budget So if Airbus provides the Budget, Boeing will become the planned revenue from the Project. Then set the EBITDA plan. And the income plan is reduced by the EBITDA plan, becoming a cost plan for PM.

Agree or disagree, sir?

Interviewee C I agree sir

Interviewer

Agreed, okay, sir Then, number 4,

The companies can integrate EBITDA into Earned Value Management in PM by setting a Target EBITDA Margin in PM, then down to the work package in each WBS. So each one is given an EBITDA Margin target. This distribution of EBITDA Margin is the way to integrate EBITDA into EVM.

Your opinion?

Interviewee C I agree sir

Interviewer

Agreed; what's the figure, sir?

Interviewee C

Oh, 6, sir, sorry. Agree.

Interviewer

Okay, 6. Question 5 Companies benefit from using EBITDA as a measure of operating profitability PM because EBITDA is a standard measure of operating profitability in financial statements.

So that if there is EBITDA in PM, other managers who are not in PM can know. Know project profitability because, without standard profitability measures, we use PM terminology that many managers don't understand the actual project profitability.

Interviewee C

Agree,

Interviewer

What's the scale, sir?

Interviewee C Seven sir

Interviewer

We can continue with question 6

Companies benefit from using EBITDA as a measure of PM profitability. Because then the benefit is that the Project Manager and Team know their respective EBITDA targets and can maximize PM profitability because those who can increase PM profitability are the Project Manager and its Team.

Do you agree or disagree with the benefits?

Interviewee C I agree sir

Interviewer

What's the scale, sir?

Interviewee C

6. 6 sir Interviewer

Interviewer

Then question seven. To increase support from supporting units at COMPANY to PM, the project manager needs to report the actual achievement of EBITDA and targets to other management. So other management knows what the Project's profit or loss is like?

So that it can provide support to PM if needed. Agree or disagree

Interviewee C

Agreed, six sirs, on a scale of 6

Good. And this is the eighth, sir. To obtain timely support from the Board of Directors and provide direction to PM, the project manager must report actual EBITDA achievements compared to the EBITDA Target. In this way, the BOD can promptly provide guidance and strategic decisions, which are often beyond the project manager's authority.

Interviewer

According to you?

Interviewee C Strongly agree

Interviewer

What's the scale, sir?

Interviewee C

7 Interviewer

Interviewer

This structured one is finished, sir. The score is A plus, sir (Laughter) Okay

Now please tell me. By integrating EBITDA with EVM in PM. What do you hope for the implementation of EBITDA throughout COMPANY? What are your hopes like?

Interviewee C

Yes, the hope is that every team involved, both direct and supporting units, understand the details of the activities, including the costs and the desired revenue target.

Well, of course, we must do it intensively. For this, agree with daily control.

We don't want everyone to be surprised after the Project's completion and realize that we do not achieve the final target,

So the hope is that EBITDA management will occur in detail from our fellows, and there will be daily control. Why is that so that the recovery and action can be faster? So what is experienced today can be achieved or not can be improved by tomorrow's action. Now this is not only PM but also supporting units that need support that can also take action. Let's know the progress.

That's what I think is the hope for implementing EBITDA in PM.

So far, the focus of our fellows has been a bit more on achieving the time to finish the Project. With EBITDA implementation, of course, everything has been summarized, sir. Is it cost-wise or time-wise

That's our hope, Pak Interviewer.

Interviewer

Good. If there is a project, the strategic value is the same, and the EBITDA target is higher than the other. Can you use EBITDA as a decision-making tool for portfolio management? Which one comes first? Which resources for which projects are essential? Can EBITDA be used for that portfolio management, sir?

Interviewee C

Suppose it is necessary to select the determination of resources. Of course, there are considerations, sir. The first is that whatever Project we do, we agree that it must bring good profitability.

However, of course, we also have to consider projects whose impact is not only profitability, namely the company's sustainability. We must consider EBITDA when we should share the resources. In my opinion, because the COMPANY is in the aerospace sector, we need to consider sustainability.

That's what we think. Because by coincidence COMPANY has a development project in nature, there is a serial one. We still hold on to profitability for those developing but want it sustainable.

Interviewer

Good. Then, are there other things regarding the implementation of EBITDA to EVM? What program is the COMPANY working on right now? Is there any training? Who did the training, then who made the WBS, and how to apply EBITDA to the work package? Can you tell me, sir, for a moment?

Interviewee C

Okay, what's underway is to complement our fellows engaged in projects on their knowledge, PM, sir. PM with EBITDA. Because, of course, to carry out knowledge, our fellows have to be able to do it first; that's the first thing. We are currently training, and of course, the more people can be trained, the more they will know about it.

The second is to complete the training for PM, specifically for Project Managers; we integrate EBITDA with WBS, right, sir, the Work Breakdown Structure, which currently exists at COMPANY using SAP.

We also call Project Management at SAP. In that area, our term is in production called MBOM, but we must complete some work in an end-to-end project after the completion of the aircraft production.

Now we are completing this to become a complete WBS from pre-sales activity to post-acceptance by customers. Now this is what all units are currently involved in to achieve this.

So we have a complete WBS of PM implementation related to EBITDA and EVM so that the integration of EBITDA with EVM becomes optimum.

That's what we do now.

Apart from that, we are making parallel improvements to the existing processes. Because, after all, to manage the Project and the cost, we need to see how to control the costs. We are seeing which processes are value-added and which are non-value-added. Then the PM processes improvement is not only in production but also in the supply chain must be improved.

Interviewer

Then, what is the importance of IT for aircraft projects where there may be thousands of work orders? Please tell me, sir.

Interviewee C

Okay, so, to work on one plane, we must involve 15,000 to 17,000 orders, sir. That's the first complexity. The second complexity is the geography and organization involved work area. If we look at it from aircraft manufacturing, the work area is spread out. So that the speed of information for consolidation, which is a daily movement in nature, is impossible to do manually. Information Technology is essential here; the COMPANY already has a Project System.

Currently, our challenge is to improve the discipline of the end users so that the purpose of the presence of IT is to help accelerate the distribution of information to the right people accurately and timely. So basically, IT is an essential tool for implementing Project Management.

Interviewer

Okay, sir. What about MRO, sir? Implementation of EBITDA in MRO, as in GMF, and so on? Please, sir.

Interviewee C

Yes, it's similar, sir.

MRO at COMPANY has the same complexity. So there are projects such as aircraft restoration and aircraft modification. In terms of cost, it requires the implementation of PM. In the context of time, there was work that required eight months, some one year, and cost management was also quite large. So PM for MRO must also integrate the EBITDA with EVM so that the Project can get the profitability we expect.

Interviewer

Then, what is your target for implementing EBITDA maximizing in this Project? What is the EBITDA Margin target that you expect?

Interviewee C

Yes, COMPANY currently has how many portfolios. We are targeting above 20% EBITDA Margin, sir. If only this year we can reach 25%. But this is indeed challenging. Some of the challenges are the availability of

resources that we must complete. But with the implementation of good PM. With the performance of a good EBITDA project, we are trying to achieve the EBITDA target above 20% or if it is 25%.

Interviewer

Our interview is over, so I turned off the recording, sir.

Interviewee C Yes

INTERVIEW Interviewee D

Interviewee D

We will turn it off first after the video because we are worried the signal will go up and down.

Interviewer

All right, sir, let us start.

Interviewee D Please sir

Interviewer

Interviewee D, thank you for your presence and approval in this interview. We will use this interview as material for research for the case earlier. The application of EBITDA in Earned Value Management project management COMPANY, which uses the IM4 information system. Then the explanation, project management is project management activities, starting from contracts to engineering, then up to the logistical requirements of the implementation, delivery to warranty operations, but not including the work results of COMPANY. Whether the COMPANY ship is operating successfully or not does not have that, sir. Then Earned Value management is what is now implemented at COMPANY with IM4 to manage the entire project. Then the WBS is the Work Breakdown Structure applied to the management project. Then, Work Stages are work orders used to carry out WBS then. EBITDA is earnings before interest, tax, depreciation, and amortization, namely profit income before paying interest expense, tax burden, depreciation, and amortization. At the same time, the EBITDA margin is the percentage of EBITDA divided by revenue.

For this interview, I also used a Likert scale of seven scales. If you agree with the statement that I convey, you can choose the level on which scale the number is. If you strongly disagree, the score is one. If you strongly agree, the score is seven, so the plan is from a scale of one to a scale of seven, a scale of two disagrees, a scale of three somewhat disagrees, a scale of four is neutral, a scale of five is somewhat agreed, a scale of six agrees, a scale of seven strongly agrees. Let us start, sir. We start the interview. EBITDA is a measure of profitability from proper project management because EBITDA is operational profitability before paying interest expenses, taxes, depreciation, and amortization. EBITDA is not affected by tax interest and amortization depreciation, but the value of EBITDA is affected by the efficiency and productivity of project management. Your opinion? Please.

Interviewee D

Yes, thank you, Interviewer, we strongly agree. So indeed, EBITDA is an excellent tool for measuring the profitability of a project management activity. So those of us on a scale of seven could not agree more.

Interviewer

OK, thank you, sir. Then EBITDA is an appropriate measure of project management profitability because EBITDA is entirely under the control of project management. From all project management, it can control EBITDA. What do you think?

Interviewee D

Yes, we also strongly agree because aspects outside the control of the project, such as interest, taxes, and amortization depreciation, are indeed the BoD's authority or are outside the control of the project management. So, we also strongly agree with a scale of seven.

Interviewer

Thank you, sir. Then the company can integrate EBITDA into EVM as COMPANY sets a revenue plan and budget that the project owner approves. For example, Indonesia's power provides funding. So, the budget becomes the COMPANY Revenue plan. We can define the project’s costs, and the plan revenue is reduced first by the EBITDA plan. So, the plan cost is different from the budget. There is no profit if the plan cost is the same as the plan revenue. What is your opinion, sir?

Interviewee D

Yes, we also strongly agree because EVM is indeed one of the methods that can be used to control costs and project completion time. So that by managing EBITDA, one side related to cost control efficiency will also be achieved so that, indeed, the integration between EBITDA and EVM is very good in project control so that we can be more thorough in detail. That is our opinion, Pak Interviewer.

Interviewer

OK, Interviewee D, thank you. Then the following statement is that companies can integrate EBITDA into EVM by setting EBITDA targets in each Work Breakdown Structure and Work Package so that implementers know their target EBITDA margin. Your opinion?

Interviewee D

Yes, we agree to be integrated because if it is EBITDA, at least he is very detailed in controlling the cost aspect. from an efficiency perspective. And even if we manage it properly, it will also be related to the schedule. It will also be better to control project goals with EVM in controlling costs and time will also be achieved. I think so, Interviewer.

Interviewer

OK, thanks, Interviewee D. Then the next statement is as follows

companies benefit from using EBITDA as a measure of project management profitability because EBITDA is a standard measure of profitability in financial reports, so if we apply EBITDA in project management, our fellow team and the management can understand because, without this financial indicator, the administration can't find out project profitability immediately, they have to wait for financial reports and so on. Your opinion?

Interviewee D

Yes, we strongly agree. Because indeed, with the EBITDA pattern applied in project management, it means that finance fellows and accounting fellows can directly adopt reporting so that it can quickly be used as input from financial reports according to Interviewer

Interviewer

OK, thank you, Interviewee D. Companies benefit from using EBITDA to measure project management profitability. Because by doing so, the Project Manager and the team can find out the EBITDA target and maximize the EBITDA in its implementation because they have control over the EBITDA. Your opinion?

Interviewee D

Yes, we also strongly agree because, with the distribution of EBITDA targets in all divisions, the first one will raise awareness. The second will also create a sense of control over existing costs, especially if we provide an understanding that this will have a direct impact on, for example, the sustainability of the company as well as those whose personnel income is extraordinary, for instance, with an agreement that savings will result in efficiency returning to the welfare of workers, this is very beneficial for management as well as for the employees themselves. I think so, Interviewer.

Interviewer

OK, Interviewee D, thank you. The following statement is to increase support from supporting units to project management, the Project Manager needs to report the actual achievement of EBITDA compared to the target to the supporting units so that they are aware and helpful in providing support. Your opinion?

Interviewee D

Yes, that is also very appropriate. I strongly agree because the first thing is that people will be aware. They can act according to the needs if they understand the project management target and the achievement. , Should they pursue or maintain, and so on. So, in my opinion, it is very accurate that by providing reporting to the supporting department, you can understand, oh, what support is needed to speed up, whether it is support for accelerating the opening of the credit line, accelerating contracts, accelerating material procurement, accelerating product completion and so on, in my opinion, That's very true, Interviewer. So precisely disclosing this information will generate strong support from some supporters, said Interviewer.

Interviewer

OK, sir, it is exciting; you said sharing this information can awaken a sense of belonging and a sense of togetherness to support. Well then, for the following statement, to make it easier for the Board Of Directors or directors to provide decisions, timely strategic decisions to project management, the Project Manager needs to report the actual achievement of EBITDA and EBITDA targets to BOD so he can give direction, and frequent strategic decisions times beyond the authority of the Project Manager. Your opinion?

Interviewee D

That is also very appropriate because, with the correct information related to the EBITDA, management can make the right decisions. If appropriately managed, the administration can make the right decisions if they get accurate information, including this EBITDA issue.

The management can further seek information about the cause of underperforming Project Management. Does the material aspect cause it, the production growing part, is it from the human resource aspect, so that the management can find out the weaknesses and top management can immediately give direction can provide solutions to problems that may be outside the authority of the project management, I think so, Interviewer.

Interviewer

Thank you, Interviewee D., Structured questions and statements are complete. Now we ask you to please convey. What is your experience, and what are your hopes with integrating EBITDA to EVM with

IM4? What are your hopes for the COMPANY, sir? Please.

Interviewee D

Thank you, Interviewer. So, we hope that integrating EVM and EBITDA with IM4 will make project control more real-time faster and integrate all parts. A successful project has three dimensions: quality, cost, and delivery (QCD). An example is also the HSE aspect.

Now, with the correct information, it will provide an early warning to top management to make decisions on actions to prevent project losses, project delays, or quality failure so that procurement control can only be done if the system developed was structured between EBITDA, and EVM, including IM4.

Interviewer

Both are from the integration of EBITDA-EVM at COMPANY, that you carried out. Very interesting, yes, very interesting because in every COMPANY Work Breakdown Structure, there will be an EBITDA margin so that the fellows who implement the project understand very well, in detail, how, sir, to build a sense of belonging from fellow project implementers in workshops and engineering, then in logistics procurement. How sir? With this integration, sir?

Interviewee D

Now with this system, number one for each department will understand the scope of its work, its progress, and what actions they must complete immediately. For example, how come this project does not progress? Is it because production growth is not ready, the materials are not prepared, the human resources are insufficient, and so on?

Everything can be traced with this EBITDA implementation system and the Work Breakdown Structure. It will be an institution business in the direction of profit and lost continuity from the sustainability earlier. By controlling the EBITDA of the EVM with these IM4 tools, it is an extraordinary combination to manage an enterprise. Moreover, we know that the project's development is a one-time event. There cannot be any delays. We must synergize all resources and balance quality, cost, and delivery time. With these tools, we get helped, Interviewer.

That is my opinion.

Interviewer

COMPANY is one of the state-owned strategic industries that has turned out to be a pioneer in implementing an EBITDA system integrated with Earned Value Management and IT.

This experience includes pioneers, not only in Indonesia but in the world, sir. Your advice to other BOD of parent and sister company, what is your advice, sir? Because with this experience, you certainly can share this valuable experience with other companies, sir,

Thank you, sir.

Interviewee D

Thank you, Interviewer, for accompanying us like this, the first time to our internal COMPANY, that the system we are developing is very complex and sophisticated to control an enterprise.

So, we must understand that this process cannot run perfectly immediately. We must continue to run this process, and we implement it. It will further refine the system that we are developing. So, we must have tenacity, patience, toughness, and agility to continue implementing this consistently and sustainably. We must internally instill something that is not simple, not easy, but thank God, we have started it. So, the hope is that with us consistently implementing this, God willing, this will make the company's conditions better so that externally it can be adopted by similar shipyards and according to our target, later we can synergize multi yards so that various shipyards can be synergized in one this platform. So, the IM4

principle is to synergize engineering design systems, resource management systems, and one more integrated finance system. So, these three platforms, which usually stand-alone, are united in the IM4 platform. So coincidentally, Interviewer also developed the EBITDA one, which is very suitable for controlling the details of WBS projects related to budgetary control, cost, revenue planning, etc. So, this is very complementary to IT extensive system. Then in the project, there is EVM in detail controlled again with EBITDA; now, this is global control, in fact, and more detail. So, if we imagine it like that, the EBITDA will focus on cost control, revenue, earnings, and so on. The EVM is in the direction of cost and the target time for IM4 in general corporate business. So, this is complementary, starting from the available medium to the meticulous details, so all the stakeholders in it must understand it in detail so that if this is integrated, it is extraordinary, Sir. The journey of project and corporate control itself, according to Interviewer, is our opinion.

Interviewer

The case is exciting, wider, sharper, and more in control. Then ask for your opinion by knowing the EBITDA implementation of all projects. What are the benefits for you as a senior vice president in controlling the portfolio of these projects by knowing the EBITDA of each project? What are the benefits, sir? Please.

Interviewee D

So, of course, one thing that matters in improving this system is that we know early on the performance of existing projects. During this period, the EBITDA turned out to be like this. If we control it, we can prevent it better than cure it. Those are the differences in the early detection of our condition. That is why daily improvements can be added up to weekly or monthly, which means that later we are not too late to make decisions or act. Thus, God willing, these projects will become more well-managed, benefiting both the company and the customer. This improvement is a positive cycle of making the project completed on time. The project is profitable, the customer is happy, will provide new marketing, order again, or positively influence prospective customers. I think so, Interviewer.

Interviewer

Excellent, OK, sir. Last message, sir, with your extensive experience, then integration with IM4 and IT because shipbuilding is impossible without a computer, and you integrate EVM with IM4 with EBITDA. Last message, please, sir.

Interviewee D

OK, thank you. We are very grateful to have a visionary CEO by developing the IM4 system, and we are also thankful to meet Interviewer, who has a max EBITDA system. So that is complementary. So, on the other hand, in general broadly, it is controlled in a thoughtful and detailed manner related to earnings before interest, taxes, depression, and amortization.

So, this control system that we can use to control at the macro and micro levels.

This integrated system is an asset for our company, but our homework is to provide an understanding to all existing stakeholders.

Sir, this is what we think should be our achievement together.

How do we consistently implement the IM4 system for EBITDA EVM integration?

So that is my message, and indeed those are the keywords, consistency and sustainability,

In addition, we must develop communication coordination and cooperation in this application. Following Interviewer's suggestions. Agree,

That is, it, Interviewer.

Interviewer

OK, Interviewee D, what an extraordinary interview. Hopefully, it will be helpful for our fellows at COMPANY then in companies in Indonesia and abroad. This knowledge has been applied at COMPANY and COMPANY is an excellent example of an EBITDA- EVM Project Management application, and we thank you, sir.

Interviewee D God willing

Interviewer Yes, greeting.

Interviewee D

May Allah's peace, mercy, and blessings be with you too. Thank you, Interviewer. See you soon.

Interviewer

Thank you, sir.

INTERVIEW INTERVIEWEE E

Interviewer

All right, good morning, Interviewee E. Thank you for your permission for this interview in which we intend to make a case study. How does COMPANY implement EBITDA, integration with EVM, and current COMPANY IT systems? There are two sessions in this interview; the first session is a structured interview where there are statements, from which statements you will choose the answer to agree or disagree, where the scale is 1 to 7. Strongly disagree 1, disagree 2, somewhat disagree 3, neutral 4, somewhat agree 5, agree 6, and strongly agree 7. So, 1 to 7, from strongly disagree to agree Strongly. Can we start, sir?

Interviewee E

Yes, Interviewer

Interviewer

The first statement, EBITDA, is the proper measure of the Profitability of project management because EBITDA is operating Profitability before interest expense, taxes, depreciation, and amortization.

The value of EBITDA is not directly affected by interest rates, taxes, depreciation, and amortization. And the EBITDA value is only influenced by the efficiency and productivity of Project Management.

Your opinion?

Interviewee E

I do agree because the EBITDA value is before interest, tax, depreciation, and amortization. So, we usually say operating profit. So how do we do efficiency, effectiveness, and a business process in the project? Under the scope assigned to the project. That, so I agree that EBITDA is a measure of Profitability in project management.

Interviewer

So, what is the number, sir?

Interviewee E

If the number is 7, I strongly agree.

Indeed, so that we know how much the operating profit is in each project management, EBITDA is the measuring tool at least like that.

Interviewer

All right, let us continue, Interviewee E

EBITDA is an appropriate measure of Project Management profitability.

Because EBITDA is entirely under the control of Project Management and the team. The team consists of Engineering, then procurement, and the coordination to delivery.

What is your opinion?

Interviewee E

I agree if this is the score, 6, but there are still challenges, especially in several organizations in Indonesia and especially in COMPANY Indonesia. In this project management, there are several related stakeholders. So, the Project Manager and Team depend on several other work units, such as the financial or treasury divisions. But agree that EBITDA is a measure of project management profitability. Still, if you say it is entirely under project management control for crucial business processes such as Engineering Production,

Design, Procurement, construction, to quality assurance, it is in the project manager. Meanwhile, it is difficult for the project manager to have full authority in areas related to corporate matters, such as funding and other things.

So, my score is 6, sir. Excuse me.

Interviewer Fine

Third, companies can integrate EBITDA into EVM by setting an Authorized Budget such as the BMPP; the authorized budget is considered a Revenue Plan. Then the COMPANY determines EBITDA, and the rest is the cost plan. So, the budget plan does not spend all the Authorized Budget

Your opinion?

Interviewee E

Agree. If you agree, the point is 7. Because this practice can see the actual project management performance, if the Authorized Budget is implemented, the Project Manager, in this case, the project management manager, can see how much Profitability my project has experienced. From the direct cost side and the indirect cost side, can we see efficiency?

That is, it, sir.

Interviewer Next

Companies can integrate EBITDA into EVM by giving Project Management a margin target, and then each WBS is also tagged with EBITDA Margin, right down to the Work Package.

In this way, everyone who is responsible knows the target and can control their EBITDA. Your opinion?

Interviewee E

Agree on point 6. There is still a challenge here; distributing large chunks of EBITDA into small activities is challenging. But I agree. In concept, I agree that this EVM has been practiced in Project Management; Interviewer, I beg for permission.

It has been practiced, but there is no target profitability; what % should it be?

The framework or mindset of our fellows in Project Management is to have a share of 100, so it is already

100. There is no profitability target; what is the %?

Suppose there is an EVM with an EBITDA Margin or profitability target of what percentage and distributed to each WBS to the slowest level. In that case, it will be suitable for the Company.

I agree, 6. The Work Package has EBITDA Margin attached. But the challenge is quite significant.

Interviewer

The point is, do you agree, sir?

Interviewee E Agree Point 6

Interviewer

Then the Company can benefit from using EBITDA as a measure of Project Management performance because EBITDA is a standard measure of financial Profitability. So Non-Project Management, our fellows can know and understand the Profitability of Project Management in COMPANY.

What is Your opinion?

Interviewee E

Strongly agree, sir totally agree.

If it is attached to a project, even to marketing, it will be easier for the Company to sell in the future. If, for example, we now know that Project a Fast Missile Ship knows its Profitability, it turns out that you can see that the Profitability is 40% during delivery. 40% surplus, we have 40% profit, for example. In the future, there may be a strategy that we target; that is just the market. In the future, that.

So, I totally agree with that, sir. Because later, it can make a company sharp in marketing and business in the future.

Interviewer

Good sound, good, good, good.

Then, the Company can benefit from using EBITDA as a measure of Project Management Profitability because by doing so, the Project Manager and Team know the target, then they can maximize the Profitability of the Project Management.

What is your opinion?

Interviewee E Strongly agree.

So, from the project manager and team side, they can maximize EBITDA.

So far, the PM framework has indicators for the Scope, Costs, and Time. So, in terms of time, it is specific, the target time is already, is that certain, that is absolute.

Then the scope is also absolute according to the contract attached to the project management. The cost so far is also absolute, but only the finish line.

Now, the finish line is pulled a little forward so that you can get what percentage of profit. So, this is important in every project for an efficiency framework or an efficiency mindset for HR in Project Management. That is the first benefit.

The second benefit is for the Company itself. In running a project, the Company also runs its finances, making money guarantees, funding with investors, etc. Profitability in the EBITDA measurement tool, which is commonly used by financial statements, will facilitate funding from the corporate side. Go to projects. So right on target. So totally agree, sir 7 points, sir.

Interviewer Okay sir

Then to increase the support of supporting units for project management, the Project Manager needs to report the actual achievement of EBITDA and the target to the supporting functions in the Company so that he knows the project's Profitability.

Your opinion?

Interviewee E

That is right. Strongly agree, sir, so the point is 7; I totally agree. Because it has been out of the reach of Project Management or Project Managers and Teams are supporting staff outside of production. The production team can perform their work because there is support from facility management.

The actual EBITDA synchronizes oh, it turns out that the costs for direct labor, for example, production costs with supporting staff, can be compared.

So, we can use EBITDA as a measuring tool; what contribution does the supporting unit have? Furthermore, management can use EBITDA as the basis to distribute bonuses. For example, if the project turns out to have a profit of 40%, then you know how many of the supporting people have contributed to the project.

Management can use the EBITDA indicator as a trigger for supporting our fellows.

The achievement of Project Management EBITDA is not solely by the production but also by the supporting units. The Supporting units can contribute some percentage to the accomplishment of PM EBITDA So; the right measurement tool is EBITDA.

I strongly agree, sir.

Interviewer Okay sir

Then the last. To make it easier for the Board of Directors or Directors to make timely strategic decisions to Project Management, the Project Manager needs to convey the actual EBITDA and EBITDA targets to the BOD so that he can provide decisions or directions for strategic decisions, which are sometimes outside the authority of the Project Manager, sir.

Interviewee E

I agree, sir. So, this is part of the Project Management framework at PMBOK, Stakeholders management. Indeed, it has been done in the form of an S-Curve. But in general, globally, the S-Curve is below its achievements Planned. Now this is important to convey on the process side.

So, on the other hand, there are things that the Project Manager cannot touch, which we discussed at the beginning-namely Finance. If the Project Manager knows that the Project EBITDA is minus so much USD, he can report to the Board of Directors for consideration.

Oh, this project turns out the possibility for profit is difficult. So, what is the strategy being conducted in the existing?

For example, when a company is looking for additional investors or new funding, or Joint Operations, management can use EBITDA as an indicator to get financing.

Furthermore, management can use the EBITDA indicator for the Business Strategy Marketing Strategy. Oh, it turns out that the project we are going through in 2022 has terrible profits, for example, only 1%. Do we need to repeat this? That is strategic reasoning in Top Management. So, this EBITDA measurement tool is essential to work progress. I really agree, sir. To facilitate the Board of Directors.

So far, in all the work that is like an EPC company, what is visible is only the progress of the work. Oh, delivery, delivery, it is from the work side. From a cost standpoint, it is sometimes out of reach. Well, this is very agreeable to apply, sir.

Interviewer

All right, Interviewee E.

So, the Structured interview is over, then now the Un-Structured Interview. Next,

Please let me know your hopes and opinion on integrating EBITDA with EVM supported by Company's IT for Company's progress.

Please sir

Interviewee E

Thank you, Interviewer

When it comes to needs and expectations,

We first need accurate time data, which we currently target in our digital platform as an IT SYSTEM.

Now, we are already running in phase 3. Thank God it is integrated. So, Man-Hours become Direct or Indirect Costs in a project, including the materials. That is our first need, according to actual data.

Then the hope is that after we collect the data successfully, the data will become input for us all, especially for Corporations in COMPANY Indonesia to become a mature strategic plan as we advance.

So, choosing projects in the future will be based on actual data. Well, that.

Related to that, another requirement is that the Earned Value Method is consistently implemented in every project. Because each project has a different style, small and large projects may have other weighted Earned Values. So, this is what is needed in the future in digitalization, Interviewer.

In addition, the need for real-time data can be enjoyed or seen by Top Management, Middle Management, and the Lower management level at any time. Therefore, the strategic directions and directions from the current BOD are pretty good. So that everyone holds the Gadget. So, everyone can see the EVM of each project. So, Interviewee E is willing, and it will be a good benchmark in the future. In the end, the project data is actual; the data can be absorbed, seen, and used to forecast and become material for future strategic plans. It is Interviewer.

Interviewer Good

EVM does not have an indicator of Profitability.

Interviewee E That is right.

Interviewer

With integration with EBITDA and maximizing Daily EBITDA, what are your benefits and expectations for Company B?

Interviewee E

The benefit is that we know that each project has a profit or a percentage of Profitability. That is the first thing that matters.

So that we know that both big and small projects are per the contract value, that is us.

Oh, this project is profitable; this project is not very promising. This project is not very promising but strategic in the government; this project is good but carries risks.

So, EBITDA becomes our basis for conducting what projects we will take on. So, we hope that EBITDA will stick to EVM in every WBS and Business Process. It can be a Big Picture for a project in COMPANY.

Now, from the Big Picture, a new planning strategy will emerge. Which area will we take? So, it is a battle for that. It has expanded. Expansion is in Area A, B, and Area C. So, the unprofitable area might become Priority C. What becomes Priority A? Maybe because this is the direction for Profitability, we can prioritize Area A first.

We hope that the Company will be sustainable. That is, it, sir.

Interviewer

So, in your opinion, this is useful for portfolio management. Interviewee E

That is right.

Interviewer

The portfolio of the projects, sir

Interviewee E Yes

And this is useful for increasing the awareness of all stakeholders in COMPANY Indonesia. Good externally. Externally, we deal with customers. So later customer satisfaction will increase.

Trust has increased externally in terms of funding, banking, and so on. So, there's transparency in there. Internally, this increases awareness and work ethics.

My hope is like that from the point of view of Human Capital Management. Because of what you feel from Project Management, EVM, and so on, our fellows think that I have done On-Scope, but you do not know the result; how big is the Profitability? Or how small?

The hope is that this will increase awareness and further improve the work ethic or culture.

Interviewer

And a Sense of Belonging, sir

Interviewee E

Sense of belonging, right, sir? That is the future hope. Long term sustainability

Interviewer Okay, sir.

There is the last one, sir. You hope that this EBITDA integration with EVM is beneficial for the COMPANY and may be helpful for other strategic industries. Please sir,

Interviewee E That is right.

COMPANY is tier one in procuring the primary maritime dimension defense system equipment-the first tier.

The second tier is the components that support it. So, the key is not only in COMPANY but collaboration between industries.

For example, the electronics industry, the machinery industry, the steel industry, the design industry, or design manufacturers. Or other supporting industries that support ship manufacture. Interior, paint, welding, and so on

With the EBITDA method attached to EVM Project Management, we can benchmark COMPANY Indonesia's with its industry partner.

We can benchmark with strategic partners or subcontractors so the industry can excel.

The final product of the supporting industry will go to COMPANY Indonesia. Yes, the product finally went to COMPANY Indonesia because there are thousands of components that COMPANY cannot produce alone.

We hope that with the EBITDA Matrix, the EBITDA Method attached to the WBS, or project management, this will become a benchmark to be shared with other industries.

In addition, Company's strategic direction for the shipyard may be the Center of Excellence for Indonesia's shipyards, which will become an ecosystem. In this shipyard industry, COMPANY will not appear alone.

So other shipyards can follow the Company's level.

So, my hope is that way, sir, in various aspects.

Interviewer

Okay, thank you, Interviewee E

I beg permission to turn off the recording. Thank You

Interviewee E

Ready, ready, Interviewer

Interviewer Thank You

INTERVIEW Interviewee F Interviewer

OK, sir, the first questionnaire is the first question. EBITDA is the proper measure of project management profitability because EBITDA is operational profitability before payment of interest expenses, taxes, depreciation, and amortization. The EBITDA value is not affected by loan interest, taxes, depreciation, and amortization; the EBITDA value is only affected by the efficiency and productivity of project management. According to you, strongly disagree or strongly agree, or which scale is in between? Thank you.

Interviewee F Totally agree sir.

Interviewer

Good number seven, sir; thank you.

Then point two, EBITDA is the proper measure of Project Management profitability because project management and the team fully control EBITDA. And Project Management is not responsible for Interest, Tax, Depreciation, or Amortization.

Your opinion?

Interviewee F

Agree, totally agree.

Interviewer

The Third question, sir.

Companies can integrate EBITDA into earned value management by setting revenue from the authorized budget, so the authorized budget is revenue from project implementers from project management. An example of an appropriation from BMPP is a revenue plan from COMPANY. Then determine the EBITDA plan; from there, we can get a cost plan, which is the budget or revenue plan minus the EBITDA plan. Agree or not.

Interviewee F Yes, I agree.

Interviewer

What is the scale, sir?

Interviewee F Seven sirs

Interviewer

Point four. Companies can integrate EBITDA into earned value management by determining the EBITDA margin of the project, then the EBITDA margin in each work breakdown structure, and the EBITDA margin in each work Sirage so that friends in the field know whether the EBITDA margin agrees or not, sir.

Interviewee F

Agree

Interviewer

What is the scale, sir?

Interviewee F Seven sirs

Interviewer

Fifth question. Companies benefit from using EBITDA as the appropriate measure of the profitability of management projects because EBITDA is a standard measure of profitability in financial statements. If the financial report has EBITDA, if in the Project Manager, project management many indicators cannot be recorded and reported financially. Thus, company management, apart from project management, can understand the profitability of project management. In your opinion, with EBITDA in project management, the other management can understand the project's profitability.

Is the signal not good sir? Well, I was waiting for you to enter the house first.

Interviewer

Yes, yes, yes, the network was disconnected earlier! Interviewer

Yes sir.

Interviewee F

Yes, sir, you should sit down first to calm down.

Interviewee F

It was OK; it was a traffic jam.

Interviewer Ready. Ready

Interviewee F

The last one, sir

Interviewer

The company benefits from EBITDA as a measure of project management profitability. Because EBITDA measures the operating profitability of standard financial reports, many managements do not understand its profitability if the term is a term in project management. With EBITDA placed in project management, the financial statements of people in the company can understand the profitability of project management. According to you,

Interviewee F

There are two, hello?

If EBITDA is projected, I agree; scale seven, sir.

But if the corporate size is corporate profit, it is only related to EBITDA, and I disagree, so maybe it is scale six, right?

Interviewer

OK, sir, scale six, sir.

So, what we have is the project profitability of the company. Besides EBITDA, there are many other indicators, but those under project control only get to EBITDA; both points six

Companies May benefit from using EBITDA to measure project management profitability. Because project managers and teams know their targets, they can maximize project management's profitability. With the EBITDA target in the project, the Project Manager can maximize the EBITDA, and his team also maximize it. Your opinion?

Interviewee F Six sirs

Interviewer OK sir

Interviewee F

What is project management? This project management includes my job.

Interviewer

Management starts from the definition; it is not just production operations but from marketing, design, coordination, and delivery scope. So, it is not just the implementation in production, sir.

Interviewee F

Yes, but three valuable things in the project should be considered: quality, cost, and delivery. Well, this is true for boarding houses, earlier, but the quality is not yet, it is not yet massive, so if you are afraid of this, if it is EBITDA, later it will run to cost, but the quality is not, it is not recorded with the delivery.

Interviewer

So, EBITDA is under constraints, quality, safety, and delivery, so with this concept, we still maximize EBITDA; that is what, sir.

Interviewee F

This statement means that we maximize EBITDA while fulfilling the same quality. standard

Interviewer

While still fulfilling quality, delivery, and safety, standard yes.

Interviewee F

If so, seven means.

Interviewer

So instead of maximizing EBITDA, ignoring safety, quality, and delivery. The constraints are still being maximized, but we are looking for loopholes for the best profit. That is, it, sir.

Interviewer

Then the next question, sir, is to increase support from the supporting unit to project management. So, the Project Manager needs to report the actual achievement of the EBITDA and EBITDA targets to the company's supporting units so that all company management understands its profitability and can provide support.

According to you?

Interviewee F

Totally agree because that's part of it.

Interviewer Then the eighth

To make it easier for the board of directors to provide timely strategic decisions to the project manager, the Project Manager needs to report the actual achievement of the EBITDA and EBITDA targets to the BOD so that the BOD can provide direction and make strategic decisions that are beyond the Project Manager's authority. Your opinion?

Edy

Yes, six sirs.

Interviewer 6 Sir is good,

The previous question was the structured question, sir. Now, sir, please tell me about the importance of EBITDA in project management without neglecting quality, delivery, and safety.

So, in terms of quality, delivery, and safety, how important is the profitability of project management for the company?

Please, sir.

Interviewee F

So, if we see that EBITDA is one form of measurement, the measure of revenue that can be obtained because costs reduce income, then there will be profit. There will be indirect costs instead of meeting EBITDA before tax amortization, meaning when people want to go towards their destination, they must there an original note than where they are going, so this must be planned for to go to one of these achievements. The scale uses EBITDA, so this EBITDA must be controlled every month, every week, and even every day.

Following the type of project, each member must know that when he does something, it is related to the cumulative total EBITDA obtained from the project. Therefore, the most important thing is that all project members, people, or support must understand that both achieve that goal or are jointly responsible for executing the EBITDA value because this EBITDA is one of these points if the quality equals the delivery. Now that has been achieved, and this EBITDA is one of the points or goals that must be earned. Every unit must know its position and duties to accomplish the EBITDA target. The most important thing is that the

role of EBITDA will affect the success of the following process for the team or unit, then this must be monitored and controlled comprehensively every time, so the function is there on EBITDA.

It was later related to costs related to checks, depreciation, or something else that's already management's business. The focus of project management is to focus on getting evidence. The current picture is.

Interviewer

OK, sir, what are your hopes for COMPANY with its current EBITDA position, with the integration between EBITDA and earned value management in project management?

Interviewee F

Some things are broader than a company when one or two years are profitable. It can suddenly drop into a product or what is obtained from this EBITDA.

Sustainability needs management performance, so the company must be agile. To create Project Management agility, the Project Manager and the team must strengthen bottom-up system feedback, whether standard operating procedures, operational or management standards.

COMPANY already has its shipyard standards which adopt provisions or measures from abroad. This success must be supported by an integration system and measurement methods or standards required in this EBITDA. From a top-down point of view, the system is already running by default, and the target is already there. However, from a bottom-up procedure, the procedures must also be met, so consistency, application or implementation, work standards, or operational standards that are possible at COMPANY must be encouraged again.

I am afraid that when we implement the EBITDA -EVM system integration, the company rules, and regulations do not support the implementation. Management will take some time to prepare proper standard operating procedures. For example, everyone has a task card; then, the task cards are collected at the head of the workshop, where the analysis is already there.

The company needs discipline and consistency to implement the EBITDA-EVM system. The company needs EBITDA planning targets, the actual EBITDA achievements or schedules, and the system. Management must strengthen this system to support EBITDA integration.

So, the opportunity is quite ample, but it needs both top-down and bottom-up cooperation, so you must manage it together, not alone.

Interviewer

All right, Interviewee F

So, the integration between profitability with quality, safety, and delivery must be excellent. Then it must be supported by a sound system. Of course, with changes in work culture and so on. It is.

Then. Is there anything else, sir, that you need to convey about how COMPANY will achieve success with profitability, sir?

Interviewee F

It is like a life cycle in shipping; it is a life cycle or circle. So, we must start now. After one year or once or twice a year, we must evaluate our project based on our culture. The system must have been implemented, and performance management must have been measured.

Indeed, our current KPI is too general, but we do not only focus on EBITDA but also related to performance management. How we must achieve productivity and what should be done. The KPI is measured repeatedly following the existing processes in a company. Especially in the ship-building company, there must be differences between ships. Even though there are ships with the same system, we found many differences inside the vessel. So, it must be developed and then run. This ship has characteristics, high technology,

So, it must be agile towards the development of both technologies, and so our hope after this is EBITDA implementing EBITDA with IM4. With a good management process, use PMO or whatever standard is used. Do not forget that this project has good management performance related to culture because the constructive collaboration between divisions and divisions is already good. Still, it needs to be maintained and improved like that, sir.

Interviewer

Good, Interviewee F. Im sorry I will put off the recording, sir.

INTERVIEW . Interviewee G.

Interviewer

OK, thank you for taking the time to conduct this interview. We will use this interview as a case study on the implementation of EBITDA integration with earned value management in the IT SYSTEM of COMPANY In this interview, we will have two sessions-the first is structured questions. We make structured questions. If you agree with this statement, we use a scale of one to seven. Scale one strongly disagrees, if seven strongly agree, scale two disagrees, scale three slightly agrees, scale four is neutral, then five slightly agrees, scale six agrees, and scale seven strongly agrees.

OK, can I start, Mom? Can I start? Then there is the statement.

EBITDA is a proper measure of the profitability of project management because EBITDA is operational profitability before paying interest, taxes, depreciation, and amortization. Therefore, amortization depreciation tax interest does not affect EBITDA.

And the EBITDA value is only influenced by the efficiency and productivity of project management from end to end.

Interviewee G Agree on sir.

Interviewer The scales?

Interviewee G Six yes

Interviewer

Secondly, EBITDA is an appropriate measure of project management profitability because EBITDA is entirely under project management control from contract to delivery. What do you think?

Interviewee G Strongly agree, Seven.

Interviewer

Then the company can integrate EBITDA into earn value management. Firstly, setting a revenue plan, namely the Authorized Budget from the project owner, becomes the planned revenue for COMPANY. Then, the COMPANY determines the Planned EBITDA plan; so that we can calculate the planned cost of the budget minus the EBITDA plan. What is your opinion?

Interviewee G

Seven strongly agree. Interviewer

Then the company can integrate EBITDA with earned value management by setting a target EBITDA margin from the management project and distributing the EBITDA margin into each work breakdown structure and each work package.

What is your opinion?

Interviewee G Strongly agree.

Interviewer

Next is this fifth question. Companies benefit from using EBITDA as a measure of project management profitability because EBITDA is a measure of profitability in regular financial reports. Thus, people or managers who are non-project management can find out the profitability of the project management. What is your opinion?

Interviewee G Agree

Interviewer The scales?

Interviewee G Six

Interviewer

Then the company benefits from using EBITDA to measure the profitability of management projects because by determining the EBITDA of the management project, the Project Manager and his team know the EBITDA target. Thus, the project manager and his team can maximize the profitability of project management in a fixed corridor. Quality, delivery, and safety cannot be violated in this corridor. Still, they can maximize EBITDA by improving the business process in that corridor.

What is your opinion?

Interviewee G I agree, sir.

Interviewer The scales?

Interviewee G Seven

Interviewer Next,

To increase support from the supporting unit to project management, the Project Manager needs to report the achievement of the actual EBITDA with the target to the supporting team so that they know what the Project's profitability is, and then they can help. What is your opinion?

Interviewee G Seven

Interviewer

Strongly agree, madam. Then to make it easier for the board of directors to make timely strategic decisions, to project management, the project manager needs to report the actual achievement of the EBITDA and EBITDA targets to BoD so they can know the profitability of the Project can provide direction and decisions strategy which is often beyond the authority of the project manager.

For project management, the project manager needs to report the actual achievement of the EBITDA and EBITDA targets to the BoD so that he can know the profitability of the Project and can provide direction and strategic decisions, which are often outside the Project Manager's authority.

What is your opinion?

Interviewee G Strongly agree.

Interviewer

Strongly agree. OK, ma'am. Now the structured questions are done.

We are starting an unstructured interview. You are free to talk about the benefits of EBITDA integration project management. So, we learned about EBITDA and how we integrate EBITDA with EVM and IT SYSTEM. And the benefits for COMPANY in the future, please, madam, tell me.

Interviewee G

Yes sir, according to what has been explained and the training I attended by . Interviewer. The implementation of EBITDA is perfect for me, sir because we can control our shortcomings there, but we still need much effort because for the detailing of EBITDA as well as an example at COMPANY is included in direct labor, right? Including the calculation of Man-Hour at COMPANY

This COMPANY B for the calculation of PERSON-HOURS still has two variables for the measure, sir, so there are two, namely, the person hour rate according to what it is, according to the calculation, and the accounting there might include the overall costs, especially the salary and so on.

At the same time, there is also a rate that we use a standardized rate. So there's a possibility for the EBITDA calculation, sir, maybe it's necessary for that person's hourly rate, maybe if all of them later the calculation is the same, maybe for the EBITDA value it can be even more detailed because so far we have calculated the MAN-HOURS report calculation, isn't it every month, sir?

We used those two standards, so some use the standardized rate here. We use the value of 85,000.

Then we also use the rate based on the accounting calculations there. According to what Rudi said yesterday during the training, EBITDA is not affected by s amortization tax, but the accounting department

still includes the depreciation and amortization. Yes, later, if we use all of that for reporting, if we cost it, there may be many unprofitable projects, sir.

Interviewer

So, the entire company needs to be standardized first. Then with these standards, it is easier to implement in the WBS and work packages. So, ma'am

Interviewee G

Yes, we used to calculate the profitability by including the detailed rate for each hour of the machine-hour. Because so far, we have estimated that we have not used it, sir. But the exact calculation is only to calculate the person-hour for production, sir, it is like that.

Interviewer

So, the Homework is in accounting, OK, ma'am?

Standardize it first, then talk to production. If the standards already exist, entering the cost into the WBS is more straightforward.

Then the benefits of attaching EBITDA to each project management? What is your opinion?

How about integrating EBITDA with COMPANY? What are the benefits of the progress of COMPANY B? Please ma'am.

Interviewee G

Please repeat your question.

Interviewer

OK, Mom, by attaching the EBITDA to each Project so that ordinary people not involved in project management can know project profitability. This Project earned this much EBITDA, and the other Project has that much profit.

What are the benefits of that Project Profitability indicator?

Interviewee G

If it is helpful, we might know, sir, about the progress of the Project. Is the Project we control every day according to what we planned?

We can control it if we know in advance how our EBITDA is, for example, why it is small. So, we can better prevent it by making some improvements in that field.

Interviewer

Yes, when someone can control EBITDA, what is the benefit for COMPANY? Is it better or the same?

Interviewee G

Yes, I hope it is better if we already know EBITDA. So, we can be more enthusiastic about completing the work. We also can find our weaknesses so that we can fix them.

Interviewer

OK, OK, OK. So, by knowing, for example, the project's success or the project's difficulty, the team outside the project management outside the production line, such as engineering or programmers, can participate, right?

Interviewee G

It must be, sir, because without a team of engineering programmers, how can this work, sir?

Interviewer

Yes, if we know that if the production line can get support from engineering and logistics, the productivity can be higher, so yes, yes, and yes, yes.

OK, ma'am, then what do you hope for with the implementation of EBITDA with earned value management with IT SYSTEM, which is now using hand-held for every person? What is your hope the impact will be on the company?

Interviewee G

We hope and believe that COMPANY is much better, sir, COMPANY is much better.

Indeed, the company and the employee will benefit from EBITDA - EVM integration.

Interviewer

Is there any other message, madam?

We are waiting for your story, free story, madam, please.

Interviewee G

Well, I think that's enough, sir.

Later, if there is anything, I can write to . Interviewer.

Interviewer

Please allow me to turn off the recording first, madam.

Interviewee G Yes sir.

Interviewer Thank You

INTERVIEW . Interviewee H

Interviewer

Good evening, Miss Interviewee H. We begin with interviews regarding applying EBITDA in Earned Value Management in projects to COMPANY management. This question uses a Likert scale of seven. One strongly disagrees, then seven strongly agree. Among them, two disagree, three disagree, four are neutral, fifth agree, sixth agree, and seven strongly agree. I started the first questionnaire.

EBITDA is the proper measure of profitability from project management because EBITDA is operational profitability that does not include interest payments, taxes, depreciation, and amortization. And the EBITDA value is not affected by interest, taxes or taxes, depreciation, and amortization because it was calculated before that. And the value of EBITDA is only influenced by the efficiency and productivity of project management because the more efficient, the more productive project management, the better the EBITDA. Do you strongly agree or strongly disagree? Please, ma'am.

Interviewee H

I totally agree, sir.

Interviewer

Good. Scale seven, ma'am, huh? Good. The second questionnaire, EBITDA, is an appropriate measure of project management profitability because EBITDA is entirely under the control of the Project Management and Team. The project management and team are not responsible for others, interest, tax, depreciation, amortization, etc. What do you think?

Interviewee H

I agree. Strongly agree.

Interviewer

Good. Scale seven totally agrees. Third, companies can integrate EBITDA into Earned Value Management by first planning income. The revenue is planned from the authorized budget. So, the budget from Airbus from Boeing is the Company's revenue plan. Then to get a cost plan, EBITDA must first reduce it. EBITDA margin, EBITDA.So revenue minus EBITDA is where we get the cost plan. So, we must set aside EBITDA, so the costs do not exceed that target. What do you think, ma'am?

Interviewee H

I agree, sir, so that we can control the budget. So, I totally agree that we can know the profits later by separating the EBITDA.

Interviewer

Yes, Thank you, ma'am. Now we enter the Scale is seven. Yes, I totally agree. Then questionnaire number four. Companies can integrate EBITDA into Earned Value management by setting the target EBITDA margin above the project; each WBS also includes its EBITDA margin. So, each WBS has an EBITDA margin; there is a target. There is also a Work Package so these people can know their responsibilities. What do you think?

Interviewee H

Yes, I agree. I agree there. So, we can know the control. I do agree, sir. Strongly agree. So, in each Work Package, there must be an EBITDA target. So, we will know later how much the actual profit is there. So, from the above, we already know we will manage projects in each WBS.

Interviewer

OkayOkay, mom. Thank You. Number four is a Scale of Seven; you totally agree-fifth questionnaire. Companies can benefit from using EBITDA as a measure of project management profitability because EBITDA is a standard operating measure found in financial reports. Because if EVM only uses its indicators without formal financial statements, it cannot display the profitability indicator that ordinary management people easily understand. So, with EBITDA in project management, company management can determine the project's profitability. So, in project management, EBITDA is attached so that financial and legal people (for example) know the profitability. What do you think?

Interviewee H

Yes, everyone should know, sir. So totally agree.

Interviewer

Now the next is to increase company support so that management can determine if the project manager reports actual EBITDA and targets. So, there are actual EBITDA and targets so that supporting units know their profitability. In your opinion, do you agree or disagree?

Interviewee H I agree, sir.

Interviewer

Good. Next, the next question is to make it easier for the Board of Directors to provide strategic and timely decisions to the project manager; the project manager needs to report the actual EBITDA and EBITDA targets so that the Board of Directors can give direction or provide strategic decisions that are beyond the project's authority. Management. What do you think?

Interviewee H

Yes, it must. Must report, sir.

Interviewer

Okay, okay, okay. This last question is the structured interview, and it is done. Now, please tell me your opinion about the importance of EBITDA to measure project management profitability so that the manager knows and can control it. Other managers who are not on the project also know. Please, Madam, tell me freely, tell me.

Interviewee H

Yes. So in my opinion, because this is also new, yes, and this actually turns out to be accurate as it is, so far we don't know because we work, don't understand how much our income is, with . Interviewer's knowledge about EBITDA, we have to know that the So far, we are, if I may be honest, yes, it was wrong

because we referenced all Costs of Production (COP). So it should be, okay, okay, COP, but we determine first how much we want the margin so that the EBITDA later we can calculate because the margin so far, we have not been involved in defining it.

In sales, only sales, and in that case, what percentage of EBITDA can be determined? For example, we set a benchmark of 25% so that our costs will not exceed what we have set, sir. So, we know how much profit there is so that people also see whether we can measure whether our project is actual or not. And everyone can tell, I see. So, measurably, and openly, everyone knows whether every project is profitable. That is how it is. It does matter that EBITDA.

Interviewer

Yes. Okay, okay, okay. So, do you have anything else to add? The benefits for the project management itself?

Interviewee H

Suppose the benefits are for project management so the management can know, sir. Can measure whether the project has exceeded the budget or ceiling of what is there. So, if we do not know the (target) EBITDA, we will use the cost, even though it will be a loss, right? So, project management can control whether it is already over budget or not, from what has been used, so from the ceiling, from the budget ceiling. So, he can,

Interviewer

What are the benefits for financial people and marketing people with EBITDA in project management?

Interviewee H

As for accounting and management people, from the data from EBITDA, he also knows that to support the project, he adjusts the budget allocation so that the EBITDA target can be achieved, sir. So, for financial people, he must look at each project's EBITDA target and how much they must allocate for the supporting project so that the project can reach the EBITDA that has been targeted. So, people,

Interviewer

For marketing people, what is the point if there is EBITDA in the project?

Interviewee H

For marketing people, he will know at the time of selling, sir. We have already set this EBITDA, so he has a limit. I will have to sell on top of that, plus the profit margin, which is up to the company. So, that's a company decision, but it should be determined, as . Interviewer said, if it can be above 20% because, with current conditions, it's only a 10% profit margin plus General &Administrations, which is 11%, that's after accumulation and all sorts of actual how many years has EBITDA been in the past? It is far away, sir, downstairs. So, a marketing person must know how much EBITDA has been determined by the project so that he can sell to, sir, his customers.

Interviewer

All right, then, Madam, um., do you have any more suggestions? How is EBITDA implemented in the project? What are your expectations for the company?

Interviewee H

So in my opinion, before..., because the base of this project is KB, right, Sir, Business Studies, it's better when you make a Business Study, don't decide directly by sales, marketing, they have to participate first, we project people also have to know, everyone, unlike the existing conditions, we must also be involved so that we also know whether what is decided later in the business study can be implemented, that is. So, if you think it cannot be done in a business study, then there is no need, which is it. So (except) if the project is called prestige, that's different again, right, sir, yes. But there must be calculations that we have to make a profit like that, sir.

Interviewer

Yes, yes. Okay, okay, okay, okay. Yes, mom. There are more. Is there anything else you want to say?

Interviewee H

I think that's enough, sir. So, if, for example, there might be this one later, I might be able to provide information to . Interviewer for that update, and at the same time, I also know the materials that . Interviewer sent me. That is new knowledge for us here, sir. We thank you for sharing your expertise in the middle of the night. As soon as you get the information, you immediately enter the group. So, we too, oh what, here? . Interviewer is still reading at this hour, I see. So, we sometimes feel like yesterday at dawn, . Interviewer, we already have new knowledge that journals for project management are like that, you know, . Interviewer.

So, it was combined (in our project), so it is for learning like that, sir. It was understanding that you still want to read that way even at that age. I still want to take it apart; that is rough, sir. So, it is a good sharing of knowledge; that is what I asked yesterday, if you can, please share the knowledge that . Interviewer gave to the operator but in that language, sir, because to be honest, . Interviewer's language is a high-level language, right, sir? Because you are different, right? For going to the operator, it will not hit, sir. I am still confused; yes, sir. So, I have tried to socialize with the operator's friends with EBITDA. Why are you late? Why are you like this? Yes, sir, Madam, like this, because Pak Luqman is like this, just like what Pak Interviewer called Pak Luqman at that time. And it exists, so. That is why I want . He to inform everyone how the Scales do not know how to make the Scales easy, so my fellow professionals, oh, this you know, as Interviewer said, even in Ferry Company, the Security Guard feels grateful love. Oh, I was able to increase EBITDA. Well, that is not it, sir. That is why I told you yesterday, right? Sir, this, this. And you have called Pak Luqman that way. Well, that is also possible so that our fellow professionals are aware and continue to understand that this, you know, is the function of EBITDA. Continue to be linked again with facilities and others. Like that, sir. So, I think like that. So, because, to be honest, we are the only ones in the group that you constantly update, isn't that, right? If our fellow professionals do not update their knowledge, sir. I do not understand what EBITDA is. I do not know where this is going. That is, it. If I tell the story, who am I? That is why yesterday I said . Sir, if I were to change this, I cannot. That is why (must) go through you.

That is why it was escalated until it was finally found that Spirit was declared a loss, and, in the end, I had surgery, and I made a presentation of it so that he reached the Director , just like what I had told . Interviewer at that time. This one function is too high (cost). But who evaluates herein? Well, I am just

saying because I do not have authorization there. But I know which areas we need to re-evaluate; that is, it. Well, the Director yesterday asked for clocking, sir. So that was also the case; it has to do with EBITDA. Between the plan cost and the actual cost so that it can be balanced, sir. That is . Interviewer.

Interviewer

It is fantastic, it is incredible. I also hope that the COMPANY will be victorious by using this knowledge and providing us with prosperity. It is. Thank You.

INTERVIEW . Interviewee I

Interviewer

All right, . Interviewee I, good morning. Let us start the interview. There is a questionnaire on a scale from one to seven, and one scale strongly disagrees. The scale of seven strongly agrees, so you need to state the scale of this statement. Let us start with statement one, Sir.

Interviewee I Ready

Interviewer

EBITDA is a proper measure of project management profitability. EBITDA is operational profitability before paying interest, taxes, depreciation, and amortization.

EBITDA's value is not affected by tax interest and depreciation because of the way it is calculated. In addition, the EBITDA value is only affected by production efficiency and project productivity. EBITDA will be good if the project is efficient and productive. If the efficiency and productivity are bad, then the EBITDA is bad.

According to you, 1 to 7 or a value of 1 strongly disagree, up to seven strongly agree.

Interviewee I

Yes, for this point, I answered strongly agree, Sir, point seven, yes. Because indeed, when talking about EBITDA, as you said earlier, measuring profitability means efficiency. There are various kinds of efficiency. Yes, Sir, it means efficiency from the point of view of things that are not needed and not necessary, then efficiency from operation, and so on. The other side is that the acceleration of production, as you said earlier, is aimed at achieving higher EBITDA than before, amortization, etc.

Interviewer

The second question, EBITDA, is the proper measure of project manager profitability because EBITDA is under the control of the Project Manager and the team. Meanwhile, interest and taxes are beyond the project manager's and team's control. What do you think, Sir?

Interviewee I

I answered yes, I have six, Sir. Yes, I agree, Sir. Six is close to seven. Why did I answer six? Because I agree that this project controls the overall operations, but indeed in our organization, it is not entirely solely under PM's control. So, some are not under the control of the Project Manager and team. For example, material procurement is not under the control of the project management team, but

INTERVIEW . Interviewee I other

divisions control it, so we do have to coordinate with them. Even though we manage and evaluate, we sometimes depend on this condition, Sir. So, answer point six, Sir.

Interviewer

OK, Sir, thank you, Sir. Let us go to number 3. Companies can integrate EBITDA into earn value management by setting an income plan from the budget. We determine the EBITDA from that budget first to find the project's costs. So, the project costs do not consume the whole budget, but we secure EBITDA. What do you think? Do you agree or disagree?

Interviewee I

Strongly agree because, indeed, as the basis of the project, we must determine the EBITDA first. The costs will be defined later. And we will also implement this in BMPP 2 following the terms and conditions directed. Indeed, the implementation is like that, sir, then determines EBITDA, margin, and others.

Interviewer

OK, Sir. Then question number 4, companies can integrate EBITDA into earn value management by setting the EBITDA margin for each WBS and Work Packages in each WBS; Work Packages have an EBITDA target so that friends can see and control it. Do you agree or disagree?

Interviewee I

Strongly agree, Sir. Yes, I agree. Scale Seven, yes, because it is in our project too. We do that. Meaning we make a schedule and other things. We also make the WBS to determine the EBITDA margin. Then the Work Packages are also more detailed. The better, the more precise the monitor, the more straightforward it is, Sir, so my point is just point seven.

Interviewer

OK, Sir. Then point five.

Companies benefit from using EBITDA as a measure of project profitability because EBITDA is a standard measure of the profitability of financial statements. EBITDA is in the financial statements.

If there is no EBITDA in the project, people outside the project do not know the project management.

profitability. So, with EBITDA in project management, everyone can know and understand the project's profitability.

What do you think?

Interviewee I

We are number 6, Sir. So why agree and number 6?

Because there is a little gap, right? I do agree with that statement. However, it is challenging to convey what you described earlier regarding implementation. Sir, it is tough to provide what information is,

all of which are difficult to explain.

Interviewer

OK, Sir, question number 7 is to increase support from supporting units from logistics to engineering to project management, so the project manager needs to convey the actual EBITDA of the target to the supporting team. Hence, he understands that if there are difficulties, they can help. Do you agree or not?

Interviewee I

Absolutely agree, Sir. Because you have conveyed this many times, not all information like this is understood by the people in the field. Only so many % of people understand the plan, and 5% know the targets according to KAPLAN if I am not mistaken.

That is so this is mandatory, and not only EBITDA, including the Annual Budget, should be informed to the people, including all the targets must also be conveyed to the people, Sir.

Interviewer

Then the last question is to make it easier for the board of directors to make the right strategic decisions for the project manager; the project management needs to report actual EBITDA and EBITDA targets to the Board of Directors so that the Board of Directors can make timely strategic decisions beyond the project manager's responsibility sir.

Interviewee I

Yes, I agree, . Interviewer, because it does not rule out the possibility that operations within the project depend on many parameters, for example, market selection, for instance, during the program, when buying something. At first, we planned this, but we could not control some conditions. For example, if there is such a condition, a war, and other things, it could be an increase from the initial planning. If that happens, we must report to the board of directors so that the BOD can decide following the changing condition to maximize EBITDA.

Interviewer

OK, Sir, this is exceptionally good. The structured interview was done. So now, kindly tell me your opinion, a free story, how vital is EBITDA to help you improve project performance? Please give your idea freely, Sir.

Interviewee I

OK, . Interviewer, in the project I managed before this BMPP (Barge Mounted Power Plant), we did not

all of which are difficult to explain. use EBITDA terminology, right?

But there are control functions that we did, too, so this project started with planning, Sir.

With good planning, there will be good monitoring later, producing sound output, especially for us. We also discussed the profitability problem, quality issues, and so on.

Regarding the EBITDA, this is necessary. But indeed, in a place like what I said earlier in the questionnaire, not all activities that are within the authority are not fully authorized by the project management team.

Indeed, sometimes there is that area in project management.

The most dominant factor of project management success is not in the construction services area or anything. But the success factor depends on many supporting units such as purchasing area, yes supporting, or in we talk about the supply of materials. For example, if the company cannot control the materials supply, the value is almost 60% itself, Sir. On the other hand, we are in the production department and want to monitor improvement, but we only control 30% of the value of the project.

Precisely, yes, if we also control the key, we, in all units, must have the same understanding of EBITDA. That is why you also said earlier that there is a need for outreach to all parties related to this EBITDA system so that they fully understand the EBITDA targets if all systems hold them.

We may start the EBITDA awareness from engineers in the engineering department, people in the supply chain, production, and so on, up to product delivery. If they have targets in every work area, they can achieve their EBITDA target, Sir.

But there are specific departments; as I mentioned earlier, the supply chain that business process impacts EBITDA results significantly.

Why? Because the supply chain process is a bit out of control, Sir.

So, the EBITDA control cannot be 100% at our production department. We (the production department) only coordinate preparing budget allocations. We evaluate, and so on. But material supply delays often happen that exceed the initial planning schedule, . Interviewer, which will surely erode the EBITDA margin that we set.

That is also an obstacle. That is why every unit needs to focus on the EBITDA, and all business lines must understand their goals and EBITDA targets.

Then how to achieve EBITDA on each business line. So, EBITDA is not only 100% of the project management (PM) responsibility on site (Production) but other supporting units' responsibility.

Yes, so if the supporting unit does not know the impact, yes, Sir, it cannot support Project Management properly.

Interviewer

So, if the supporting unit does not know the impact of their job on EBITDA results and they do not understand the project profitability, they work without caring about EBITDA results. Sir, they cannot

support the project

Interviewee I

But the impact on the result is that we face difficulty controlling the project and its outcome. Overall, it seems that the Project Manager and others are incompetent. This underachievement is the problem-the problem of many connected systems.

So, finances are also affected, then production is concerned. And financial and material support to the production line becomes unreliable.

Yet, in the end, the production line must execute the jobs even though it is late. We must catch up to finish the project.

As you said earlier, maintenance was late in the case of Merpati Airlines. However, some aircraft maintenance was late for r Boeing aircraft. In the end, we must make the delivery.

Interviewer

So, everyone needs to know the project's profitability to participate. Is that correct, Sir?

Interviewee I

Yes, yes, at least. Even if people do not know the complete result, they should see the target in their line. It is excellent if they know the whole thing, but at least they understand the impact of their job on the project.

Interviewer

OK, Sir. This is good.

As we record the interview, now let me turn the recording off.

# INTERVIEW . INTERVIEWEE J

Interviewer

Good evening, . Interviewee J

Interviewee J Evening sir

Interviewer

We begin the interview by writing a case study at COMPANY regarding the application of EBITDA in Earned Value Management in project management; COMPANY is a project-based company or project-based corporation; therefore, to increase Company's EBITDA, EBITDA is raised from project management.

We will conduct this interview to determine the management's understanding of implementing EBITDA.

.

COMPANY manages project management, then Earned Value Management is earned value implemented by COMPANY. Then the Work Breakdown Structure is the WBS implemented in Company's Earned Value Management.

The work package is a work order to implement the Work Breakdown Structure. Then EBITDA, or Earnings before interest, tax, depreciation, and amortization, is income or profit before paying interest {interest}, taxes, depreciation, and amortization. And EBITDA margin is the percentage of EBITDA divided by revenue. The question is twofold. One is structured questions; there are eight questions. After that, you told me about the implementation of EBITDA in earned value management at COMPANY and your expectations with this implementation.

First, we use a Likert scale, 7 on a Likert scale. +1 strongly disagrees. 7 totally agree. If number 2 disagrees, 3 somewhat disagrees, 4 is neutral, 5 somewhat agrees, 6 agrees, 7 strongly agrees. Shall we begin, Sir?

Interviewee J Yes Sir

Interviewer

EBITDA is a proper measure of project management profitability Because EBITDA is operational profitability before paying interest, taxes, depreciation, and amortization. And the value of EBITDA is not affected by interest, taxes, depreciation, or amortization. The EBITDA value is only affected by the efficiency and productivity of project management. Your opinion Strongly disagree or strongly agree or some of them?

Interviewee J

The program manager can control it because it is an EBITDA issue. So, I totally agree with that, **right?**

Interviewee J

OK, Sir. All right, Sir, Number 7, Sir, **right**?

Interviewer Number 7.

Interviewee J

The program manager can control it because it is an EBITDA issue. So, I totally agree with that, **right?**

Interviewer

OK, Sir. All right, Sir, Number 7, Sir, **right**? Number 7.

Interviewee J Scale 7

Interviewer

The second question, EBITDA, is an appropriate measure of project management profitability Because EBITDA is entirely under the control of the project manager and team. Your opinion Do you agree or disagree?

Interviewee J

Seven again, Sir. Because it has something to do with number 1 earlier

Interviewer

OK, Sir. Then this is number 7.

The third question, companies can integrate EBITDA into earned value management-the first step is determining planned revenue. The revenue comes from the authorized budget. So, if we get a project from Airbus, the budget Airbus provides planned revenue or revenue plan. Then to get a cost plan, first determine the Plan EBITDA and reduce Plan Revenue by EBITDA, say 20%, 15%, or 25%. For the rest, we make a cost plan. In your opinion, do you agree or disagree? So, the planned costs are the Planned EBITDA reduces planned revenue. Then for us, the rest is the cost that we can consume.

Interviewee J

Mmmmm. Earlier with the EVM, yes, Sir, it was reduced from there.

Interviewer Yes, budgets.

Interviewee J

It means budget management.

Interviewer

The budget is not a cost, but the budget from Airbus is the Company's income. Then what can be spent is not the budget, but we must set aside EBITDA first. After st aside the EBITDA from the revenue, the rest is the planned cost that we can only be used for spending.

Interviewee J

Yes, yes, I agree, Sir.

Interviewer

Fine, Fine, agreed.

J unit

That is what it is used for.

Interviewer

Then. Companies can integrate EBITDA into earned value management by setting the EBITDA margin from project management. Then this project management EBITDA margin is distributed to the EBITDA margin in every WBS, and the EBITDA margin percentage is 20% up to the workback instructor. Do you agree or disagree, Sir?

Interviewee J Agree.

Interviewer Agree fine.

J unit Agree.

Interviewer

Number 7, Sir. Then the fifth. Companies benefit from using EBITDA as a measure of the profitability of project management because EBITDA is recognized in financial statements. So, with the EBITDA indicator in project management, Company's management can know how much profit it is. Because if you only use earned value management, many financial people do not know production terms, marketing people do not know, and logistics people do not. What do you think, Sir?

Interviewee J

Yes, if it is included in EVM, I agree with using EBITDA.

Interviewer

OK, Sir. Good. Then the Sixth, companies can benefit from using EBITDA to measure project management profitability. Because then the project manager and his team know the target. Know the EBITDA target and understand the profit target so that he can maximize profitability by improving the process basis. Your opinion?

Interviewee J

Yes, I agree. Strongly agree.

Interviewer

OK, Sir. Seventh, to increase support from supporting units, engineering, logistics, and procurement, the project manager must report its actual EBITDA achievement compared to its target. The supporting team know and can help better. Your opinion?

Interviewee J

Agree. Strongly agree. So that the support can

Interviewer

Yes Then, point 8, to make it easier for the directors, the board of directors makes timely strategic decisions, so the project management needs to also report to the BOD what the actual EBITDA is and what the target is so that he can anticipate making strategic decisions that are sometimes not owned by the project manager because the project manager's decisions are limited if the directors know because they can make timely strategic decisions. What do you think, Sir?

Interviewee J

They can decide, Sir, for BOD based on the EBITDA daily report. Give directions so that it really agrees.

Interviewer

OK Yes. You have completed structured questions, Sir. Now all that is left is your story, what your hope is, what you think about the integration of EBITDA that is attached to value management, then each project is found out about its EBITDA, what do you hope this integration will do for your company, Sir? Please.

Interviewee J

It is OK; it is a bit long, Sir, isn't it?

Interviewer

Yes, Sir, it is a bit long. Yes

Interviewee J

Like this, Sir, so EBITDA, frankly, yes, it is a new term. I have been engineering all this time. So, all this time, you have thought that EBITDA is a financial matter like that, Sir.

Interviewer

Yes, that is right.

Interviewee J

Well, yesterday, it was introduced like that, and it has started. What is more, by reading the chairs' names, I started looking for them too, Sir. I have often asked . Interviewer since we are in engineering, we only think about the design process to produce a product and the product development process. We have never thought about EBITDA in our daily activities.

While we may develop one product in five or seven years, we can usually improve the business process from several interactions and discussions with production planning and control. We can at least take one step to improve the business process efficiency and effectiveness to deliver the product on time. We can contribute to enhancing the EBITDA.

Since engineering is a cost center, its EBITDA value is usually negative. But we can improve the business process to improve the value of EBITDA.

So, by understanding EBITDA, engineering people can be creative to seek the proper business process to improve EBITDA. They can enhance the business process and create innovation that produces better revenue for the company. Creative and innovative engineering design for the product and business process may improve the EBITDA.

Interviewer True, true

Interviewee J

I can illustrate that because you said that EBITDA is earnings earlier. For instance, the taxi driver does not just sit idly to get profits from morning till the afternoon, but he can find another place or, on the way, to get passengers on the road who can get on.

Interviewer

Yes, and, it is not only the person who produces the revenue directly who is responsible for EBITDA, but also the supporting team is responsible for improving EBITDA. If we are public transportation drivers, we are taxi drivers; that is our responsibility.

Previously, because the term EBITDA is in English and financial terminology, we thought it belonged to finance people. We did not dare to touch EBITDA.

Physicians and Surgical doctors rarely dare to touch EBITDA. But once they look at the EBITDA structure, it is income before tax, depreciation, amortization, and interest. They will understand that EBITDA is their responsibility. I also realized that several years ago, I told people that EBITDA was the company operations' responsibility. When the operation team understood that EBITDA is their responsibility, they felt a sense of belonging and commitment to deliver the best.

Then what do you hope for in an atmosphere like this at COMPANY? How can you participate in increasing the company's EBITDA with this knowledge, Sir? Please, Sir.

Interviewee J

Yes. I hope this is true, Sir; I will look at the background first; yes, I joined COMPANY in 1993, at which time I did not blame the situation. Because indeed, engineers who join the company are asked to learn and absorb knowledge because the company motto at that time was: "Starts from the end and ends at the beginning."

Indeed, the task of engineers is to focus on learning. Well, the thing that has carried over from its engineers is a character like that. We must break that mindset to be more open-minded, Sir.

So, I am often in the technology and design center division; that is me; frankly, many engineers in my batch or those who are more senior; still have closed-minded characteristics. They do not want to learn the other knowledge that engineering. That is what needs to be examined because so far, if there is, for example, there is something called a case, it is even more debated.

So, I remember when I was at GPM floor 9, during the meeting with all the directors there, I asked the same question to you. And you suggest that the engineering department be assumed as a single company. And the engineers who run it should have entrepreneurship discipline.

Interviewer That is right.

Interviewee J

What if there is a request for something? Masa, I said I cannot because this does not exist, right? Not one company.

I mean, the engineers must be aware of and understand the costs and revenue sides. So, we see the costs of the business process and the potential revenue that the outcome or product may generate.

We should know how it works, right? If we know one side, we should know the other side.

Second, we should innovate and create something unique or more advanced. When we make a product, we should be silent so people can only notice when we finish production and produce an excellent product.

Interviewer

And the measure is money. Indeed, EBITDA money is constrained by delivery, quality, and safety corridor. The corridor remained unchanged. But we are improving efficient and productive business processes in that corridor to earn more money. Well, that is about it.

This system of maximizing EBITDA will bring up entrepreneurship. So, we can sider every person not as an employee, so he is a leader and will be able to lead like that. OK, here is another addition, Sir. What do you think about implementing EBITDA in project management for COMPANY?

Interviewee J

Yes. I hope the engineers must know the engineering knowledge to design saleable products and the ability to maximize EBITDA. The point is that we should develop engineering competencies such as testing, etc. So far, we have only focused on supporting COMPANY products-for example, the interior, dor the new orders, changing interior cabins, etc.

But if we have a bus like that, we can look for it in other places.

Interviewer Entrepreneurship, Sir

Interviewee J

We use this existing potential to serve COMPANY products and outside of the many airlines' private jets, whose interiors may have to be replaced periodically. Well, if they are made aware of these thoughts from the EBITDA side, people can open their minds, Sir. Engineers, it turns out that our activities will impact when we think about it more creatively, not just focusing on what we have been doing routinely, like that.

Interviewer

I am so glad you have come a long way from thinking about it. Yes, I hope other COMPANY fellows can follow you like that. OK, Sir, your message to COMPANY, fellows, seniors, or juniors about how to use this EBITDA to improve COMPANY. Please, Sir?

Interviewee J

Yes. Come on, people, both senior and junior, let us realize that we already know about aircraft science at this time. We have mastered it, but how to make this knowledge into a product that sells well and makes money? We must understand EBITDA first so that the profitability of our activities in design can increase. That is what we all need to realize for the Company It is Sir.

Interviewer

OK, Sir. I will close the recording first, Sir. I am asking for permission. Let me turn off the recording for a moment, Sir.

INTERVIEW . Interviewee K

Interviewer

Good morning, . Interviewee K.

Interviewee K Goof morning

Interviewer

So, we started the interview for the case earlier at COMPANY regarding implementing EBITDA at earned value management. The explanation is that project management is project management managed by COMPANY. Then earned value management is a system that is implemented for project management. Then the WBS or work breakdown structure is applied for project management with EBITDA. Then work packages are work orders used to conduct work breakdown structures. Then EBITDA is earnings before interest tax depreciation, amortizations are income, profits before payment of interest expense or tax, then depreciation and amortization. Amortization is depreciation in kind, not physical like permits and so on. Then the EBITDA margin is EBITDA divided by income; now, in the question later, it was stated that we would use seven Likert scales. The scale of one strongly disagrees; the scale of seven strongly agrees; then number two disagrees; number three somewhat disagrees; number four is neutral; number five somewhat agrees. The scale of six agrees, and the scale of seven strongly agrees. I have a question. First, . Interviewee K, I have a question.

Interviewee K

In the meantime, it is clear enough, . Interviewer. Thank you.

Interviewer

OK, let us start with the first question: EBITDA is the proper measure of project management profitability because EBITDA is operating profitability before payment of interest expense, taxes, depreciation, and amortization. The previous amortization, depreciation, tax, and interest rate unaffected the EBITDA value. Then the EBITDA value is only influenced by the efficiency and productivity of project management; your answer is neither agree nor disagree.

Interviewee K

I agree, sir. It is just that for this answer, I did not choose the number seven but preferred a scale of six, . Interviewer.

Interviewer

OK, what is the explanation, sir? Interviewee K

All right, sir, this is related explicitly to COMPANY; Company's products are unique. So every time we deliver an aircraft product, there are three project stages, sir; this project is divided into three; the first is engineering to order, so there are products that we really have to design first, and some we manage based on make-to-order, so then what's the name, the project or the contract is effectively signed, we just made it now the third one is made to stock. Hence, this is the stock level that we manage so that the service level for product delivery is more on time, so all that remains is assembly and then testing and shipping.

Now, out of these three, in fact, COMPANY, sometimes there are programs that engineering-to- order do not enter the contract, so what is the name? Effective efficiency is not efficient, yes, the distribution of the burden, for example, as an example yesterday, to change the propeller from a DOUTI propeller to an MT propeller, so what is the cost for what is the name for the development cannot be charged to the project directly because the customer does not pay for the product development. The customer knows the product is already good, so there is no need for additional development.

It needs to go into the invisible project, but the cost must be controlled, sir, so is it the next ten aircraft, or how many are the next aircraft? Following the investment study for what is called maintaining the sustainability of its products at COMPANY, it is him, but that does not include interest tax depreciation and amortization.

So, this is what I do not know, so what I understand is, sir, I would like to clarify this, if the expenses for depreciation and amortization are not included in the calculation of EBITDA, the project might be unprofitable. In this case, the project management will be unprofitable. So, I think depreciation and amortization should be included in calculating project management or product costs.

Interviewer

What is under the control of project management does not include depreciation and amortization. Because it is under the control of the board of directors, right?

Interviewee K True, true.

Interviewer

Yes, we are talking about the project management scope of responsibility only, which does not include the responsibility scope of the directors. In this case, the interest, taxes, depreciation, and amortization or ITDA are not the responsibility of the project management. The company must earn sufficient EBITDA to pay the expenses for Interest, Tax, Depreciation, and Amortization or ITDA.

EBITDA itself omits ITDA. We must reach a higher EBITDA than ITDA to pay it-yes, yes, interest, tax, depreciation, and amortization. The project management cannot increase or decrease depreciation, interest, and taxes either.

So, we are only talking about those under Project Management's control. The Project Management scope includes design, marketing, and delivery, which is not affected by depreciation variations. So, if we are diligent, the business process will be efficient, and EBITDA will be reasonable. But if we are lazy, the business process is inefficient the EBITDA will be insufficient. EBITDA result depends on the business process efficiency and productivity but does not vary due to the variation of ITDA.

Interviewee K

OK, thank you, Interviewer; please allow me to have a phone call; please pick up the phone for a moment.

Interviewer

OK, I will post it first, sir.

Interviewee K Ready, thanks.

Interviewer

OK, Interviewee K, which is the first question earlier. Your answer is like six.

Then the second, EBITDA, is an appropriate measure of project management profitability because EBITDA is entirely under the control of the project manager and the team, from engineering requirements to delivery. Do you agree or disagree?

Interviewee K

I agree with this statement that EBITDA is a proper measure of project management profitability, as it is under the project manager's and team's control. It's just that I'm here with the condition that I'm currently in sixth position; the reason is that, for now, the project organization at us is COMPANY, indeed, the authority of project management is still limited, so from yesterday's directors, one of them appreciated this project management so that the control can be maximized, sir. DI is not optimal yet, then Interviewer.

Interviewer

Yes, indeed, finance cannot control project management because project management is under the control of the team and project. OK, sir, number six, OK, then, point 3, the company can integrate EBITDA into earned value management and determine the income from that income, the authorized budget; if we look at it from the arrow structure, the budget arrow structure is the income of COMPANY, then to get the cost, we first determine the EBITDA target, the EBITDA plan from the revenue plan. Then there is an EBITDA plan there; then, we get a cost plan for a project management cost plan. So, the project manager's cost plan is not to spend all revenue. We do not set aside margins or EBITDA first; only then can we establish a cost target. In your opinion, do you agree or disagree?

Interviewee K

OK, sir, for this statement, I agree that we can integrate EBITDA into EVM by setting a revenue plan. Precisely that is what is essential in COMPANY from the authorized budget and determine the EBITDA plan to resolve this management fee. I agree. For the value, I chose number seven, Interviewer.

Interviewer

OK, Interviewee K, number seven, for number three. Then the company can integrate EBITDA into EVM. By setting a target EBITDA margin for the project, the EBITDA margin is also set in the breakdown structure and each work package so that everyone knows the target and can work according to the target through this integration; what do you think?

Interviewee K

OK, for statement number four. Those companies can integrate EBITDA into EVM and set margin targets for each WBS; I agree. And for this, I choose number seven. Totally agree.

Interviewee K

Yes, yes, at least. If not from that line, the people do not know the whole thing, but at least he knows the target in their responsibility. Thankfully, if they know the entire strategy or planning, at least if he understands and understands their duty, it will impact the whole project.

Interviewer

OK, sir. The interview is recorded, and I promise to turn it on first.

Interviewer

Then question number five. Companies can benefit from using EBITDA as a measure of project management profitability because EBITDA is a standard measure of profitability for financial reports, so if there is not any and the profitability is in project management, management wonders how much profitability this is. So, management with its EBITDA as an indication of this, company management can understand the profitability of each project management. So, it is not the scales that appear, not the scales for operations regarding quality, but the Financial Measure can appear. So, the company can benefit. What do you think, sir?

Interviewee K

OK, sir, for this statement, the benefits of EBITDA as a measure of project management profitability for standard operating measures, I agree with this statement.

Interviewer

On what scale, sir? The sound is gone sir; the signal is not good.

Interviewee K break up, yes.

Interviewer

How about scales?

Interviewee K Scale seven sir

Interviewer

Then point six, companies can benefit from using EBITDA as a measure of project management profitability because then the Project Manager and his team know their EBITDA target; by understanding the target, they can maximize the Project Manager's profitability by improving business processes. What do you think, sir?

Interviewee K

Yes, Interviewer, for this statement, what are the benefits of EBITDA as a measure of profitability? It needs to be known for all Project Managers and teams to achieve their targets; I totally agree, sir, and I choose this on a scale of seven.

Interviewer

OK, sir, OK. Then next is the question of increasing support from the supporting unit from HR from accounting to production strategy planning to project management; the Project Manager needs to report the achievement of EBITDA, actual compared to the target so that everyone is aware of the condition of the project. Do you agree or disagree?

Interviewee K

OK, thank you, for the statement that information, this EBITDA target needs to be known and achieved by all supporting units; I totally agree that the supporting units can also help appropriately following the EBITDA conditions, and I choose scale seven, sir, for this one.

Interviewer

Then the next one is the last question for a structured question. Later, after this, I want you to tell a free story. There is no structure; how is the EBITDA application in EVM at the company, for example, number eight, to make it easier for the board of directors to provide direction or provide timely strategic decision support to the Project Manager, the Project Manager needs to report actual conditions compared to the target, so the board of directors can make timely strategic decisions, which decisions may often be beyond the authority of the Project Manager. Your answer?

Interviewee K

Thank you, Interviewer, for the point of this statement that the actual EBITDA and EBITDA targets need to be known by BOD to make decisions beyond project management responsibility. I strongly agree. This responsibility is also related to the authority of the project management question above, and I gave it a scale of seven, sir.

Interviewer

OK, Interviewee K, the structured questions have been completed; now, sir, please give your opinion on how the company can use the integration of EBITDA into Earned Value Management. DI in increasing EBITDA considering COMPANY is an aircraft manufacturer whose projects have tens of thousands of work packages. If the project is not appropriately structured, what if the project is structured? How can you control it? Kindly speak freely.

Interviewee K

Thank you, Interviewer, regarding the integration of this project management with EBITDA and EVM; maybe I'll tell you about my experience as a Project Manager, sir, so in 2015 I was appointed Project Manager for Bell helicopters, especially handling components where this project is a derivative of offset the purchase of a significant number of 39 helicopters from the Indonesian Army through COMPANY to the helicopter Bell. In this project, I tried to ensure that the project's profitability was well maintained, sir. So, at the beginning of the project, the challenge was that we transferred some of the existing production capabilities in Poland to Indonesia. So there, it is already calculated that each activity has a target cost and a timeline for what it is called the implementation for each so that this target case is achieved. So here I will tell you that two obstacles may be relevant to EVM. The first was that following the contract, there was no shipment of tools from Poland to Indonesia, while the delivery targets had to be maintained. So, what efforts do we need to make tools that do not exist, sir? There is no WBS. Because we deliver this, we still must make it; why are we pursuing the first delivery for the second component, indeed the Ministry of Defense are awaiting this INDUSTRIAL DOMESTIC CONTENT offset because it is included in Defense Requirement; this must be carried out. So, in the end, to be able to take good care of the project target, it becomes the EBITDA project, so I negotiated with Bell, sir, OK, we will implement these changes, changes related to this WBS with the addition of making tools, but you have to pay for that, so the point is, even if you don't it can be maximized because we also maintain the profitability target, yes, so if we all overdraw the tools, so we prioritize delivery, so we prioritize which shipments are the priority from start to finish, those that cannot be delivered, that's all we make. Well, finally, now with the process.

Interviewer

You have been thinking about the profit margin but do not know the term EBITDA.

Interviewee K

Yes, yes, indeed, it was still like that, sir, yes, yes, that happened last time. I was coached by one of the division heads; he passed away, right? So, I take care of it there, sir, so it is indispensable for us to know, even I do a risk analysis every week because it has not been a daily yet, sir, so because access to data and information is also limited, we can only control it every Sunday.

The second incident was in 2017. Well, there was an oil crisis then, and in the end, all Bell's sales worldwide dropped from an average of two hundred helicopters per year; this special Bell 412 became only one pack per year. Finally, all POs sent by Bell were postponed. So, at that time, I calculated that the income revenue was only $ 40,000 per year, sir, while to finance this project, it was impossible to pay my salary; it was not enough. Close, I am a bit stubborn, sir. Why do I ask permission? Because from a profit perspective, this doesn't make sense, and if we continue, the company could make a loss, so that's just where the director of commerce scolded me because he saw it from a broader view of the helicopter, Interviewee K can't be stopped because this is INDUSTRIAL DOMESTIC CONTENT, this is a state asset that must continue to operate even though the current costs cannot be met, that's the revenue.

Interviewer

This decision is a strategic decision.

Interviewee K

The strategic decision was made because the EBITDA was small and insufficient to cover the costs; yes, that was the term. So, in the end, it was continued, but with one condition, sir, that I would ask the terms of the commerce director, the same as the Bell. OK, if, for example, it's like that, from $ 40,000, I don't want to send it monthly; I want to send it only once a year, so the production costs are only once, it will be more efficient than the second one is to replace all the old row material. Hence, it is a safe material, we will reimburse them, and they must pay for it. The second condition is the third. I do not want to pay fees for every audit, sir, so they are paying for the audits for the tests. For certification, I mean production certification, so thank God. That year, the project was successful and ended up being a bit profitable. Hopefully, this is the only positive project, sir; the EBITDA will be 340.

Interviewer

Yes, but at that time, you only thought about the project's profitability; you were not familiar with the term EBITDA.

Interviewee K

Not yet, sir, not yet just recently, I understand EBITDA. Interviewer

If everyone already understands EBITDA, including the younger people, and the company informs them about the target EBITDA and the actual achievement, there will be no more debate, right? If everyone already knows that, then yes.

Interviewee K

That is right, that is right, just that there was no knowledge about EBITDA before, right, sir? So, I remember, wow, do not lose, do not be late, that is all on my mind, sir.

Interviewer

That is right, right.

Interviewee K That is, it, sir.

Interviewer

What else do you hope for the project's profitability at The company and enterprise progress with integration from EBITDA to Earned Value Management. What are your hopes?

Interviewee K

All right, sir, I hope that with this integrated information, the benefits are related to the first, COMPANY can sustainably maintain the profitability of all its businesses, sir so that it can continue, even as a company. Now, apart from what was previously, the second is that I hope that in every project, the company can see the benefits from the profit margin. Then we must adjust our product price compared to other companies because I have noticed that our product price can cover better employee salaries. We can ask our people to deliver EBITDA that enables the company to give better wages to the employees. I hope we provide employees with clear information about EBITDA so that they participate and control it.

Interviewer

Yes, so you can also make strategic decisions, right, sir?

Interviewee K

Yes, that is right, the third one earlier. So, if, for example, there is information related to this epidemic, the directors or other management, when something is described earlier, yes, project development can make the right strategic decisions, sir, so there is no misinformation like that. Yes, those three, sir, which we hope COMPANY,

Interviewer

All right, sir, let us finish the interview first, thank you very much; I beg your permission; I will turn off the recording first, OK, sir?

Interviewee K

Yes, please, sir, thank you.

INTERVIEW Interviewee L

Interviewer

All right, Interviewee L.

Good afternoon, good evening. We ask permission to do this interview to write a case study on implementing EBITDA with Earned Value Management for Indonesian Aerospace project management because COMPANY is a project base company. If a COMPANY wants to increase its EBITDA, of course, the company should increase EBITDA in its project management.

Then, in this case, the definition of project management is project management managed by COMPANY. Then Earned Value Management, you already understand that this project control system exists at COMPANY.

And WBS is the Work Breakdown Structure implemented by project management at COMPANY, while the Work Package is the Work Package used to run the WBS. T

hat EBITDA, uh., EBITDA margin is the EBITDA divided by revenue.

Then, I tried to use a 7 Likert scale in this interview if there was a statement that if you strongly disagreed with the statement, the scale is one.

If you strongly agree, the scale is seven. On a scale of 1 - 7, scale two disagreed, one strongly disagreed, two disagreed, three somewhat disagreed, four were neutral, five somewhat agreed, six agreed, and seven strongly agreed. OK, let me start with the questions, Sir, OK?

Interviewee L Please, Sir.

Interviewer

EBITDA is an appropriate measure of project management profitability because EBITDA is operating profitability before paying interest, taxes, depreciation, and amortization. And that EBITDA value is not affected by interest, taxes, depreciation, or amortization. And the value of EBITDA is only affected by efficiency, inefficiency, or productivity in project management. What do you think, Sir?

Interviewee L

I Strongly agree, Sir. So, it is true what Pak Interviewer said.

Interviewer Seven, Sir, yes?

Interviewee L

Seven. Right, Sir.

Interviewer

Good. Then, EBITDA is an appropriate measure of project management profitability because EBITDA is entirely under the control of the Project Manager and Team. Your opinion?

Interviewee L

I Strongly agree because it is complete control rather than project management, Sir. So strongly agree scale of seven, Sir.

Interviewer

Seven, yes. Seven. Good. Question number three is how companies can integrate EBITDA into Earned Value Management

to determine the revenue plan from the budget. So, the Airbus {project aerostructure) budget is the Company's revenue plan. Boeing's {project) budget is {a) the COMPANY project's revenue plan. Then we first determine the planned EBITDA. After EBITDA reduces the budget, we can determine the planned costs. So, the cost is not consuming the entire budget, but we must set aside EBITDA. According to you?

Interviewee L

Strongly agree, Sir, so that the costs can be controlled, and we have a margin, OK?

Interviewer

Then number four.

Companies can integrate EBITDA into Earned Value management by setting a target EBITDA margin on their projects, then distributing it; each WBS has an EBITDA margin, then Work Packages also have an EBITDA margin. According to you, Sir?

Interviewee L

Strongly agree that each operational process has an EBITDA target.

Interviewer

OK, OK, Sir. Fine. Let us go. Then, companies can benefit from using EBITDA as a measure of project management profitability because EBITDA is the profitability of general financial statements, so if we provide EBITDA indicators in projects, other management will know the project's profitability and can understand the project's achievements. According to you?

Interviewee L

I also agree because it is easy to see whether we lose or profit. That is from the EBITDA control earlier, Sir. Strongly agree, Sir.

Interviewer

Yes, yes. Then, the company also benefits from using EBITDA to measure project management profitability. That way, the project manager knows the EBITDA and the EBITDA margin, and the team can also see the target. Thus, they can maximize the profitability of project management. Your opinion?

Interviewee L

Strongly agree, Sir. It is mandatory for us, yes.

Interviewer

Yes, Sir. The next question is,

To increase support for project management by supporting units, project management or project managers need to submit actual EBITDA reports compared to the targets to support the project. According to you, Sir?

Interviewee L

Strongly agree. So that we share with the support system, OK? So those who support us also need to know this information, Sir.

Interviewer

Yes, yes, yes. Then, to make it easier for the Board of Directors to provide timely strategic decision support, the Project Management must also report the results of the achievement of EBITDA and actual targets to the BOD so that he can provide direction and can assist with decisions that are sometimes beyond the project manager's authority. According to you?

Interviewee L

Strongly agree. Yes, BOD should know, too, Sir, to get their support.

Interviewer

Good. So, these were eight structured questions. Now, please tell me the importance of EBITDA as an indicator of project management at COMPANY and what you hope Company's project management will be like with the integration of EBITDA with EVM. Please, Sir, please.

Interviewee L

Yes. So firstly, I support the idea that EBITDA integration is implemented in the project so that we know, Sir, that we have limitations. That means we can control costs, especially efficiency, so we can maintain this EBITDA margin because, without it, we usually do too much, Sir. Yes, we assume that we still have a budget like that. So, we continue to use the budget; it is as if, yes, the important thing is not to exceed revenue, right? But with this EBITDA, we can see that we must have a margin that we have set together and do it well. The hope for the future is that project managers and program managers can more easily control it and that every related function that supports us knows the information needed so they can keep it even more, Sir. That is, it, Sir, from us.

Interviewer

Good. Then in implementing the integration of EBITDA into EVM, what are you doing, Sir? With IT, with the project manager, with finances, what {need) to do, Sir?

Interviewee L

Yes. In addition to installing the first one, each target in each WBS, because after all this project, we must split based on the WBS, right, Sir? This WBS can be made from level one to the level below it. There can be up to four or five, and in each WBS, we set the target so that everything is integrated from the top to the lowest level, even if the operators understand the concern about this EBITDA margin.

Interviewer

Good. With this concern, what do you hope for with EBITDA in EVM for a company that., manages aircraft manufacture? What do you expect, Sir?

Interviewee L

So ., we can already know by implementing EBITDA long before cost overrun occurs, Sir. So please do not go too far waiting for the end of the year, then you will know you are losing, that is it. With this EBITDA - EVM integration, we can control project EBITDA, and before the project losses worsen, we must take action. Action something, take what to fix it so that we return to the margin that we agreed on, namely the EBITDA margin earlier, Sir. So, it is much easier to control and can be managed earlier than if we wait for it to happen first, and then we will find out about it later. Well, this is the important thing, why do we need to implement this so that in advance we can control day by day the problem of what is the name of this project, Sir?

Interviewer

Then what are the benefits of the relationship with accounting, with other supporting units, Sir, do we tell them that? Please.

Interviewee L

Yes. First, the units will support us when we experience difficulties; for example, what causes this overbudget? Oh, I see. At the operator level, for example, or at the material or financial status, what might be called, the provision of funds is not proper, and so on. So, from the start, we have conveyed information they can prepare better.

Interviewer

OK, Sir. Erm, any more messages, Sir? What are your hopes for this company that many Indonesians love, Sir?

Interviewee L

We wish this to be a success, Sir. So that in the future employees will be more prosperous, Sir. With this success, I am sure the company should not be stingy anymore. It will provide many conveniences and even sustenance to employees. That is all, Sir, from us.

Interviewer

OK, Sir. Ee ., thank you for allowing me to interview you. Hopefully, this can also be a lesson for other friends. If the case study is good, we can also set an example for other companies to integrate EBITDA with Earned Value Management. Thank you, Sir. Good afternoon. Thank You.

Interviewee L

Thank you, Interviewer, for your help. Good afternoon.

INTERVIEW . Interviewee M

Interviewer

OK, thank you for your presence in this interview. From this interview, we will conduct a Case Study on how COMPANY applies EBITDA for EVM with IT SYSTEM.

Let's start; there are two sessions; the question session is like a questionnaire with a scale of 7; if the statement, according to the Madam, strongly disagrees, the scale is 1, and the scale 7 strongly agrees. If 1 strongly disagrees, 2 disagree, 3 somewhat disagree, 4 neutral, 5 somewhat agree, 6 agree, and 7 strongly agree.

After this statement, Madam spoke freely. The first is a Structured Interview.

Let's start, Madam.

Interviewee M

Please Interviewer

Interviewer

Your voice is small.

Interviewee M It's unheard of.

Interviewer

Good. EBITDA is a proper measure of Project Management Because EBITDA is operating profitability before payment of interest expense, depreciation tax, and amortization. Interest rates, taxes, depreciation, and amortization do not affect EBITDA value. However, the EBITDA value is only influenced by the efficiency and productivity of project management.

Madam, what is your opinion on a scale of 1 to 7?

Interviewee M

In my opinion, I strongly agree with Interviewer.

Interviewer Strongly agree yes.

Interviewee M

The scale of 7, yes, the scale of 7.

Interviewer

The second statement.

EBITDA is the proper measure of Project Management profitability because EBITDA is entirely under the control of Project Management. So, it's not under the control of the Board of Directors.

Madam's opinion?

Interviewee M

Strongly agree, Interviewer. EBITDA is under the control of Operational Project Management. Apart from the Project Manager as well as the Team. In our place, it includes PPC.

Interviewer

OK, Madam, Next, the company can integrate EBITDA into EVM. The first is to set a revenue plan from the Authorized Budget. So, the Authorized Budget from the BMPP {Barge Mounted Power Plant) is the Project Management revenue plan. Then, by setting EBITDA, we can only get a cost plan.

So, the budget plan is not to spend all the existing budget plans. So, the way is, the company can integrate EBITDA by setting the Budget as the Revenue Plan, selecting the EBITDA plan, and then from the rest, it is the costs plan that Project Management will manage.

According to Madam?

Interviewee M

Yes, Interviewer Leres, I agree.

Because it is mandatory to run a project, the EVM method taught by Interviewer is compulsory. Because, after all, both planned and actual realization must always be controlled for cost planning and tracking. So strongly agree.

Interviewer

OK, Madam Scale of 7

Then the company can integrate EBITDA into EVM by setting the EBITDA Margin target in Project Management. Then in each WBS and Work Package also attached the respective EBITDA Margin.

What do you think?

Interviewee M

Yes, I strongly agree, Interviewer

So, in COMPANY, it is related to the WBS or Work Breakdown Structure in every project; the details inside contain Work Packages. And in each activity, we must think about how much % of EBITDA must be generated. So, we don't necessarily make a Purchase Order to adjust the existing Budget; it doesn't. The minimum must follow the respective Budget. For example, we have a Budget of 10 and an Aspirational Margin of 20%. So, for the work package to third parties, we must leave a minimum EBITDA of 20%. In each Work Package, sir. strongly agree.

Interviewer Madam, thank you.

Then point 5, the company benefits from using EBITDA as a measure of Project Management profitability because EBITDA is a standard measure of profitability in financial statements. Everyone knows. Everyone is used to it.

So, by including EBITDA in Project Management, Project Management, who does not understand EVM terminology but can read the financial statements, can understand the project's profitability.

Madam's opinion? Interviewee M

Strongly agree, Interviewer. Indeed, a company's healthy or unhealthy picture can be seen from its EBITDA. So, what is the financial condition like? The main KPI to read is EBITDA first. There, we can issue future strategies like what, for example. For example, what approach should we take if the EBITDA condition is lower than the Plan?

And God willing, the evaluation should be routine per quarter, Interviewer. So, I think.

Interviewer OK, Mom,

Then the company benefits from using EBITDA to measure project management profitability. Because that way, the Project Manager and Team can determine the EBITDA. To maximize PM Profitability

Madam's opinion?

Interviewee M

Yes, I strongly agree, Interviewer. Indeed, the measure of project management profitability is from EBITDA. Especially if, in our place, there are ongoing Projects.

So indeed, the Project Manager and his Team, as well as friends from the supply chain, support Interviewer,

Especially because the highest content of our project is materials and services, around 60%, that's what they really need to understand. What's the profitability like? What was his achievement like? The goal is to understand EBITDA and know what they must do for efficiency.

That's Interviewer

Interviewer OK, Mom.

Next, to increase support from supporting units to project management, the project manager must report actual EBITDA achievements and targets to support teams throughout the company.

Madam's opinion?

Interviewee M

Yes, strongly agrees.

; Interviewer

It is a Habit that we must develop. Indeed, we only report monthly, weekly, and even daily, sir. So, the obstacles that occur in the field have been reported daily to the top.

So don't wait until one month, then gather; only then will the board of directors find out. The boss knows that because time is valuable for project management.

That's it, sir.

Interviewer Good

To make it easier for BOD to provide direction and timely strategic decisions to project management, the PM also needs to report the actual EBITDA and target EBITDA so that he is aware and can then make decisions that may be beyond the authority of the Project Manager.

Interviewee M

Strongly agree, Interviewer. Because the average project manager can't decide. So, the decision is by BOD, so EBITDA must be reported. What is the actual like, then what is the Gap from the Plan like?

What sort of issues must be raised? Interviewer must also be detailed and not piece by piece. Because fellow project managers sometimes fear late delivery, they cannot make decisions, right?

And this is the hope that with this EBITDA Maximize, everyone knows every point of the business process has a problem. Must be reported to superiors. Especially if the level of the division head cannot be resolved, then the problem must reach the BOD level to get a decision.

That's Interviewer's input from us.

Interviewer

So, what's the scale of mom?

Interviewee M

7 Interviewer

Interviewer

All right, Madam, the structured interview is over.

Un-Structured. What are your hopes for Project Management profitability at COMPANY with the integration between EBITDA and EVM? Madam, free story, please, Madam.

Interviewee M

All right, Interviewer, thank you, Interviewer, for the time given.

Feedback about the project. Interviewer has many projects at PT PL, especially this Marketing Director, who is currently working with the UAE to provide projects to COMPANY is related to foreign defense projects. So, there are many strategies that we must implement, also associated with everyone's performance. So, it's not just project management but also all structural lines. So maybe it's like what Pak Interviewer taught us about communication, coordination, and cooperation must be encouraged.

Because of this, thank you, sometimes one person knows, the other doesn't know; this is true; communication must be intensive. And they must be proud to accept all orders and strategies from the company. Must implement consistently. Sometimes because of Pak Interviewer's communication, the process doesn't work. So, we must remind each other of the importance of coordination, communication, and consistency that we must build continuously to support BOD programs.

That Pak Interviewer might be one of them. Also, a concern for fellows, especially COMPANY members, and all employees. The importance of EVM and EBITDA, many people in the fieldwork, not that they don't care. It's just that they don't have enough knowledge about their EBITDA. Some of them are like this.

Suppose he is a welder, welding; he must know his schedule; one day, he must weld two meters. From one day's target, he had to solder two meters; he only welded one and a half meters. So, why is half a meter lacking? What's the problem? Possibly the welder didn't report to their immediate supervisor, namely the head of the section, that the electrodes were used up there. Or maybe there was an electricity blackout, and he might be tired or sick. Things like that may have to be communicated frequently, right?

Maybe these little things might reduce idle capacity or idle activity, which can create many indirect hours that are made, sir. Yes, maybe once again, we must continue raising awareness among our friends as leaders. That's Interviewer from me.

Interviewer

Well, then, your fellows' knowledge about EBITDA, about EVM can motivate them to be more responsible and have a sense of ownership and motivation. What do you think?

Interviewee M

Yes, that's right, Interviewer, with the knowledge of yesterday's fellows.

Following EBITDA Maximize, EVM, especially now that there is a mandate for implementing IT SYSTEM by system on HP. So, one person holds a cell phone, and 1,500 are sent to fellow employees, where we must fill out a time sheet. The first is attendance; then the second is Pak Interviewer's timesheet, and the timesheet is their daily activities. Of course, everything must be corrected or validated by their respective superiors.

So, it doesn't mean they fill one 7-hour working day, we don't acc. Let's review again, is the activity following the output written there?

So, the role of the supervisor is very dominant, Interviewer. This role of supervisor is also applied to EBITDA and EVM control. So, there are inputs and outputs, Interviewer,

Interviewer

OK, Madam, lastly, what are your hopes for increasing the profitability of the Company , with discipline your friends carry out EBITDA control with IT SYSTEM and EVM; please, Madam.

Interviewee M

OK, Interviewer, ready.

We hope that with the running of the IT SYSTEM at COMPANY, the CEO has been buzzing about it as of June 1 yesterday. It will increase profitability, so those friends know the value of their presence in COMPANY they are aware of what output must be done. That their every move contains money.

So, they haven't been aware all this time; I'm sorry, it's our culture; once they're present and after they're absent, that's it; the responsibility has been paid off.

But with this IT SYSTEM, it will be read what this clock is doing and what it is doing like that.

Even though there are obstacles later, it will be written there; for example, he has a ceremony, there is a meeting that is illustrated in IT SYSTEM their respective superiors will validate the activity per person. The hope is that the profitability aligns with the target of more than 10%. Well, yesterday, our target was 10%. It must be 20%, like the target conveyed by Interviewer. Like that Interviewer

Interviewer

OK, Madam, I thank you for allowing me to interview you. I beg your permission to turn off your video recording.

INTERVIEW Interviewee N Interviewer

All right, Interviewee N, the explanation is that COMPANY manages project management. Earned Value

Management is an Earned Value Management system implemented for project management at COMPANY. WBS is a Work Breakdown Structure system that is implemented in project management at COMPANY, and Work Packages are Work Orders used to conduct WBS. EBITDA is earning before Interest, Tax, Depreciation, and Amortization, is income or profit before paying interest and before paying taxes, depreciation, and amortization. For EBIT, it includes depreciation and amortization. And the depreciation and amortization can be increased or decreased by the directors.

Meanwhile, EBITDA cannot be affected by depreciation and amortization. And the EBITDA margin is the EBITDA value divided by the revenue. Then in our question, there are two sessions, sir. The first session is a structured interview with a scale of one strongly disagree, then seven strongly agree. Among them, two do not agree; three somewhat disagree; four neutral; five somewhat agree; six agree; and seven strongly agree. May I begin, sir? Any questions?

Interviewee N bismillah, sir.

Interviewer

OK, sir. EBITDA is an appropriate measure of Project Manager profitability because EBITDA is operational profitability before payments of interest, taxes, depreciation, and amortization. And EBITDA is not affected by interest, taxes, depreciation, or amortization. And the EBITDA value is only influenced by the efficiency and productivity of project management. What do you think?

Interviewee N

My opinion, I strongly agree, sir.

Interviewer

Strongly agree, yes. Good. Seven, sir, yes?

Interviewee N Scale seven, sir.

Interviewer

Good. Then secondly, EBITDA is a measure of profitability from proper project management because EBITDA Project Management is entirely under the control of the Project Manager and Team. Your opinion?

Interviewee N

Strongly agree-on a scale of seven.

Interviewer

Scale seven. OK, sir. Then, the company can integrate EBITDA into Earned Value Management by establishing first the revenue plan, the project management income plan if the project is Airbus, (then) the Airbus budget is (is) our revenue plan. Then set its EBITDA plan. For the rest, we can get a cost plan. Your opinion?

Interviewee N

Strongly agree, sir.

Interviewer

Strongly agree. Good. Fourth, the company can integrate EBITDA with EVM by setting a target EBITDA margin in the project, breaking it down, and distributing it to each WBS; the Work Package has an EBITDA margin so that implementers also know the target. What is your opinion, sir?

Interviewee N Strongly agree.

Interviewer

Strongly agree. OK, sir. Point five, the fifth question is that companies benefit from using EBITDA to increase company profitability through a capacity matrix. Companies with a project management process with positive EBITDA can increase their EBITDA margin by using the capacity matrix. We can analyze which processes are bottlenecks or over-capacity with the capacity matrix. Thus, steps can be taken to improve the process to increase the Operating Profit. What do you think?

Interviewee N

Strongly agree, sir.

Interviewer

Strongly agree. Good. Ee, next, the sixth question, companies can benefit from using EBITDA as a measure of profitability from project management because then the project manager and team know their respective EBITDA targets in the WBS Work Package. Thus, it can maximize the profitability of project management, of course, in the corridors of delivery, quality, and safety. What is your opinion?

Interviewee N Strongly agree.

Interviewer

Strongly agree. OK, sir. Then, the next question, the seventh. To increase support from Company's supporting units to project management, the project manager needs to report actual EBITDA achievements compared to the EBITDA target, then together with the EBITDA matrix and Capacity matrix and fishbone analysis, issues or problems that can be found in business processes. Based on this, alternative solutions can be provided by using the Toyota Way. Opinion, sir?

Interviewee N

Strongly agree, sir.

Interviewer

Strongly agree. Then next, to make it easier for the Board of Directors to provide timely direction of decisions to project management, the Project Manager needs to report the actual achievement of EBITDA and its EBITDA target to BOD. Thus, the BOD can provide direction and support for strategic decisions that the project manager sometimes does not own. What is your opinion?

Interviewee N

Strongly agree, sir.

Interviewer

OK, sir. Er, structured questions are over, sir. Please tell me your hopes for implementing EBITDA with EVM in project management to COMPANY. What is your hope?

Interviewee N

Sir, permission, I hope that the application of this EBITDA, its application can be conducted in all lines, sir, especially in areas that directly interact with the achievement of EBITDA. The projects we are currently conducting can be fully supported by functional; what are the names of organizations that directly conduct functional support, sir? In today's experience, we only pursue delivery, never considering whether this project is a loss or a profit. With the implementation of EBITDA from the start, daily EBITDA, at least, we have started to be able to control from the start whether the project or this project, in conditions, is good or bad at cost, reasonable EBITDA or not. And there is already a set target. Of course, since the beginning of conducting bidding to get contracts, this EBITDA must have been kept at the forefront of 25 (percent), 26, or whatever the number is. This target must have been stored there from the start so that the project doesn't run, only now we force EBITDA to be entered at a particular value while when compiling the project and offering the project, we don't take that into account, only take a margin of 10%, and after that, it's not enough only determines a 10% margin but there are also other costs that we need to consider. So, I hope this EBITDA can be fully applied to all projects at the COMPANY. That is, it, sir. Thank You.

Interviewer

OK, sir, with your position as project manager, what are your hopes for implementing the EBITDA concept, sir?

Interviewee N

Ready. Of course, we hope project management under my supervision can undoubtedly provide an EBITDA Margin of 25% or follow management's directions. For this reason, we are also implementing a bottom- up budgeting pattern so that we can review the processes to achieve this target. The EBITDA margin may be below this value in specific processes but can be compensated by margins in other processes. Or make improvements so that the process can increase its productivity or make the process more efficient. As a project manager, I also direct so that there are no delays in the delivery of this project's output while maintaining the project's quality according to the scope/requirements agreed with the client.

That is, it, sir. Thank you.

Interviewer

OK, sir. If only with Earned Value Management indicators, let us say with CPI, with schedule variance, many people do not understand, especially in accounting then in marketing, with EBITDA as a measure of profitability that is common in financial reports, do you think it helps them understand or what, sir? Please.

Interviewee N

Er, today fellows at, especially in accounting, sir, we have been working together since the beginning. Um, we do a weekly review, sir. Eh, and we get information about EBITDA, sales achievements, then what are the targets from our Annual Budget so that our EBITDA will appear negative or positive in the end. That can do it, sir, yes. It is just more reporting, I see. And the expected things do not appear, such as alerts for other supporting functions. And right we should also find out why he did not reach his EBITDA. We should be looking for the root cause, the deepest one, so we can fix it. Well, this often does not happen, sir. So weekly it happened, a review took place, but there was no explicit action from the meeting results, so the

following week when we reviewed the position again, there did not seem to be any significant changes like that. It is like we are just listening to reports. After that, it just goes by. So, this is what I think is the problem. We should have acted on what was reported that we had to follow up on to improve the current condition so that it was not in the end that we found out that, oh, it turns out that this project made a loss. So, we need to cooperate with them so that we know exactly where we are every week, and we must know what to do to improve the situation that has occurred so that we do not lose more from projects that are currently running. That is, it, sir.

Interviewer

With Earned Value Management, we can see the condition of the project at any time, but so far, the EVM indicator has not shown profitability. So, by attaching EBITDA or profitability to EVM at any time, not just daily, um, the whole organization can see it so that whatever difficulties there are in the field, they can be immediately assisted and agree, as you said earlier, that they are not just watching, but they are involved to help with the difficulties that are in the field. Then with the profitability indicator of the project at any time it can be seen by the board of directors, do you think there are any benefits or not?

Interviewee N

Er, extremely useful, sir, in my opinion. So, the directors can also see the performance of the projects, right? It should be monthly, right? So, every month, gentlemen on the board of directors can see the performance of each project. This information also includes communication that makes, um, later, there will be policies taken by the board of directors when we experience problems that we cannot solve under circumstances related to working capital. So, working capital can be my biggest problem now because it will impact procuring payments for these materials so that they arrive on time. And in my opinion, this is especially important, sir, to be opened to the BOD, to be seen by the BOD so that the BOD does not only see the results but also takes control of the travel phase over a period of months, that is it, sir.

Interviewer

Yes, they can monitor the performance of each project through the EBITDA dashboard prepared by IT. So, you can do a go-see or visit the site directly by IT. If necessary, be able to communicate effectively directly with the field team based on the same dashboard to improve the quality of management communication and project managers or other related teams. OK, sir.

Interviewee N

Maybe, sir, if we meet and the BOD sees it, at least there will be an interaction like that, sir.

Interviewer

Yes sir. Yes sir. Yes, Then, what do you hope with the integration of EBITDA into Earned Value Management for COMPANY projects, sir?

Interviewee N

I strongly agree, sir. Well, I have done, sir. So, gentlemen, all those supporting can see what is going on with ongoing projects, and that can, uh, what is it called, make a correction or a directive that makes this project run the way we want it to. Want. Sir. I completely agree that this can be done.

Interviewer

All right, sir, thank you for your time and permission to be interviewed. Especially useful for the COMPANY and its improvements. That is what I say. Please let me turn off the recording, sir. Yes.

INTERVIEW Interviewee 0

Interviewer

All right, 0, thank you for coming.

We will use this interview as data for compiling a Case Study on the application of EBITDA at the company with EVM for project management with IT System

Project Management is Project Management managed by a C0MPANY. Suppose Earned Value Management is a system implemented by C0MPANY for Project Management. Meanwhile, the Work Breakdown Structure is the WBS implemented in C0MPANY Projects. Then the Work Package is the work order used to implement the WBS. While EBITDA, you are a finance expert.

Then we divided this interview into two sessions. The first session is a structured interview. There are seven scales where you will express your opinion from strongly disagree to strongly agree. Scale 1: strongly disagree, Scale 2: disagree. Scale 3: slightly disagree. Scale 4; neutral, Scale 5: Somewhat Agree; Scale 6 Agree. Scale 7: Strongly Agree. We use the scale to answer structured questions.

So, the first statement: EBITDA is the proper measure of Project Management profitability because it is 0peration Profitability before payment of Interest, Tax, Depreciation, and Amortization. And EBITDA is not affected by interest, tax, depreciation & amortization. However, EBITDA is only affected by the efficiency and productivity of Project Management.

Your opinion? Agree or disagree.

Interviewee 0

Strongly agree with me.

Interviewer 0K, 0K, 7, sir.

Interviewee 0

Yes 7

Interviewer

Then EBITDA is the proper measure of Project Management profitability because EBITDA is entirely under the control of Project Management. From start to finish. Your opinion?

Interviewee 0 Agree agree.

Interviewer

Agree, the Scales?

Interviewee 0

6, 6

Interviewer

Six, sir, 0K, the company can integrate EBITDA into Earned Value Management by determining the revenue plan from the Authorized Budget. So, the company does not consider the BMPP budget from PT Indonesia Power to be Costs, but the company thinks the authorized Budget is Planned Revenue. Then the C0MPANY can set the EBITDA, and the rest is Planned Costs.

So, in the current EVM, the Budget is the Planned Cost, so there is no margin. This perspective suits the project owner. But for contractors, for project implementers, Revenue is the Budget, then we set aside EBITDA, and the rest is planned costs.

What is Your opinion?

Interviewee 0 Agree

Interviewer The Scales?

Interviewee 0

6

Interviewer Good

Then the company can integrate EBITDA into EVM by setting EBITDA targets in Project Management. Then the EBITDA Margin targets are broken down into EBITDA Margin targets for each WBS and Work Package. So, all WBS has a target EBITDA Margin, and all Work Packages have a target EBITDA Margin. So that everyone knows that the EBITDA Margin of this project is that much.

Your opinion?

Interviewee 0

7, 7 Strongly agree.

Interviewer 7, sir, Fine. Then,

The company can benefit from using EBITDA as a measure of Profitability in Project Management because EBITDA is a measure of profitability from standard financial reports. Thus, if EBITDA is applied in Project Management, then all management in the company knows how much the profitability of project management is.

If there is no EBITDA, Management outside the Project does not know Project Management's profitability. What is EBITDA?

So, according to you, the company will benefit by attaching EBITDA to Project Management. Your opinion?

Interviewee 0

6, agreed

Interviewer

Six, 0K. Then furthermore, the company benefits from using EBITDA as a measure of Project Management Profitability. Because then the Project Manager and team know the target and can maximize their EBITDA. Those who previously only knew Costs, Quality, Delivery & Safety. Now there is a targeted Margin.

According to you?

Interviewee 0

7, Strongly agree.

Interviewer

Strongly agree, good sir.

Then to increase Supporting Units support for Project Management. The Project Manager needs to report the achievement of actual EBITDA and targets to all management supporting units so that he can participate in supporting the success of project management.

Your opinion?

Interviewee 0

6, agreed

Interviewer

6, fine. Then to make it easier for the Board of Directors to provide timely strategic decisions and Directions to Project Management, the Project Manager needs to report the achievement of actual EBITDA and its Target to B0D. Thus, the B0D can provide direction and make strategic decisions that are sometimes beyond the authority of the Project Management.

What do you think?

Interviewee 0

7, Strongly agree

Interviewer

All right, sir, the structured questions have been completed, then I ask for your opinion. What are the benefits of implementing EBITDA in Project Management with EVM now integrated with IT SYSTEM (information technology system) for C0MPANY?

Please sir

Interviewee 0

Yes, Interviewer, as we know before, this C0MPANY, if we talk about IT SYSTEM, called the IS early, is almost the same. There is a bit of a difference from the earlier version, IS had more people to key in, so there was a delay. We hope that the IT SYSTEM that we are developing will be a success. Everyone is immediately involved. That is what significantly differentiates. Then, because everyone is directly involved, the whole level is concerned.

So, in connection with the structured question, it becomes relevant.

Everyone knows what the terms are, a portrait of what was done, then what contribution is reflected in the company. 0f course, it all depends on leveling. But this portrait must be known by all lines, which is now the differentiator. I guess so, in my view. It was regarding Interviewer's question.

Interviewer

0K, Interviewee 0, what are your hopes for implementing EBITDA in Project Management? How can this IT SYSTEM integration, with EBITDA and EVMbe implemented at every WBS? Every work package has an EBITDA target. What do you hope this can increase Company's EBITDA?

Interviewee 0

Yes, so this is true when we talk about EBITDA.

This has something to do with behavior, yes, Human Capital behavior. 0ccupation, I say Scales 7 and 6 are thin because the difference is in behavior. Because as I see it, it is related to behavior. If we look at the history of the Company, it was an ordinary service company that was a non-profit-based entity. 0ne day it entered a limited corporation, a profit-oriented entity. So, these people are still getting acquainted with non-profit organizations into profit organizations-something like that.

So now we are aware because there is a new system. The system usually involves many comfort zones. Why did I say to behave? Usually, if people are judged inefficient, they will refer to the inefficiency caused by the system or that it is not perfect. Therefore, from the side of the person holding it.

0n the upside, this is why it is so important to have a top-down process. That is, it; how do we ensure we are both happy to learn this system?

This EBITDA becomes something important, then integrated into the system. So later, we will not talk about reward and punishment first. The habit is to do it. Later after everything is running, the end will be reflected. That is how it is.

The behavior will automatically become normal after this habit feels the importance of EBITDA. That is, it. I remember, for example, similarities when we studied payment at T0LL. The pay-to-use System was inconvenient. Go to the ATM too.

People who did not hold their bank transaction record book in front of the counter did not feel comfortable-something like that.

In terms of EBITDA implementation, it is somewhat similar. It concerned human capital. So, human behavior is my homework.

All fellows know that EBITDA is important, and it is under control. But this knowledge becomes essential when Interviewer reminds them.

They already have this knowledge. What is the definition of Direct, Indirect, and responsibilities? They already know. But with Interviewer's entry to renew, what is Maximizing daily EBITDA (EBITDAMAX) is integrated into the system.

To make this system work, I have homework in Human Capital.

Because this concerns organizational behavior, that is what I see Interviewer.

Interviewer

Good. If the finance person already knows EBITDA from daily work. But the people of production, engineering, procurement, and logistics do not know this. Many are not familiar with EBITDA. With this introduction that EBITDA is their responsibility, according to behavior. In your opinion, how can this improve the company's performance? Please sir

Interviewee 0

So, there must be a common perception among production people. The fundamental understanding is that if you take a picture on the outside, it means a portrait of the comprehensive financial report, the term, according to the accounting person, is like management accounting. What we are currently photographing and pushing for is management accounting; that is how it is. It will also come out as accounting. Still, management accounting uses EBITDAMAX. In other words, it is more management accounting. Although in the end, it will be reflected later in financial accounting. Something like that.

Therefore, in my experience, if I have only been here for a while, the terms may equate to perceptions first.

For example, suppose there is someone who has project management in Ship Maintenance and Repair. He prefers to subcontract work rather than hiring permanent employees. So. Because at the time

Make a cost structure and determine which is the most efficient.

If the project employs someone whose cost is more expensive, even though the work is not like that, in the term later, part of the transfer costs must be transferred to corporate financing. Well, I think so.

That is all. As for information, Interviewer, the problem is just there. Meanwhile, instead of being idle, enter a project that can be worked on. Corporate financing is like that, so the double cost is like that.

We pay idle people because they do not go into Maintenance and repairs, just as we do sub-contracting to other companies. So, an understanding of EBITDAMAX sometimes is based on these things. EBITDAMAX means it must be a project manager, so think about my imagination. For example, a forklift picks up goods once. 0ne time it should be able to take three items, but only take one. I think so. And tomorrow comes again.

Friends are blaming each other in operation. Maybe Pak Interviewer means I understand. Maybe friends also understand. But because of that earlier, the management did not monitor the EBITDA appropriately, but it has been getting better over time because EBITDA is integrated with the system so that people will see. It is not wrong to blame, but this needs to be seen.

The forklift lifts one item, why not three goods, the supply chain people are to blame. Supply chain people do not want to be blamed because the finance department has not paid the money related to the vendor. So, in the end.

So, blaming each other, in total EBITDA in terms of Corporate Financing, has been eroded.

But if the portrait is related to the project management person's EBITDAMAX, maybe he has a reason. It can be seen why only one item is transported. In the future, he will have to talk to the supply chain people, and in the end, there will be an overall improvement.

The direction looks like my friends understand. However, what needs to be built from the Top-Down is that we both understand this, but communicating the search for solutions involves many things related to organizational behavior. In this case, Human Capital needs to create homework.

That is my view as short as I follow it.

However, at the executive level, he is still comfortable. I want to transport only one item or two for him, and it is the same.

But things will be different once he sees his impactful work on EBITDA. Interviewer

So according to you, with the corridors of quality, delivery, and safety, a target is given to maximize EBITDA within the corridors.

How about this corridor, which aims to maximize EBITDA, which can improve people's behavior in the field?

Because the system requires them to deliver the target EBITDA while fulfilling the target quality delivery and safety?

What do you think?

Interviewee 0

So, it is true, Interviewer, once he knows the target, then he knows the release, automatically, his behavior will change. He cares about himself, his friends, and his unit. I think.

0f course, he will not change the standard delivery time, quality, and safety, and people can meet those targets.

Interviewer

All right, Interviewee 0, this is the last one.

What are your expectations with implementing EBITDAMAX, then IT SYSTEM and EVM in C0MPANY? What are your expectations of the impact on the C0MPANY?

Please

Interviewee 0

So, my first hope is. This system is something relatively new for the Company, I mean. Later, ask Interviewer to do a before and after analysis of its application. We expect it that way.

Then, the whole level becomes something good; you can measure yourself like that. I hope this will be successful regarding this EBITDAMAX because, in my opinion, it is the only way.

In the end, I Strongly agree. There is only a value of 6 and 7, and the deviation is due to the behavior of human capital, my homework. So, my task is to deliver something meaningful. That is, it-all human capital.

But inshallah so.

Because otherwise. This change is somewhat heavy. Because many believe that if the people cannot fulfill it, the company will miss the many opportunities, which will become dangerous for the C0MPANY.

For example, let me tell you a little: soon, many orders will come in. Lots of orders, but what is it,

Interviewer

EBITDA is not reasonable, right?

Interviewee 0

To be precise, the EBITDA at the beginning was well planned.

I did not learn from the previous implementation. That is essential with the tools currently being guarded by EBITDAMAX, EVM, and IT SYSTEM. So that the new contract will encourage people to implement the system, and the company can maximize EBITDA.

So, it is not just that it looks like it is working. It is still working that produces something positive.

Interviewer

There is a report.

Interviewee 0

People will have something in return. It adds value to the company. Employee welfare will automatically get that way if the company's value increases.

If it is not like that, the people will look for additional income from outside the company's EBITDA if it works later. That is a bit dangerous, in my opinion.

Interviewer True, true, true

All right, Pak Interviewee 0, I beg your permission to turn off the recording first.

Interviewee 0 Good

INTERVIEW Interviewee P

Interviewer

OK, Interviewee P, so the definition is as follows:

- project management is project management managed by a COMPANY.
- Earned Value Management is an Earned Value Management system implemented for project management at COMPANY.
- WBS is a Work Breakdown Structure system that is implemented in project management at COMPANY and
- Work Packages are Work Orders used to conduct WBS.
- EBITDA is earning before Interest, Tax, Depreciation, and Amortization is income or profit before paying interest/interest, before paying taxes, depreciation, and amortization.
- For EBIT, includes depreciation and amortization. And the depreciation and amortization can be increased or decreased by the directors. Meanwhile, EBITDA cannot be affected.
- And the EBITDA margin is the EBITDA value divided by the revenue. Then in our question, there are two sessions, sir.

The first session is a structured interview with a scale of one strongly disagree, then seven strongly agree. Among the scales, scale two, do not agree; three, somewhat disagree; four, neutral; five, somewhat agree; six, agree; and seven, strongly agree. May I begin, sir? Any questions?

Interviewee P Yes, bismillah, sir.

Interviewer

OK, sir. EBITDA is an appropriate measure of Project Manager profitability because EBITDA is operational profitability before payments of interest, taxes, depreciation, and amortization. And EBITDA is not affected by interest, taxes, depreciation, or amortization. And the EBITDA value is only influenced by the efficiency and productivity of project management. What do you think?

Interviewee P

My opinion, I strongly agree, sir.

Interviewer

Strongly agree, yes. Good. Seven, sir, yes?

Interviewee P

Scale seven, sir.

Interviewer

Good. Then secondly, EBITDA is a proper measure of the profitability of project management because the EBITDA of Project Management is entirely under the control of the Project Manager and Team. Your opinion?

Interviewee P

Strongly agree-on a scale of seven. Interviewer

Scale seven. OK, sir. Then, the company can integrate EBITDA into Earned Value Management by establishing first the revenue plan, the project management income plan if the project is Airbus, (then) the Airbus budget is (is) our revenue plan. Then set its EBITDA plan. For the rest, we can get a cost plan. Your opinion?

Interviewee P

Strongly agree, sir.

Interviewer

Strongly agree. Good. Fourthly, companies can integrate EBITDA into the project by setting an EBITDA margin target and then breaking it down, dividing it into each WBS, and the Work Package has an EBITDA margin so that implementers also know the target. Opinion, sir?

Interviewee P Strongly agree.

Interviewer

Strongly agree. OK, sir. Point five, the fifth question is that companies benefit from using EBITDA to measure profitability in project management because EBITDA is a standard operating profit measure from financial reports. Thus, company management that is not in project management can understand project profitability. Your opinion?

Interviewee P

Strongly agree, sir.

Interviewer

Strongly agree. Good. Ee, next, the sixth question, companies can benefit from using EBITDA as a measure of profitability from project management because then the project manager and team know their respective EBITDA targets in the WBS Work Package. Thus, it can maximize the profitability of project management, of course, in the corridors of delivery, quality, and safety. Your opinion?

Interviewee P Strongly agree.

Interviewer

Strongly agree. OK, sir. Then, the next question, the seventh. To increase support from the COMPANY supporting unit for project management, the project manager needs to report the actual achievement of EBITDA compared to its EBITDA target to the supporting units so that the supporting units know the difficulties and needs for support. Opinion, sir?

Interviewee P

Strongly agree, sir.

Interviewer

Strongly agree. Then next, to make it easier for the Board of Directors to provide timely direction of decisions to project management, the Project Manager needs to report the actual achievement of EBITDA and its EBITDA target to BOD. Thus, the BOD can provide direction and support for strategic decisions that the project manager sometimes does not own. Your opinion?

Interviewee P

Strongly agree, sir.

Interviewer

OK, sir. Er, structured questions are over, sir. Please let me share your hopes for implementing EBITDA in project management to COMPANY. What is your hope?

Interviewee P

Sir, permission, I hope that the application of this EBITDA, its application can be conducted in all lines, sir, especially in areas that directly interact with the achievement of EBITDA. The projects we are currently conducting can be fully supported by functional. What are the names, organizations that directly conduct, and functional support, sir? In today's experience, we only pursue delivery, never considering whether this project is a loss or a profit. With the implementation of EBITDA from the start, daily EBITDA, at least, we have started to be able to control from the start whether the project or this project, in ee conditions, is good or bad at cost, reasonable EBITDA or not. And there is already a set target. Of course, since the beginning of conducting bidding to get contracts, this EBITDA must have been kept at the forefront of 25 (percent), 26, or whatever the number is. This EBITDA margin target must have been stored there from the start so that the project doesn't run, only now we force EBITDA to be entered at a particular value while when compiling the project, offering the project, we don't take that into account, only take a margin of 10% and after that, it's not enough only determines a 10% margin. Still, there are also other costs that we need to consider. So, I hope this EBITDA can be fully applied to all projects at the COMPANY. That is, it, sir. Thank You.

Interviewer

OK, sir, um, with your position as the authority for project planning throughout this company, what are your hopes for the supporting unit where you can achieve EBITDA, the EBITDA target of the project? What are your hopes for those in the supporting unit, sir?

Interviewee P

Ready. Er, of course, we hope that the supporting unit can.

meet all the requirements we need, among others, and no project does not need a budget. So, this budget must have been secured from the start so that when the need arises, there will be no more stories of looking for sudden funding. That is the first. Second, the application of just-in-time for materials. This

business process is fundamental because there are no waiting stories when we examine the Toyota Way. So, all materials, all tools, and all resources should have been available from the start. Today's events are extremely far from ideal, so we from the production planning function are experiencing difficulties because we must amend planning every week, and this is not particularly good because, in the end, it will be challenging to control the EBITDA that we have planned. So, the function that I need the most is the function that controls finances and material functions that must be more initiative-taking to support our activities in the project. That is, it, sir. Thank you.

Interviewer

OK, sir. If only with Earned Value Management indicators, let us say with CPI, with schedule variance, many people do not understand, especially in accounting then in marketing, with EBITDA as a measure of profitability that is common in financial reports, do you think it helps them understand or what, sir? Please.

Interviewee P

Er, today friends at, especially in accounting, sir, yes, we have been working together since the beginning. Um, we do a weekly review, sir. Eh, and we get information about EBITDA and sales achievements, then what are the targets from our RKAP so that our EBITDA will appear negative or positive in the end? That can do it, sir, yes. It is just more reporting, I see. And the expected things do not appear, such as alerts for other supporting functions. And right we should also find out why he did not reach his EBITDA. We should be looking for the root cause, the deepest one, so we can fix it. Well, this often does not happen, sir. So weekly it happened, a review took place, but there was no explicit action from the meeting results, so the following week when we reviewed the position again, there did not seem to be any significant changes like that. It is like we are just listening to reports. After that, it just goes by. So, this is what I think is the problem. We should have acted on what was reported that we had to follow up on to improve the condition at that time so that it was not at the end that we found out that, oh, it turns out this project made a loss. So, we need to cooperate with them so that we know exactly where we are every week, and we must know what to do to improve the situation that has occurred so that we do not lose more from projects that are currently running. That is, it, sir.

Interviewer

With Earned Value Management, we can see the project's condition at any time, but the EVM indicator has not shown profitability. So, by attaching EBITDA or profitability to EVM at any time, not just daily, ee, the whole organization can see it so that whatever difficulties there are in the field, they can immediately assist the project management. And agree, as you said earlier, that they are not just watching. Still, they participate in helping with the difficulties in the field. Then with the profitability indicator of the project, at any time, it can be seen by the board of directors, ee, do you think there are any benefits or not?

Interviewee P

Er, especially useful, sir, in my opinion. So, the directors can also see the performance of the directors, right? It should be monthly. So, every month, gentlemen in the directors can see the performance of each project. These efforts also include communication that makes, um, later, there will be policies taken by the board of directors when we experience problems that we cannot solve under specific circumstances related to working capital. So, working capital can be my biggest problem because it will impact procuring payments for these materials, so they arrive on time. And in my opinion, this is especially important, sir,

to be opened to the BOD, to be seen by the BOD so that the BOD does not only see the results but also takes control of the travel phase over months, that is it, sir.

Interviewer

If they see the display using IT every day, it can work, right? So indeed, the meeting can be monthly, but he can see an act and come to the site with IT to see the performance of each project. OK, sir. Er.

Interviewee P

Maybe, sir, if we meet and the BOD sees it, at least there will be an interaction like that, sir.

Interviewer

Yes sir. Yes sir. Yes, Then, what do you hope with the integration of EBITDA into Earned Value Management for COMPANY projects, sir?

Interviewee P

I strongly agree, sir. It is excellent, sir.

So, the management, all those supporting can see what is going on with ongoing projects, and that can uh, what is it called, make a correction or a directive that makes this project run the way we want it to. Sir. I strongly agree. It would be excellent if we could do it.

Interviewer

All right, sir, thank you for your time and permission to be interviewed. Especially useful for the COMPANY and its improvements. That is what I say. Please let me turn off the recording, sir. Yes.

# INTERVIEW Interviewee Q

Interviewer

Good afternoon, Q; how are you?

Interviewee Q

Yes, thank God, Sir.

Interviewer

Yes, good. We will interview for a case study on how COMPANY Indonesia implements EBITDA integrated with Earned Value Management for project management. Well, COMPANY is a project-based company. In English, it's Project Based Corporation, so if you want to increase Company's EBITDA, that's what you need to improve on the project. Then project management is project management managed by COMPANY. I think the other thing that is the same as the Work Breakdown Structure is the WBS, which is also implemented at COMPANY. Then based on your knowledge, there is no problem to answer this. So, for the question, we use the 7 scale; yes, the name is 7 on the Likert scale, but scale 1 strongly disagrees. So, if there is my statement, if you strongly disagree, you will choose a scale of 1. Scale 7 strongly agrees. So, if a scale 2 does not agree, 3 somewhat disagrees, 4th is neutral, 5th somewhat agrees, 6th agrees, 7th strongly agrees.

So, here is a statement that the first statement said that EBITDA is the proper measure of project management profitability. Because EBITDA is a measure of operational profitability before paying interest, paying taxes, paying amortization, and depreciation. And the value of EBITDA is not affected by interest rates, is not affected by taxes, is not affected by amortization depreciation. So, this is pure Profit that is not affected by it, so the efficiency and productivity of this project influence Profit. Do you agree that EBITDA is the proper measure of profitability for project management because it is not affected by interest and so on? Your opinion?

Interviewee Q

Number 6, Sir. Agree

Interviewer

Number 6, Sir. OK, Agreed, Sir Yes, Sir. Good. Second, EBITDA is the proper measure of profitability in project management. Because it is entirely under the control of project management, starting from procurement, logistics, then workshops, to delivery. Your opinion?

Interviewee Q Agree.

Interviewer

Agree. What number, Sir?

Interviewee Q 6.

Interviewer

Number 6, OK. Then the company can integrate, and COMPANY is currently integrating this. In a way, EBITDA, or earned value management, is the first time the authorized budget is determined as income. So, the budget from Indonesia Power is Company's revenue plan. Then we insert first the name EBITDA, EBITDA Margin, then the rest is costs. So, the cost is not the same as the budget. So, if it's the same, then it's spent, and there's no profit, right? What's your opinion, Sir? Number?

Interviewee Q

Oh, that's it, Sir. Because it's been in production all this time, the budget sometimes doesn't go down. Yes. Those costs are not going down. So, I have conveyed to you that the budget and the costs should be distributed to the project management; for example, what has been scheduled so far has come from PMO or PPC, Sir. That's sometimes, the people at the top level should understand their responsibility to distribute the schedule and drawings to the project management timely.

Interviewer

The budget and project plan should come down, Sir.

Interviewee Q

It should have come down.

Interviewer

So, we talk "should," Sir.

Interviewee Q Agree

Interviewer

Yes, so EBITDA should be used to control profits, and we should assign the person in charge of the EBITDA. Yes, that's it, Sir. Your opinion?

Interviewee Q This

Interviewer Please, Sir.

Interviewee Q Agree

Interviewer

EBITDA can be integrated into earned value management. Do you agree or not?

Interviewee Q I Agree, Sir. Six.

Interviewer

Six, OK. Then next, companies can integrate EBITDA into earned value management. The trick is to set an EBITDA margin. The project EBITDA margin is then broken down into each WBS EBITDA margin level 1,

level 2, up to the work package level, where there are EBITDA margin numbers. Do you agree or disagree?

Interviewee Q Six

Interviewer

Six, Sir. Good Then, the company can benefit from using EBITDA to measure profitability and project management profits because EBITDA is the usual operating Profit in the financial statements. Because so far financial people don't know how much Profit it is.

Suppose we look forward to the profitability report. But by combining it with EBITDA in EVM and project management, people can see the project management profitability; they can see. What's your opinion?

Interviewee Q Seven.

Interviewer

Agree. The numbers?

Interviewee Q Seven, Sir.

Interviewer

Seven Sir. The company can benefit from using EBITDA as a measure of profitability or project management benefits. Because then the project manager knows, the logistics person knows, the procurement person knows. So, they can maximize their EBITDA because, you know. Your opinion?

Interviewee Q Seven.

Interviewer

Seven, OK. Strongly agree. Then to increase the support of supporting units for project management, the project manager needs to report actual EBITDA achievements compared to the target to the supporting units. We also report on logistics, procurement, engineering, and finance. Do you agree or disagree?

Interviewee Q Strongly agree.

Interviewer

Finally, to make it easier for the directors to provide timely assistance or strategic decisions to the project manager, the directors must also be reported. What is the EBITDA of the BMPP project, and how much is the EBITDA of battleships? So that they can make decisions that sometimes the decision is not owned by the project manager. According to you?

Interviewee Q Strongly agree.

Interviewer

You strongly agree; fine. Structured questions are complete.

Next, kindly tell me how you hope this EBITDA has been integrated into earned value management, integrated into project management, and everyone can see the project's profitability. What are your hopes, Sir? Please sir?

Interviewee Q

Thank you, Sir. So, this is it, Sir; it's often touted to strengthen your plans. But when it's running, sometimes there must be a delay at the start. It happens a lot. Not necessarily late, but often. So, in the production schedule, it is always pursued. So, in the beginning, starting with the design, from design to PPC, sometimes they don't know which items should come first.

Interviewer Yes

Interviewee Q

or items that should be installed first are not running.

Interviewer

Yes, and maybe they don't know how much the company loses. That's right, Sir. So, there are no numbers, no rupiah, so yeah, it's normal. What do you say, Sir?

Interviewee Q

Yes, I dug into SVP's words; this needs to be fixed; this is the front because it's been in production so far.

Interviewer

If the production is good, that's good. I can build all kinds of ships.

Interviewee Q

Commitment to pursue it though hectic, but still able to finish on time.

Interviewer Yes

Interviewee Q

So, the hope is this, for example, if the portion of the burden is for the application of EBITDA, maybe in the field, it can be.

Interviewer

Not only in the workshop

Interviewee Q Yes

Interviewer

But it's also in procurement. That's it.

Interviewee Q

The procurement value is 60% of the costs; should prepare it upfront.

Interviewer

That's right, that's right. So, 60% preparation is essential, Sir. Procurement logistics must also calculate its EBITDA. So, if he is late, how much will his EBITDA decrease? If the people are more diligent in planning, their EBITDA will increase, right, Sir?

Interviewee Q Yes

Interviewer

That's the impact on the workshop. Those in the logistics, procurement, and finance offices know that implementing their daily tasks impacts the EBITDA of project management. What are your hopes for the supporting unit, Sir?

Interviewee Q

Yes, it must change, change following this one. If we refer to EBITDA, which calculates Project management Profit, it must be in front. For example, waiting for half a day; if it can work in two days in the field, that delivery delay must be known.

Interviewer Yes

Interviewee Q

For example, if there is a revision of the drawing in front, Sir. Revision of the drawing takes half an hour; if implemented, it can be finished. But his work in the field can take up to two days.

Interviewer

If the logistics are late, can the work in the field take longer, Sir?

Interviewee Q

Because they are related, Sir. Suppose the one you are waiting for hasn't arrived; in the end, the rear one can't be installed either.

Interviewer True, true, true

Interviewee Q

Back off everyone

Interviewer

There is a work sequence, Sir; there is a bottleneck like that.

Interviewee Q the bottleneck

Interviewer

So yes, it's hampered. OK. Then, for procurement, logistics is very disciplined. Following the WBS, following the plan, it's easy, Sir. To carry out your experience for a long time. The production is not a problem at PT. Nutmeg. So, what needs to be aware is not only in production but also in procurement and logistics. What do you hope for that at the company ?

Interviewee Q

My hope is from management. Because this is for the application of EBITDA, the BOD has been inflated, Sir. So, for production, it has been proven, but for support from supply chain, procurement, from design, this begs for a change.

Interviewer

the company needs a Cultural change, Sir, yes.

Interviewee Q Yes

Interviewer

Change the way you work.

Interviewee Q

Right, Sir. So please, maybe later, Interviewer will convey the possibility to BOD. The hope is that it will be ready when it is in production. And as for what I conveyed to Interviewer, if there are pictures and material in the production, that's it.

Interviewer

Go, see, act to understand the job in the field.

Interviewee Q

Achievable. Moreover, it's been neatly arranged; maybe even running can be added, Sir.

Interviewer

Yes, yes, Yes So if the EBITDA project management is procured, it will be fulfilled by the procurement person. Then the logistics people also comply. Engineering support also complained. Then it will be smoother, Sir, for the implementation.

Interviewee Q

It will be smoother, and what is conveyed in EBITDA related to the Profit earlier will be very much achieved, Sir. Its more job accomplished.

Interviewer

Will be very achieved, yes. What are your hopes for implementing EBITDA in this project management? What are your hopes like?

Interviewee Q

Ugh, Sir. This EBITDA integration is what I will propose later. So far, we have run out of JO, finished JO, and run out of anchors. We never know, for example, one block, sir; yes, A's work with Man-Hour is like that.

Interviewer

Never know how much the Project Profit is; I don't know either.

Interviewee Q Do not know.

Interviewer Do not know.

Interviewee Q

Suppose we are limited to 300,000 person-hours, and these resources must be used to finish the project.

Interviewer Yes

Interviewee Q

We in this field can also be sure to plan.

Interviewer

Yes, but what can you do?

Interviewee Q

What can you do, Sir?

Interviewer

Yes, so by controlling costs better, how much Project Profit will that be, right, Sir? You never knew before, did you?

Interviewee Q

Never know. For example, if the quota is 300,000 person-hours, I get it done with 250,000 person-hours, and when it's finished, there's 50,000 left. It's a matter of pride for the Team.

Interviewer

That's right. So, if you know the EBITDA target in every business process and can achieve it, your subordinates are also happy, Sir.

Interviewee Q

Yes, but during this time of EBITDA integration, sooner or later, we will achieve our target too. Yes,

Interviewer

Yes, last year, sir. Later it was a gain or a loss. But with this, you can find out daily. If the work is like this, the result is this; if the work is like this, the result is this. So that you can improve the way you work, Sir.

Interviewee Q

This EBITDA application to the project adds to the motivation, The culture may change later.

Interviewer

Yes, yes, yes. Then, OK, what else do you expect from implementing EBITDA in this project management? What are your hopes for the company ?

Interviewee Q

Yes, so planning is strengthened, apart from materials and results, Sir, so planning is strengthened so that each line can control it according to their respective parts.

Interviewer

According to the scope, yes Interviewee Q

Yes, each has its scope, which is also related to the installation schedule; that's the hope that those brought in will suit you, Sir. So don't let those who haven't, those who have waited a long time, come first; those who want to be installed haven't arrived yet. The hope is that it will be included in the planning.

Interviewer

Yes, yes, yes. All right, Sir, thank you very much for the interview; if you are willing, I will also convey this to you later; here are the case study results. Maybe it can inspire friends to know what the best work for the COMPANY is and provide better welfare, Sir. There is another message, Sir. The last one, please, Sir

Interviewee Q

Yes, Sir, for example, later, we really can't just turn our hands back, right? It's the culture, Sir. Yes. Indeed, it may already be taken root, but by changing little by little, it will be achieved later for Project Profit. So, we changed together.

Interviewer

Yes Sir. OK, Sir. Is there anything else, Sir, that needs to be said?

Interviewee Q

Yes, Sir, that's all for the time being earlier, for those that are being produced, once again, if the material schedule is 100% planned, Maybe, maybe delivery on time can still be very open.

Interviewer

OK, Sir. OK, Sir. Please allow me to turn off the recording, Sir.

INTERVIEW Dr. Interviewee R

Interviewer

Good morning, Dr. R, thank you for your permission to do this interview. We will use this interview as evidence from case study research regarding the application of EBITDA in Earned Value Management project management at COMPANY. Then there are two sessions; the first is a structured question and answer, and the second is unstructured. For the latter, we will use the seven Likert scales. Score one, scale one strongly disagrees; then scale seven strongly agree, including number two disagree, number three disagree, number four neutral in the middle, then scale five agree, scale six agree, scale seven strongly agree. Can we start, Sir? Yes, to the question.

There is a statement that EBITDA is an appropriate measure of project management profitability because EBITDA is operational profitability, operator profit before interest expense, taxes, depreciation, and amortization. Therefore, EBITDA is not affected by the amount of tax interest, depreciation, and amortization, and EBITDA is only affected by project management efficiency and productivity. Your opinion number one to seven, from strongly disagree to agree strongly?

Interviewee R

Permission, Sir, I totally agree, Sir, seven points, Sir.

Interviewer

Strongly agree, Sir, good Sir. Then the second, EBITDA is the proper measure of project management profitability because EBITDA is entirely under the control of the Project Manager and the team, from start to delivery and warranty; what do you think?

Interviewee R Strongly agree, Sir.

Interviewer

Strongly agree, Sir. Thank you.

Third, the company can integrate EBITDA into Earned Value Management by first setting revenue; that income is the authorized budget the project owner provides to COMPANY, we consider it as a revenue plan. Then the COMPANY can determine the EBITDA plan to determine the plan costs, so the cost plan is the income plan or authorized budget after deducting the EBITDA plan. Your opinion?

Interviewee R

Strongly agree, Sir. Minus the EBITDA plan, there will be the project management costs there; yes, I Strongly agree, Sir.

Interviewer

So, the cost plan is not an authorized budget; if it is the same, there will be no margin, right?

Interviewee R

Yes, Sir, I strongly agree, Sir.

Interviewer

OK, Sir. Then next, the company can integrate EBITDA into Earned Value Management by setting the EBITDA margin of the project, then the EBITDA margin is divided into each work Breakdown Structure and Work Package, so everything is derived accordingly, then the integration implementation is integrated.

EBITDA to EVM, according to you?

Interviewee R Strongly agree, Sir.

Interviewer

Both strongly agree, then the company will benefit from using EBITDA as a measure of profitability in project management because EBITDA is a standard operating measure for financial reports. Thus, company management can understand and know the project's profitability because the project management has an EBITDA indicator. According to you?

Interviewee R Strongly agree, Sir.

Interviewer

OK, Sir, I Strongly agree. Then the company can benefit from using EBITDA as a measure of project management profitability because by doing so, the project manager and the team know their EBITDA target, so they can maximize the profitability of project management because they know the target, Sir.

Interviewee R Strongly agree, Sir.

Interviewer

Strongly agree, either. Next, to increase support from supporting units to project management, the Project Manager needs to report the actual achievement of EBITDA compared to the target EBITDA to the company's supporting units so that they know and can support. Father's opinion?

Interviewee R Strongly agree, Sir.

Interviewer

OK, Sir, I Strongly agree. Point eight, to make it easier for the Board of Directors to provide timely strategic decisions to project management, the Project Manager needs to report the actual achievement of EBITDA and its targets so that the BOD can provide direction and strategic decisions which are often outside the Project Manager's authority. Father's opinion?

Interviewee R Strongly agree, Sir.

Interviewer

All right, Sir, I Strongly agree.

Interviewee R

Sir, the answers are all extreme, Sir.

Interviewer

Oh yes. All right, Sir, the structured interview is over, then please allow me to tell you about the free benefits of EBITDA being applied to Earned Value Management to manage project management for COMPANY. Please, Sir.

Interviewee R

Thank you, Interviewer, for asking for permission; I will convey the implementation of EBITDA and Earned Value Management at COMPANY, only then will we combine the correlation or relationship, Sir. So related to EBITDA and Earned Value Management COMPANY is the fundamental basis for making project management and subsequent performance assessments. So, these two things must be set up first, Sir. I want permission later for EBITDA. besides being related to the financial aspect of the horizon that we are reviewing, I will go into accounting a little, then Earned Value Management, I am in the current position that you have explained, or we will study it together, the position of the discussion. I will go into the EBITDA first; if it is related to that aspect, we have often studied with my father, I will go into the accounting view, so the EBITDA calculation methodology is related to the application of accounting in COMPANY. As you know, at COMPANY There are two types of project management or the nature of the project, so there are long-term projects and short-term projects. So, the long-term ones are like making helicopters, which is all the long term, where more than a year later the short-term ones like Interviewer are currently guiding us, related to the Aero-Structure program which makes airplane or helicopter parts directly we sell to the end customer. Related to the nature of the project, where the horizon is not short, the long term, there are also differences in the accounting records, so we use the long-term one, which is called the completion procedure. So, the completion rate is a periodical; then, we value money, and we value it as the basis for recording revenue or sales. Then the short term is the recording when the achievement process has been submitted, or the goods and services have been submitted; then, we record the invoice as income or revenue. As is known that when we calculate EBITDA, the top-right is revenue in the recording process because the exposure is revenue, so it is influenced by the two methods of recording earlier, namely related to long-term projects that are following their progress, then related to short-term projects that are less than a year old which is indeed recorded during the delivery and invoicing process carried out to the customer we are. So, in this position, because of the two records earlier, those related to the recording of the accounting view, which is a long-term project, will need tools in accounting, use tools. Permission, Sir, I will stop here regarding the EBITDA horizon, which is related to the accounting view, namely the emphasis on the other top, which is related to the revenue recording or the revenue reconnection, the recognition of the venue for the bottom one, I think the finance and accounting are the same there so. Then for Earned Value Management, as you described earlier, this is a tool for my understanding so far, namely to find out the performance or progress of a project; this one is due to packaging, so we can see not only related to the physical but also related to commercial aspects, in the calculations in the value management. not only how far the schedule is or where the technical position is, but "Earned" here is so complete that commercial can enter what percentage of the totality of the project is because it has been described in the WBS (Work Breakdown Structure) in detail so we can find out

comprehensively to what extent is the progress of the project so that we can find out later at which points are related to achievements and at which points this project is compressed following the contracts we have agreed with our customers. Even earlier, you said at the beginning that it is related to project and project management, where is it? If the project starts from the goal or dream, it is designed to the end; if there is project management here, starting from the contract is signed until our obligations are completed, our obligations are completed. Where is the warranty position? We have managed the project properly because we promised the contract related to product support. With regard to EBITDA and Earned Value Management, I have previously conveyed the permission for EBITDA; there is an accounting horizon that I have included here, one that for recording EBITDA with the methodology that was ported earlier, it recognizes compression persecution for the long term, so the recording can be perfect if Earned Value Management is carried out well because, with good Earned Value Management, we can find out the stages of the level of completion of a project. It is the same as knowing the stages of completing a project, so you can draw it later to the accounting at that stage; how much revenue is that? So, if Earned Value Management is not conducted, then the recording of revenue for the long-term project cannot be recorded perfectly. The EBITDA calculation cannot be done perfectly. The data is misleading; the information will also be misleading. The final decision will also be misleading regarding the same project itself to support the project or later in the system of directors related to where the company is going or how small this project will go for completion. So, in this position, the benefits of Earned Value Management are related to validating the EBITDA calculation itself. It can be said that if Earned Value Management is not carried out, then the recording or calculation of EBITDA, especially the topline here, which is related to long-term projects, is bound to have low reliability, but if Earned Value Management is carried out, then the record of the revenue automatically reliability is also high. Well, because Earned Value Management is in this context, I beg your permission, Sir, to use it because of its relation to finance and accounting while equating the perception I have understood so far.

Usually, it is the same if we subtract the profit from the budget in finance, so the budget is also with that profit. So, the margin is OK; that is how we separate it. So, when it comes to finances, here is the budget cost, the total, from the contractual signature to the warranty, so what is the total like? So, we can define this well in Earned Value Management. Then we can also take pictures and products for three years, then in the first year, how many two, how many three, how much is that? Then when compiling EBITDA, our target EBITDA can also be calculated because it is related to recording revenue in EBITDA earlier based on the tax rate. We can measure the project level with the cost earlier, so in EBITDA, we know the target venue and the target boarding house with good precision. And then also later, at the time of the actual calculation there, we have actual revenue and actual cost with good reliability so that when measuring performance, it is related to achievement, namely related to the budget until the realization there will be recorded well, or the reliability is good, so the position in there, so that later for the preparation of information and decision making, the position is not misleading.

This connection with EBITDA Earned Value Management is related to the self-development of EBITDA for long-term contracts at COMPANY, there is a spirit or fundamentals. Earned Value Management is wrong, EBITDA will be wrong, Sir, which is his position. Please, sir, permission in this position. Does it need discussion up here?

Interviewer

Then I said earlier that the benefits of EBITDA in Earned Value Management, then and because it has been passed down to the WBS, at any time COMPANY can take pictures of productivity gains and their EBITDA;

the condition is that EBITDA is allocated in each WBS, and that's fine until Work Packages and Earned Value Management are done to perfection. It is fascinating because not many elaborative people get there because they must understand how important it is to understand production to make it enjoyable. Now the benefits of COMPANY with the integration of EBITDA in Earned Value Management, where previously Earned Value Management did not know the profitability; we were then there until the EBITDA indicator, which can be photographed at any time; what are the benefits, Sir?

Interviewee R

Yes, Sir, thank you, Interviewer. Permission, I am going to the second topic. So, for the first topic earlier, I apologize for using Earned Value Management for EBITDA calculations. So, to build the data and information we linked. Now, how about using EBITDA in the tools in Earned Value Management? What exactly are the tools for taking pictures, Sir? What did you say earlier about taking pictures? As you often tell us, EBITDA is here to engage the fundamental idea that we must be able to differentiate the level of decisions, right, Sir? I have observed all this time, So, that there are unquote decisions at the project level, and then there is the corporate level, so they are. It is automatic if the decision has to do with accountability for power, allocation of resources, and so on it goes there. EBITDA here is related to earlier decision-making; this is a sorting tool. So which one is at the project level position, and which one is at the corporate level position, so here we also do not get mixed up about which one is at the decision level, which is something different. The hierarchy is manageable in the hierarchy, and which are not manageable in the hierarchy. So, an EBITDA is also a localization tool when we talk about decision making in this case, in the decision-making process. Then the second one also attracted me. Thank you very much, Interviewer, while at COMPANY really helped us to provide a total understanding of COMPANY that a company can run well, operate well to achieve its vision if there is a view, the vision is a commercial view, so the point is a company with a core business, quotation marks, it must be able to create value, that's because there is a commercial here, the value must automatically be measured by money, the point is there.

It is related to other finances. It is only a bridge, so it is bridging; so, for example, if you are sorry for allowing a company to exist, how come it can create value? Then the value there is from the core of the business, that's when it's honest money, then roughly it's easy, for example, that revenue minus the cost in the project or the core business, there must be a surplus called whatever there is, so we use the automatic EBITDA approach in that position, Sir, yes, we use the EBITDA approach. So if in a position maybe in specific periods where the EBITDA is negative like that, right, because the EBITDA there is the closest to the operating cash flow, right, then that reflects the incoming and outgoing money related to the operating project, then we just do bridging externally, looking for a loan or looking for something like that, and trying to be healthy is called bridging there as a bridge like a highway, the road itself and the bridge should be longer. If you have a problem, the company will too. Then the core project must continue to be in surplus. Then if there is a negative or sorted, we will bridge it from another place, which is automatic in the short term. The bridging project must be controllable, plus the cost, right, Sir? That is the position there. So, thank you, Interviewer, for the understanding you conveyed at COMPANY, it makes it easier for us.

How do we achieve the vision achieve the objective periodically? The basis is based on the project; the basis is on the core business, not in a bridging position to externals in such a way or always what you always tell us is not related to Capital making continuous capital injections, so this is very good, Sir in the capital structure on the right side, we emphasize that we do not prioritize our balance sheet on the right side, but what about our assets we rotate it in such a way that the project runs and that's it, Sir, here.

That is an exceptional understanding, Sir; what we feel now with that position is the main thing after you; this understanding is now what tools are to help, and that is why the tools to help here are EBITDA, which we have discussed so far. What is helping him now is regarding EBITDA here; if we see that it is related to project management and contracts until a few earlier, we automatically use a sizeable objective level; there are three, right? So, the first is delivery on time according to the contractual terms, then the quality is also according to contractual terms. Then the cost is automatically in our interest to keep us in a position that follows the initial standard or is smaller than the standard so that the margin will be better. Well, regarding EBITDA here, we will guide you through the three steps above will guide you well because, with the position of most companies, there are long-term contracts where Earned Value Management is mandatory for calculating EBITDA then. EBITDA will guide you in the related field. We are managing or running the project. So we already have, for example, related to being moderate, not having at the time of building, building performance targets from Earned Value Management following the stages or project performance with management tools according to the stages, so here we also have to EBITDA what is the timeline we arrange, the time we compiled the line at the time we compiled the EBITDA timeline, so here we also have to know the revenue as well as the timeline that we prepared in detail earlier. Because the revenue is a long-term contract, in this project, we must set a target cost periodically because to calculate the revenue earlier, we also arrange the costs according to the timeline that is detailed.

Well, we can arrange the costs for the activities, we can also arrange them with periodic details of the activities. So, in this process, when we compile activities and periodic, to compile periodic costs, we need a detailed Work Breakdown Structure-activity to run the project with a detailed timeline like that. So, from there, we calculated the cost based on the WBS activity earlier in every detail, along with the timeline. So, in this position, revenue, and activity-based costs, for that EBITDA can be arranged in the targeting position. Well, then, when the WBS is realized, the WBS that has been arranged is automatic; what are the activities like when running it, then what decision does it need to make so that the WBS can run smoothly, both related to the allocation of resources and in the position of problem-solving if it's needed at every periodicity of the activity which has been targeted every time earlier.

The activity will be automatic later, and there will be our output, right, Sir? Just allow me to go global. Even the output here is automatically related to the output of goods or services or the value there, then added with quality according to automatic requirements following the requirements for goods and services with the required quality. Then the third is how much it costs to conduct this activity with the required output and the cost. After that cost, then we can compare it with the target earlier, then we can see there is a deviation, and the deviation can be a reasonable deviation or a harmful deviation or the same position, Sir, which is the result. So, if the deviation is reasonable, how can we maintain it later? Can we run the project earlier with the required quality? Then, in this position, delivery is faster than the planned schedule, costs are cheaper than the planned costs, and margins are higher.

than the target margin.

If the deviation is negative, being late is not late means it is late, or the quality is worse than the plan; the cost may be higher at that stage.

So, for future activities, the process of delay recovery and recovery quality, how to optimize it related to the existing costs. So, in the context of the optimal cost, we will contain two costs: the cost of recovery and completing the project until it stops. So, with the project going forward until it stops running out, how much will it cost later, how much will it cost, and what will the recovery be? So here we are, optimizing it. How can the project maintain margins like that? Well, the consumption is different here. It becomes a tool for total project management here. An automatic short-term horizon assesses periodic short-term

performance, serving as a warning performance report in Earned Value Management. What is the quality of this project? Later assisted with the tools in Earned Value Management related to quality, and then the same profit margin is still maintained or not, or is the EBITDA still maintained? We will use EBITDA concerning the target and the total project later. Now, we are anticipating that if it is still on track, it means that the project is already running well; if it is not on track here, then we think first about what the recovery should be, then what is the relationship between the cost record and the total cost of the project, because later related to margins. That is my brief conclusion; please, Sir, if you need further discussion.

Interviewer

So far, EBITDA is understood by financial friends, while non-financial friends such as the Project Manager, then his team, and the engineers do not understand that we introduce EBITDA to non-finance at COMPANY in managing project management, to what extent are the benefits, Sir?

Interviewee R

Thank you, Sir. My permission will go global and then go into details, Sir. Which is it? To be a good company, we must manage commercials well; one of the good commercial measures is good corporate EBITDA. Corporate EBITDA is good because it is close to cash flow operations. So, EBITDA is good, related to cash flow operations; if it is linear, then the EBITDA calculation is perfect. That is good because it is close to cash flow operations, which is from a calculation standpoint, from a cash flow operation point of view, good EBITDA is good. I borrow the term, Sir, all this time you have said that means the deposit is good, so in this position, as a corporation, it is this corporation. It is making good deposits, and the point is that it goes to the shareholders, right, Sir, or existing shareholders; that has the effect on the shareholders like that, so the deposit is good. We know that we are cascading EBITDA at the corporate level, a collection of EBITDA- EBITDA projects we are working on, right, Sir? So relatively, the first EBITDA project exists as a cascading; the cascade of corporate goals is reduced to a figure of the objective under which the purpose of the corporation earlier, the objective of the corporation to cascade into the goal of the organization, what is the permission, the purpose of the organization, the funds yesterday, you have built an excellent EBITDA project and EBITDA function to read the automatic support function in EBITDA, it's just the cost, isn't it, Sir, if it's there it's complete That's it, then support will be attributed there. Some are in a permanent position at the corporation, right, Sir? Which was the first one as a cascading tool; Sir, this is good there. One of the successes of cascading is if you understand well what is cascading. Well, with the understanding that the EBITDA of this project is derived from the corporation's EBITDA, we have the same understanding as PT from a strategic point of view. The company that the role of the company is so that we have good prosperity, then EBITDA must be positive and built from EBITDA-EBITDA. The project means that if the corporate EBTIDA had to be positive so that we prospered together earlier, the EBITDA of the project had to be positive, so starting from the targeting, it was positive, then at the time of implementation, how to make the EBITDA positive according to the target, one pack. Then secondly, there is an interesting understanding related to porfolio management, Sir. So, it is related to the product cycle of the initial ground majority in the client; it has its own EBITDA character. When we had each EBITDA here in porfolio management during the checking process, our product initials, for example, N-219 aircraft, were also root; for example, at this time, it is quite difficult to find a root, Sir, at COMPANY, most of them have the majority, and Jackline, the position there. Well, the initials are usually negative, right, Sir? In the majority position, there are lots of cascos, so EBITDA is there usually overflows in position there. Then on this client, it can start to think or be negative again because it is indeed in the terminating position. Well, this is a tool there,

related to structuring EBITDA quotes according to the Broad cycle, it becomes important, and now friends understand, Sir, how to be positive EBITDA when we can get EBITDA in proportion, Sir, which permits because of the combination of the EBITDA project porfolio product, so we can now pinpoint the initials of the majority group and the decline related to the EBITDA of each earlier, the first one must be surplus or negative to be tolerated, sorry everyone should ideally all be surplus, but if it is negative according to the site earlier, that's what we can know downstream one. Then the second time, EBITDA was collected in all cycles of the product porfolio. If the proportion demands that here healthy ones have to be positive, how was collected earlier also positive, although here sometimes we also cross-subsidize, for example, with the majority of products, the same customer, it could be We will sell this product to the same customer, some will lose, and some will gain, but we cross-subsidize the manifestation in EBITDA, which can be high or thin or negative EBITDA equal to the moderate EBITDA of the three EBITDA we mix so that the EBITDA output is optimal for that one consumer. So, the second one here has benefits, Sir, for porfolio management, for how to support the corporate EBITDA in each of them; by knowing the character, we know what EBITDA should be like and what it will be like at the time of implementation. From here, Sir, thank God, you can lead well with the max EBITDA, so it's OK, EBITDA can be from projects that have positive totality, for example, Sir, but because it's not maximum related to capacity, we'll have a gap, when we go to the corporation, because there's an idle capacity cost, which automatically goes beyond the existing EBITDA, right, right, at this time the total idle corporation We were going to enter the EBITDA of the corporation, so we will know that there is unused capacity. This capacity planning is how it manages exceptionally long marketing, automatic long-term market, and short-term sales to fill this capacity. This porfolio management is the objective of EBITDA three; this is interesting, so we have related the management of the porfolio's EBITDA according to the cycle, then the third management is related to the output to corporate EBITDA, which is related to the capacity to make that is needed there. This third permission is still related to macros. Then the fourth one is already in a micro position, so it is for management related to the project itself. That is because, in Earned Value Management, there is the point of measuring all existing activities, so here we go into values. Sorry about the Value Chain, which is remarkably interesting. create a value chain; it is a value chain precisely because there are words value than its position. Much content, namely dividend activities, must contain added value. Indeed, there are non-value-added ones that cannot be avoided, for example, setups, moving materials, and others; namely, how do we pass them on, Sir?

Well, in this fourth position, then with Earned Value Management, there is management in the WBS, then in there is activity or value chain management there so that we can process the value chain properly. Well, as long as the details are in the project earlier, then later we can also link it with the management of the value chain at a macro level, namely the relationship with the capacity make that you mentioned earlier, so the capacity makes to do with the size of the building, for example, the building does not add up to the total capacity first, Sir, for example, the building is like that, with the position there, for example, we still have problems managing the building, for example, many don't idle automatically, those that are there are non-value added. So, we can localize these projects; we move them so that the moving material does not have a lot of non-value-added activities, so we can place them in one location so that the moving is not too far away, so the cost is not too far. Then also lastly, Sir, because there is also a WBS, WBS needs sources; this is for spending resources, so it needs it there first, automatically human Capital, so processing human Capital, for example, is related to the requirements of the competencies needed, when it is needed it becomes important So, the requirements there are following the timing, human resource development is also following the timing, the certification is also following the timing, so the terms don't have to be

behind the requirements, we have prepared them up front so we pay unemployed people, for example, so that's the position, Sir, that's my permission conclude, maybe there are many shortcomings, please Sir, complete it.

Interviewer

All right, Sir, I think it's complete, and I think we need to share this interview with COMPANY friends so that they also have a sense of belonging to the progress of COMPANY, I think it's extraordinary, profound, and very broad; thank you for the permission, Sir; sharing share knowledge in implementing EBITDA in Earned Value Management, Sir. Please let me turn off the recording first, Sir; thank you.

INTERVIEW . Interviewee S

Interviewer

Good afternoon, madam. Let us start the interview with these questions. The questions and the questionnaire are needed to write down the case earlier regarding the implementation of EBITDA in the project management of the company. We use seven scales. Scale one strongly disagrees, scale seven strongly agrees if two disagree, three somewhat disagree, four neutral, five somewhat agree, six agree, and seven strongly agree. That statement, EBITDA, is a proper measure of project management profitability. Because EBITDA is a measure of operating profitability, which does not consider interest, does not include taxes, does not include depreciation, or amortization. So because interest is a financial matter, financial taxes and depreciation are the affairs of the directors and so on. And the value of EBITDA is not affected by tax interest, depreciation, and amortization. The EBITDA value is only influenced by the efficiency and productivity of the project. If the project is efficient and productive, then the EBITDA is good. According to you, this statement agrees or disagrees with a scale of one to seven.

Interviewee S

Seven, Strongly agree

Interviewer

Strongly agree, good madam. Then point 2, EBITDA is an appropriate measure of project management profitability because EBITDA is under the control of the Project Manager, while interest, tax, depreciation, and amortization are not under the control of the Project Manager and the team. What's the scale, Mom?

Interviewee S

Number two, EBITDA is the responsibility of project management.

Interviewer

So for that, interest is not the responsibility of management.

Interviewee S

That's Strongly agreed.

Interviewer

Strongly agree well, Number two Strongly agrees.

Point three; companies can integrate EBITDA into earned value management by setting revenue as a budget, the budget or sale is from revenue, then inserting EBITDA first, then we find the costs. Hence, the costs are the budget minus EBITDA. Agree or disagree.

Interviewee S

Yes, the budget is.

Interviewer

Cost is budget minus EBITDA with profit.

Interviewee S Yes, that's right.

Interviewer

All right, what's your scale, Mom?

Interviewee S Scale seven.

Interviewer

Scale seven, thank you. Then the company can integrate EBITDA into earned value management by setting the EBITDA margin of the project, all of which are broken down into EBITDA margin for each WBS for each work package. So in each WBS, there is an EBITDA target so that implementers know the EBITDA target. Agree or disagree?

Interviewee S

Agree so that each member can control the other; strongly agree.

Interviewer

Strongly agree. Point five, companies benefit from using EBITDA as a measure of Project Manager profitability because EBITDA is a standard measure of profitability in financial statements. So if there is no profitability in financial reports, people outside the project don't know the profitability. Now with EBITDA in each project management, company management outside of project management can understand the profitability of the project management. Right or not, madam?

Interviewee S

That's right, and you have to know everything so that everyone is aware, controlling each other so that it is the most effective.

Interviewer

So, question number six agrees with point seven to increase support from supporting units to Project Management. The product manager needs to convey and report the actual EBITDA target, whether it has been achieved or not so that the supporting units can help better. Agree or disagree?

Interviewee S

That's right, that's right, I Strongly agree.

Interviewer

Then the eighth, to make it easier for directors or product directors to make strategic decisions or provide timely direction to the project manager, the project manager needs to report the achievement of EBITDA and its targets to BOD Because not all strategic decisions are in the hands of the project manager. So if the board of directors knows, he can promptly provide direction and strategic decisions.

Interviewee S

Have to agree Strongly.

Interviewer

That is very well agreed,

Interviewee S

Yes, what should it know about all the progress reports?

Interviewer

This previous question was the last question for the structured ones.

Now, what do you hope for the EBITDA of COMPANY to increase with EBITDA control in each project in each WBS so that people in the field can determine its profitability? What is your's hope with the implementation of EBITDA with IM4? What's the story, free story?

Interviewee S

Of course, for the lowest WBS, right, maybe if it's a fraction of what's there, right, sir, then it's possible for the one from IM4, it means that what's entered is someone's hours.

Interviewer Yes

Interviewee S

It means that the person-hours are genuinely effective working hours according to the WBS, so to avoid losses, the manager must change the loss of person-hours to be adequate working time. Maybe you are still waiting for the material, and the equipment may still be unserviceable. The manager must record the truth. So if, for example, the effective hours are one day, seven hours, it means that, if it is what is entered to work effectively on WBS, each person must be honest, sir.

Interviewer

Yes, with this daily control, they will also be required to be honest because they control and record it daily.

Interviewee S

Yes, so that, for example, the costs for each project don't overrun because the manager records ineffective hours improvement.

Interviewer

Yes, yes, what are your expectations with the implementation of this EBITDA Project Management? What are your expectations for the COMPANY company's profitability? Do you hope to be better or worse?

Interviewee S

Of course, it has to be better because we control every movement, every business, and every project must generate profit. We should not let the cost of goods sold exceed existing revenue, so revenue must be greater than production costs.

Interviewer

Is there anything else that you want to convey? So that the implementation of the EBITDA will bring benefits to the company. What are your suggestions, and what are your hopes?

Interviewee S

I want the budget secured from the start for the project contract that has already been signed. With such a budget, the planned costs consumed to work on a project should be sufficient. The funding is adequate for the project from the beginning, so the margin must be as maximized as possible. So, the production process will not experience cost overrun. For example, it is the margin that has been secured from the start. The management should determine the costs and consider the possibility of cost increases so that the margin is safe. It always means that every project should be planned to be profitable.

Interviewer

Is there anything else moms want to say?

Interviewee S

Enough, hopefully, each project can generate large profits.

Interviewer

OK, madam, let me turn off the recording first.

INTERVIEW INTERVIEWEE T

Interviewer

Thank you, INTERVIEWEE T, for coming. Let us have a relaxed discussion. So, this is a questionnaire and structure in the interview and unstructured which is intended to make a case study. So, the definition of COMPANY is a project-based company or project-based corporation. Therefore, the COMPANY needs to improve EBITDA project management. We conducted this survey to understand project management and the application of EBITDA in the information technology system that we use in Earned Value Management. The explanation is that project management is project management managed by COMPANY. Then Earned Value Management is a system implemented for project management at COMPANY, then your WBS already knows the Work Breakdown Structure, then Work Packages are WBS children, then EBITDA is Earning Before Interest Tax Depreciation and Amortizations, and EBITDA margin is EBITDA divided by income. In structured interviews, we use 7 Likert scales, one strongly disagrees, the second does not agree, the third agrees, the fourth is in the middle/neutral, the fifth agrees, the sixth agrees, and the seventh strongly agrees. So, shall we begin, sir?

INTERVIEWEE T

Yes, sir.

Interviewer

EBITDA is an appropriate measure of project management profitability because EBITDA is operational profitability before payments of interest, taxes, depreciation, and amortization. Therefore, the value of EBITDA is not affected by Payments of interest, taxes, depreciation, and amortization. The value of EBITDA is only affected by the efficiency and productivity of project management. Mongo's income from one to seven, please

INTERVIEWEE T

I totally agree, sir, because the priority of this project that shouldn't have been influenced by earlier financial factors in the form of taxes and bank interest; that's right, this is what is generated from the business of how to manage a project, that's it, so that's what I think that this measure of the quality of project management using EBITDA is very precise, sir.

Interviewer

Thank you, sir. We continue to number two; EBITDA is an appropriate measure of project management profitability because EBITDA is entirely under the control of the Project Manager and the team, starting from engineering, procurement, logistics, production operations, and warranty delivery. Your opinion?

INTERVIEWEE T

Yes, I totally agree, because earlier, sir, the Project Manager should not be influenced by interest, interest, or taxes, right? That is, it; what does it produce operationally? Is this a measure of quality with EBITDA? So totally agree, sir.

Interviewer

OK, sir, agree. Then point three, companies can integrate EBITDA into EVM by setting revenue so that the authorized budget is not a cost plan but is Company's revenue plan. Then with Company's revenue plan from the budget from Airbus, we then insert the EBITDA first, so the plan cost is the budget minus the new EBITDA, we get the plan cost, so the plan cost is different from the budget will it run out, do you think?

INTERVIEWEE T

I agree, sir. So, we set the authorized budget first as an income plan from it; yes, that must be determined first. Then we set the EBITDA. What is the margin, right? So, we must control the plan costs; the costs must be below that to get the margin. That is right. If what Interviewer mentioned earlier is correct, according to what was planned, what was actualized was zero.

Interviewer

All right, sir, let us go to number four. Companies can integrate EBITDA into EVM by setting a target EBITDA margin in the project. Then the EBITDA margin target is lowered to Work Breakdown Structure, Then Work Packages down to the bottom. What do you think, sir?

INTERVIEWEE T

Totally agree, yes. So earlier, if we looked at it from above, the WBS margins had already been set, right? Well, even below, you also must follow that. Do not let what is called the term adjust to the one above, too, so later on, the packages don't have margins like that; now, this is very important that every package has to have margins because if we know that in COMPANY, there is a name Jipno, right? It contains plan costs, that's plan costs. So, if the margin has been set, the plan cost must be controlled; it cannot be more than the margin, so you can still get EBITDA, sir.

Interviewer

OK, sir, thank you now, point five. Companies benefit from using EBITDA as a measure of project management profitability because EBITDA is a standard measure of financial profitability; thus, if there is an EBITDA margin of EBITDA in the project, non-project managers can know INTERVIEWEE T can tell engineering can tell if not they have to Take part in calculating indicators from their project is difficult in your opinion, what do you think?

INTERVIEWEE T

Yes, I really agree. So what with this EBITDA being translated into operations, right, so that every employee who worked on that earlier was assigned in if we had the name Work Packages earlier, right, now you know that I worked on these Work Packages, how much EBITDA actually is, so he has to know what you are those who are doing it must also know that all this time the only ones who know about the cost are that

Interviewer

Then friends in logistics can also know, sir, with the benefits, what are the benefits, like IT, you know, in Sumatra, too.

INTERVIEWEE T

Yes, so everyone will try to achieve the margin that was set in the EBITDA earlier so that everyone tries to control costs, so they control it, so they are aware of costs; yes, awareness of these costs is fundamental, so if there is no EBITDA, yes earlier maybe it will achieve according to what it's called, right, I'm not aware of it, because I don't know how much the revenue is, what's the difference in EBITDA, how much is that just the cost, so it doesn't bite enough, yeah, if you're not given a challenge, what's your margin? That is, it.

Interviewer

Now, number six, companies can benefit from EBITDA users as a measure of profitability because the Project Manager, Painter, also knows his team knows their EBITDA targets and how to achieve them. According to you?

INTERVIEWEE T

Yes, I totally agree-something like the one we set up earlier. Yes, the project manager and team know the target margin they are working on and how much EBITDA is, so all efforts will be aimed at streamlining the process, right? If the process is efficient, the costs will be lower, so the margins will be higher, right? So that is the main goal, so it is clear what I am streamlining the process to achieve higher margins. That is, it, so I totally agree.

Interviewer

Next, to increase support from supporting units, the Project Manager must share information and report what the targets are and how many achievements are made to the supporting units so that sporting units can support the increase in EBITDA, according to you.

INTERVIEWEE T

Yes, I really agree. So even though the supporting unit also contributes to the increase in EBITDA, for example, for example in friends in logistics, well, logistics friend, he must be able to support him to achieve that by looking for efficient suppliers, yes, with efficient suppliers, maybe the material will be cheaper, right? So, they know my contribution to EBITDA is that if I am looking for an efficient supply chain, which is good, so I totally agree, OK?

Interviewer

Then the structure of this last interview is to make it easier for the Board of Directors to make timely strategic decisions to the Project Manager. So, the project manager must also report his targets and achievements to the BOD to make strategic decisions that are sometimes beyond his Project Manager's ability; what do you think?

INTERVIEWEE T

Yes, I totally agree, sir, because earlier that the Project Manager controls project management, right? If we take the example of the NC212 program, right? That's what you need to know, what is the EBITDA target, so the Board Of Directors will support the Project Manager to achieve the EBITDA that has been targeted because the EBITDA is indeed the translation of finance for this operation to control the project management, so it hasn't been mixed up with interest, yes, with taxes, that's what is a pure performance from the Product Manager Very totally agree.

Interviewer

Now, sir, tell me freely what the benefits of EBITDA are being integrated with EVM and then integrated again with IT for such projects at COMPANY, including defense products, rockets, and weapons. Please, sir.

INTERVIEWEE T

Well, in COMPANY, an aircraft project is extensive, so it involves one example, NC212; there are 19,000 work owners 19,000, so we agreed earlier that product management with EVM will be able to control EBITDA, right? to set up daily, the data that is faced is extensive again as much as 19,000. So, IT is currently playing a significant role in how to translate from 19,000, what the cost is, what the EVM is, how much it is, then based on the business process of each business process, what is the name of each Project Manager by simply opening the dashboard, yes, the dashboard of the EBITDA project management is already You can find out, how much did the EVM arrive today, meaning what exactly is the work result of the project being controlled, how much is that, how much does it cost, then how much is the EBITDA margin per day, right, so it can be controlled from how online and real-time is very important, so this information is needed to make a decision on the following process or the next day, what the next day is, do steps need to be taken so that the margin or EBITDA remains within the target, I think so sir.

Interviewer

The use of IT in this summary includes design, quality, delivery, safety, finance, then the schedule, how about your role, sir?

INTERVIEWEE T

It is amazing. Yes, it is true that IT's job is to present information so it can design the information, the information needed earlier. Regarding quality, for example, quality should provide a rejection rate; yes, there is a rejection rate in that process. So therefore, the data was automatic, yes, automatically, and in real-time, real online time, it must be captured by IT to be presented to the decision maker. , so that can be served right away. The cost is the same. Every work order earlier is to accommodate costs, what are direct costs, namely materials, and person-hours. So, if friends in the field do a job based on a work order, there is an indication of how many hours are used and how much material is used. So, all of that will be translated into costs, yes, costs, so that with that cost quality, IT can also present information, right? So the point is that IT is as a service, yes, as a service to present information in the form of a dashboard, so what is it used for, what is it used for, for making decisions, for making decisions earlier, if we look at EBITDA, we control it, EBITDA controls margins, right? So, this is especially important how IT plays a huge role in controlling costs, costs, and delivery, then what is the name, and what is quality? That is, it, sir, maybe his role.

Interviewer

In maximizing EBITDA, we stay in the corridor regarding quality, delivery, and safety constraints. We cannot maximize everything like that. So, sir, you hope that with the use of IT integration of EBITDA with EVM and IT to maximize EBITDA in the existing corridor, what are your expectations at PT?DI, sir.

INTERVIEWEE T

Yes, if we are from IT, of course, if I determine it will be the emergence of const, what is the cost for, the cost for system development, the cost for providing hardware, right? So, where are the benefits? The user uses IT to help with his work, now that's the benefit, so we hope that what we develop costs the benefit will be more significant, yes, especially what was the benefit earlier, oh the user turns out to be controlling EBITDA to maximize EBITDA, so that means the role of IT indirectly also supports to maximize the company's EBITDA. Well, this is what we hope is to maximize the utilization of this IT. And we will also maximize this IT service by implementing SLE, both hardware SLE and service SLE, so that this data is accurate and then the dashboard information is accurate so that decision-making is also more accurate; that is what we hope, sir.

Interviewer

All right, then, sir, according to you, the application of EBITDA integration with Earned Value Management in projects other than the industrial defense can be used anywhere, sir; your experience is in Garuda, in catering, and so on, please.

INTERVIEWEE T

This EBITDA can be used anywhere, sir. Previously, I had worked at Aero Food, namely Garuda catering, yes Garuda catering. Garuda Catering also serves menus that have been designed by Garuda, right? As before, we know that this is the authority for the budget for this automaker, so to get the margin, we must determine how much margin we have first, right?

Interviewer

So, there is a buffer, sir, right?

INTERVIEWEE T

The buffer used to be set the same, so it depends not only on the manufacture of PT. Just DI, if I have experience in Aerofood, it can also work, so they know it delivers one menu like that, right? The menus are different, sir, so there are business and economy class menus, right? Then the area is also different for Jogja-Solo Semarang, just snacks, then for those 2 hours more, there are heavy meals, Nutri meals, so they must know that I am making this menu and delivering it to Garuda planes. They should know, oh, how much is my margin? That is right. What is the margin which will be enthusiastic, right? Rather than me just serving it without knowing the EBITDA, which is it, this is what I thought earlier, right? In my earlier experience at Aerofood, I translate like that.

Interviewer

OK, OK, OK, thank you for this interview, sir. Is there anything else you need, sir, please?

INTERVIEWEE T

Indeed, for EBITDA, yes, every person or employee, especially at COMPANY must understand what it is, and they also have passion for their work. Not only do they do it, it is just related to costs, but there is a target; if this work produces results, how much will the margin contribute with what EBITDA anyway? So, by cultivating that, they will be aware of costs, aware of costs in this sense. If I want to ask for material from the warehouse, of course, there is the correct cost. He knows how much EBITDA margin I support after I use it.

Interviewer

There is no ownership, sir; there is no sense of ownership.

INTERVIEWEE T

That is right, so they understand that EBITDA should have higher ownership of their work. Well, he has the passion to achieve the targeted EBITDA. That is, it, sir, is related to culture.

Interviewer

Yes, yes, sir, it looks like our interview is over. I will stop recording, OK, sir?

INTERVIEWEE T

Thank You
